# f-SPION-mediated magnetic stimulation induces reparative Schwann cell reprogramming via cytoskeletal dynamics - gated activation of Piezo1

**DOI:** 10.1186/s12951-026-04105-x

**Published:** 2026-02-24

**Authors:** Ting Liu, Mingxi Yang, Wantao Tian, Jingyan Ren, Laijin Lu, Yang Wang

**Affiliations:** 1 Department of Geriatrics, The First Hospital of Jilin University, Changchun, 130021 P. R. China; 2 Department of Hand Surgery, Orthopedic Center, The First Hospital of Jilin University, Changchun, 130021 P. R. China; 3 Key Laboratory of Tissue Repair, Reconstruction and Regeneration of Jilin Province, Changchun, 130021 P. R. China

**Keywords:** Peripheral nerve injury (PNI), Repair Schwann cells (rSCs), Reprogramming, Mechanobiology, Magnetomechanical neuromodulation, Mechanotransduction, Bio-targeted functionalized SPIONs (f-SPIONs), Cytoskeleton dynamic, Mechanosensitive ion channel Piezo1, Calcium influx

## Abstract

**Background:**

The remarkable intrinsic regenerative capacity of peripheral nerves following injury is largely attributed to the phenotypic plasticity of Schwann cells (SCs) and their ability to transition into a repair-supportive state (rSCs). Transcriptional reprogramming of SCs into this reparative phenotype is pivotal for facilitating successful nerve regeneration. While traditionally considered a biochemically regulated process, recent advances in mechanobiology have underscored the crucial role of mechanical cues in modulating SC behavior and gene expression. In this study, we sought to develop a magnetically actuated mechanical stimulation platform based on biotargeted magnetic nanoparticles and a custom-engineered gradient magnetic field, enabling the engineering control of SC reprogramming

**Results:**

We designed and synthesized fluorescent superparamagnetic iron oxide superparticles (f-SPIONs) with specific biotargeting affinity for the actin cytoskeleton, thereby enhancing the spatial precision of nanomagnetic force delivery. In parallel, we engineered a gradient magnetic field generator based on electromagnetic principles to achieve high temporal resolution in magnetic stimulation. By combining f-SPIONs with the external magnetic field, we developed a magnetomechanical stimulation platform capable of remotely delivering noninvasive, high-spatiotemporal-resolution force to SCs and peripheral nerve tissues. Upon magnetic stimulation, SCs exhibited robust reprogramming toward a reparative phenotype, effectively enhancing sciatic nerve regeneration in a rat model. The study of the mechanotransduction mechanism of this phenomenon revealed that f-SPION-mediated magnetic stimulation activated actin cytoskeletal dynamics, gated the opening of mechanosensitive ion channel Piezo1 and triggered calcium influx, ultimately inducing rSC reprogramming

**Conclusions:**

Schwann cells are highly sensitive to external mechanical environments and are capable of transducing mechanical cues into intracellular biochemical signals, thereby modulating their functional state in response. The “magnetomechanical neuromodulation” strategy, developed through the integration of magnetic nanomaterials and externally applied magnetic fields, represents a promising approach that offers innovative mechanotherapeutic tools and perspectives for biomedical research and the treatment of peripheral nerve injuries

**Graphical abstract:**

**Figure:**
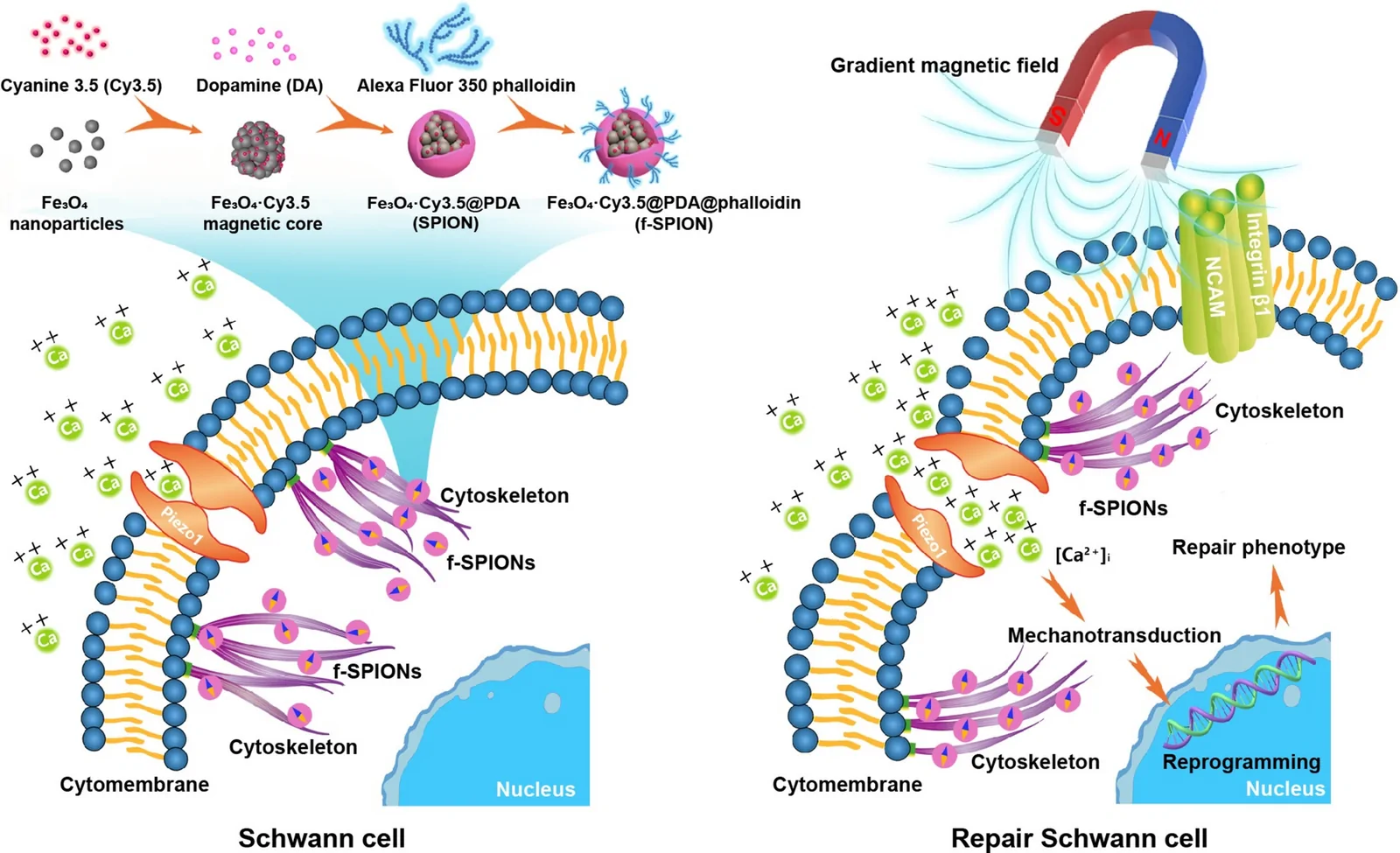

**Supplementary Information:**

The online version contains supplementary material available at https://doi.org/10.1186/s12951-026-04105-x.

## Background

Peripheral nerve injury is a prevalent clinical condition that results in the loss of motor, sensory, and autonomic functions in the affected limb. Despite notable advancements in surgical interventions, clinical outcomes remain suboptimal [1–3]. These injuries trigger a well-characterized cascade of molecular and cellular events at both the proximal and distal nerve segments. Axonal disruption induces functional status changes within the neuronal soma, leading to the activation of regeneration-associated genes [4, 5]. In the distal segment, axons undergo Wallerian degeneration, infiltrating macrophages clear myelin and axonal debris, and Schwann cells adopt a repair-supportive phenotype. Subsequently, regenerating axons must traverse the distal nerve stump to reinnervate target tissues, with remyelination mediated by Schwann cells. Facilitating axonal regeneration across nerve gaps remains a principal therapeutic strategy. Therefore, elucidating the interactions between regenerating axons and their microenvironment is crucial for optimizing functional recovery.

Schwann cells (SCs), the primary glial cells of the peripheral nervous system, ensheathe axons with myelin, which is vital for saltatory conduction and preservation of axonal integrity [6, 7]. SCs exhibit remarkable plasticity and, upon nerve injury, transdifferentiate into a specialized repair-promoting phenotype. These transdifferentiated cells, termed repair Schwann cells (rSCs) [8, 9], are phenotypically distinct from both myelinating and nonmyelinating (Remak) Schwann cells. Following injury, rSCs populate the distal nerve stump to support neuronal survival, secrete biochemical and topographical cues to facilitate axon regeneration, activate autophagy for organized clearance of myelin debris, and form regeneration tracks known as bands of Büngner to guide axons toward their targets [10–14]. The transition to the repair phenotype involves dedifferentiation from the myelinating state and the activation of a distinct transcriptional program—a process referred to as SC reprogramming [9, 15, 16].

The regenerative capacity of peripheral nerves critically depends on the intrinsic plasticity of SCs and their ability to transition into repair phenotypes following injury [9]. Successful nerve regeneration hinges on the SC response to injury, and accordingly, rSCs have emerged as a focal point in regenerative neuroscience research. However, the rSC phenotype is transient and inherently unstable—initially adapting to meet the acute needs of injured nerve tissue but gradually losing its axon-supportive capacity over time [9, 17, 18]. This phenotypic decline is recognized as a major contributor to regenerative failure in human peripheral nerve injuries [12, 15, 19–23]. Therefore, both the induction and sustained maintenance of the rSC phenotype are essential for effective nerve regeneration. Deciphering the intracellular signaling cascades governing this phenotypic transition, alongside the development of cell-based therapies and neuromodulatory strategies, remains a central challenge and an active frontier in regenerative medicine [24–26].

Nerve regeneration has traditionally been considered a biochemically driven process; however, emerging insights from mechanobiology reveal that mechanical forces also play a pivotal role in neural tissue development and regeneration [27–32]. Mechanical and biochemical signals not only interact but can also be transduced across modalities, establishing a complex bidirectional communication system [33–37]. The interplay between physical mechanical cues and neural cell fate determination has rapidly expanded in focus in contemporary neuroscience. Recent studies have demonstrated that mechanical stimulation robustly modulates SC functional phenotypes and their associated gene expression programs [38–40]. The development of neuromodulation technologies capable of delivering precisely controlled mechanical stimuli to SCs and nerve tissues in vivo represents a highly valuable therapeutic strategy. Superparamagnetic iron oxide nanoparticles (SPIONs) provide a promising biomedical platform for this purpose, functioning as transducers that convert external magnetic energy into localized mechanical forces within neural tissues. When exposed to a gradient magnetic field (GMF), SPIONs enable magnetic actuation that modulates the regenerative behavior of SCs. Magnetomechanical actuation refers to the process by which magnetic nanoparticles (MNPs) generate mechanical forces that influence and regulate cellular functions and phenotypic behaviors.

In this study, we engineered actin-targeting superparamagnetic iron oxide nanoparticles (f-SPIONs; Fe₃O₄·Cy3.5@PDA@phalloidin) and established a magnetic actuation system by integrating these nanoparticles with a gradient magnetic field for both in vitro cellular experiments and in vivo animal models. Our results demonstrate that SCs are capable of sensing and responding to magnetically modulated mechanical microenvironments, in which spatiotemporally controlled magnetomechanical forces mediated by f-SPIONs activate actin cytoskeletal remodeling. This mechanotransductive cascade subsequently activates Piezo1 ion channels, initiates intracellular Ca^2^⁺ flux, induces reprogramming toward the rSC phenotype, and ultimately facilitates peripheral nerve regeneration. This study provides a comprehensive conceptual framework for elucidating how mechanical state transitions precisely regulate cellular and tissue functions, offering novel insights and tools for advancing biomedical research and therapeutic strategies for peripheral nerve injuries.

## Materials and methods

### Cell culture and experimental groups

RSC96 cells, a rat-derived Schwann cell (SC) line representing glial cells of the peripheral nervous system, were obtained from the Cell Bank of the Chinese Academy of Sciences (Shanghai, China). The cells were maintained in Dulbecco’s modified Eagle’s medium (DMEM) supplemented with 10% fetal bovine serum (FBS) and 1% penicillin–streptomycin (100 U/mL penicillin and 100 μg/mL streptomycin). To assess the effects of magnetic actuation on SC repair phenotype reprogramming, RSC96 cells were seeded onto imaging-compatible ibidi µ-dishes (ibidi, Cat. #80156, Martinsried, Germany).

For in vitro experiments, distinct experimental groups were established on the basis of specific research objectives. To evaluate the influence of magnetic actuation induced by the interaction between f-SPIONs and a GMF on SC repair phenotype reprogramming, the following groups were defined: (1) Normal control group (designated “Normal”): RSC96 cells were cultured under standard conditions without any treatment. (2) Magnetic stimulation group (designated “f-SPIONs + GMF”): RSC96 cells were incubated with f-SPIONs (15 μg/mL) 24 h after seeding, allowing nanoparticle internalization via endocytosis. The cells were subsequently subjected to magnetomechanical stimulation by placement into a GMF system, enabling f-SPION–GMF interactions. (3) f-SPIONs control group (designated “f-SPIONs”): cells were treated with f-SPIONs (15 μg/mL) but not exposed to the GMF. (4) Magnetic field control group (designated “GMF”): cells were directly exposed to the GMF in the absence of f-SPION-mediated magnetization.

To evaluate the role of actin cytoskeletal dynamics in modulating SC repair phenotype reprogramming, the following experimental groups were established: (1) Magnetic stimulation group (designated “f-SPIONs + GMF”): cells treated with f-SPIONs and exposed to GMF. (2) Cytochalasin D treatment group (designated “Cyto D”): cells were treated with 5 μM cytochalasin D (Cyto D, Invitrogen, Cat. #PHZ1063, Carlsbad, CA, USA) for 45 min to inhibit actin filament polymerization and disrupt cytoskeletal dynamics. (3) Magnetic stimulation combined with Cyto D treatment group (designated “f-SPIONs + GMF + Cyto D”): cells were first subjected to magnetomechanical stimulation via the f-SPION–GMF interaction, followed by treatment with Cyto D (5 μM) for 45 min to inhibit cytoskeletal remodeling. (4) Magnetic stimulation combined with anisomycin treatment group (designated “f-SPIONs + GMF + Anisomycin”): cells were initially exposed to magnetomechanical stimulation via the f-SPION–GMF interaction, followed by 0.2 μM anisomycin treatment (MCE, Cat. #HY-18982, Monmouth Junction, NJ, USA) for 12 h to inhibit protein synthesis-dependent responses.

To investigate the role of mechanosensitive Piezo1 ion channel activity in SC repair phenotype reprogramming, we established the following experimental groups: (1) Magnetic stimulation group (designated “f-SPIONs + GMF”): cells treated with f-SPIONs and exposed to GMF. (2) GsMTx4 treatment group (designated “GsMTx4”): RSC96 cells were treated with 10 μM GsMTx4 (MCE, Cat. #HY-P1410A, Monmouth Junction, NJ, USA), a peptide inhibitor of Piezo family mechanosensitive ion channels, serving as a negative control. (3) Magnetic stimulation combined with GsMTx4 treatment group (designated “f-SPIONs + GMF + GsMTx4”): cells were first subjected to magnetomechanical stimulation via the f-SPION–GMF interaction, followed by treatment with GsMTx4 (10 μM) for 2 h. (4) Yoda1 treatment group (designated “Yoda1”): RSC96 cells were treated with 10 μM Yoda1 (MCE, Cat. #HY-18723, Monmouth Junction, NJ, USA), a selective agonist of Piezo1 channels, which served as a positive control.

Detailed Chemical and peptide reagent information can be found in Supplementary Table S1 [see Additional file 1].

### Experimental animal breeding, welfare ethics and experimental grouping

All animal experiments were conducted in strict accordance with the guidelines issued by the National Technical Committee for Standardization of Laboratory Animals. All procedures were approved by the Animal Welfare and Ethics Committee of the First Hospital of Jilin University (Approval No.: 2022-0045), and all efforts were made to minimize animal discomfort. Male Sprague-Dawley (SD) rats (6–8 weeks old, 180–200 g) were obtained from Liaoning Changsheng Biotechnology Co., Ltd. Deep anesthesia was induced via inhaled isoflurane, followed by the intraperitoneal injection of a mixed anesthetic consisting of ketamine (100 mg/kg) and xylazine (10 mg/kg). All animals that underwent surgical procedures received appropriate postoperative analgesia and were monitored daily for signs of distress or complications. The rats were housed under a 12-h light/dark cycle with ad libitum access to food and water. At the end of the experiments, euthanasia was performed via carbon dioxide (CO₂) inhalation in accordance with standard protocols.

To evaluate the effects of f-SPION-mediated magnetic actuation on SC repair phenotype reprogramming and peripheral nerve regeneration, the animals were randomly assigned to three experimental groups: (1) Normal control group (labeled “Normal”): Rats maintained under standard physiological conditions. (2) Crush injury control group (labeled “Crush”): Rats subjected to sciatic nerve crush injury to observe SC denervation responses and spontaneous regeneration. (3) Magnetic stimulation group (labeled “Crush + f-SPIONs + GMF”): Rats were subjected to sciatic nerve crush injury followed by subepineurial injection of f-SPIONs (200 μg/mL, 25 μL) distal to the injury site and then exposed to a GMF (15 min per session, three times daily for 14 consecutive days).

To investigate the effects of magnetic actuation on the repair phenotype of denervated SCs postinjury, distal sciatic nerve segments were harvested from the injury site for analysis. On postoperative day 7, distal sciatic nerve tissue was collected for transcriptomic mRNA sequencing. Additionally, sciatic nerve morphology, motor function, and electrophysiological performance were evaluated on postoperative days 3, 7, and 14 in all three experimental groups.

### Synthesis of f-SPIONs

#### Synthesis of Fe₃O₄ nanoparticles

Oleic acid (OA)-coated Fe₃O₄ nanoparticles (NPs) were synthesized via a thermal decomposition approach [41]. Fe(acac)₃ (2 mmol), OA (6 mmol), oleylamine (OLA, 6 mmol), and 1,2-hexadecanediol (5 mmol) were dissolved in 20 mL of benzyl ether. The mixture was stirred under a nitrogen atmosphere for 15 min, heated to 200 °C at a rate of 20 °C/min, maintained at this temperature for 30 min, and then refluxed at 265 °C for another 30 min. After cooling to room temperature, the Fe₃O₄ NPs were collected magnetically and washed three times with ethanol. The resulting Fe₃O₄ NPs had an average diameter of 5.8 nm.

#### Synthesis of Fe₃O₄·Cy3.5 superparticles

In our previous protocol, Fe₃O₄**·**Cy3.5 superparticles (SPs) were synthesized by dispersing 28 mg of OA-coated Fe₃O₄ NPs in 4 mL of toluene and 12.5 mL of aqueous solution containing 12.5 mg of sodium dodecyl sulfate (SDS) and 3 mg of Cyanine 3.5 (Cy3.5) dye to form an oil-water (O/W) microemulsion. Following toluene evaporation, the resulting Fe₃O₄·Cy3.5 SPs exhibited an average diameter of approximately 50 nm [42, 43].

#### Polydopamine encapsulation and surface modification

The synthesized Fe₃O₄·Cy3.5 SPs were centrifuged and redispersed in Tris buffer (10 mM, pH 8.5). Then, 26 mg of dopamine (DA) monomer was added and stirred under alkaline conditions for 3 h, allowing dopamine to undergo oxidative polymerization and form a uniform polydopamine (PDA) shell on the Fe₃O₄·Cy3.5 SP surface [44]. The resulting Fe₃O₄·Cy3.5@PDA SPs (SPIONs) were collected via centrifugation at 3500 rpm for 5 min and subsequently redispersed in ultrapure water.

#### Surface functionalization with Phalloidin–Alexa Fluor 350

An aqueous solution (5 mL) containing 100 units of phalloidin-Alexa Fluor 350 (Invitrogen, Thermo Fisher Scientific, Cat. #A22281, Waltham, MA, USA) was mixed with 200 μL of Fe₃O₄·Cy3.5@PDA solution (2 mg). The mixture was stirred gently at room temperature for 24 h. The mixture was centrifuged at 3500 rpm for 5 min, and the resulting precipitate was redispersed in deionized water to obtain the final Fe₃O₄·Cy3.5@PDA@phalloidin (f-SPIONs) solution (0.4 mg·20 units/mL).

### Characterization of f-SPIONs

The particle size and core–shell architecture of the f-SPIONs were examined via a JEM-F200 high-resolution transmission electron microscope (HRTEM) operated at an accelerating voltage of 200 kV. The hydrodynamic diameter and polydispersity index (PDI) of the f-SPIONs were measured via dynamic light scattering (DLS) via a Zetasizer Nano-ZS (Malvern Instruments, UK). The magnetic properties of the f-SPIONs were assessed at 300 K via a superconducting quantum interference device (SQUID) magnetometer (MPMS-XL, Quantum Design Inc., San Diego, CA, USA). Optical properties were evaluated via photoluminescence (PL) spectroscopy with an RF-6000 fluorescence spectrophotometer (Shimadzu, Japan). Fluorescence imaging of cells and tissues was conducted via an inverted confocal laser scanning microscope (CLSM, Nikon AXR (Ti2-E), Japan). The Z-axis resolution of confocal imaging was configured for standard high-resolution cross-sectional acquisition (pinhole = 1 Airy unit, z-step = 0.38 μm). Cellular and neural tissue microstructures were observed via transmission electron microscopy (TEM, EP 5018/40, Tecnai Spirit Biotwin, 120 kV, FEI, Czech Republic s.r.o., Netherlands).

### Design and fabrication of magnetic field generators and characterization of magnetic field gradients

**Fig. 1:**
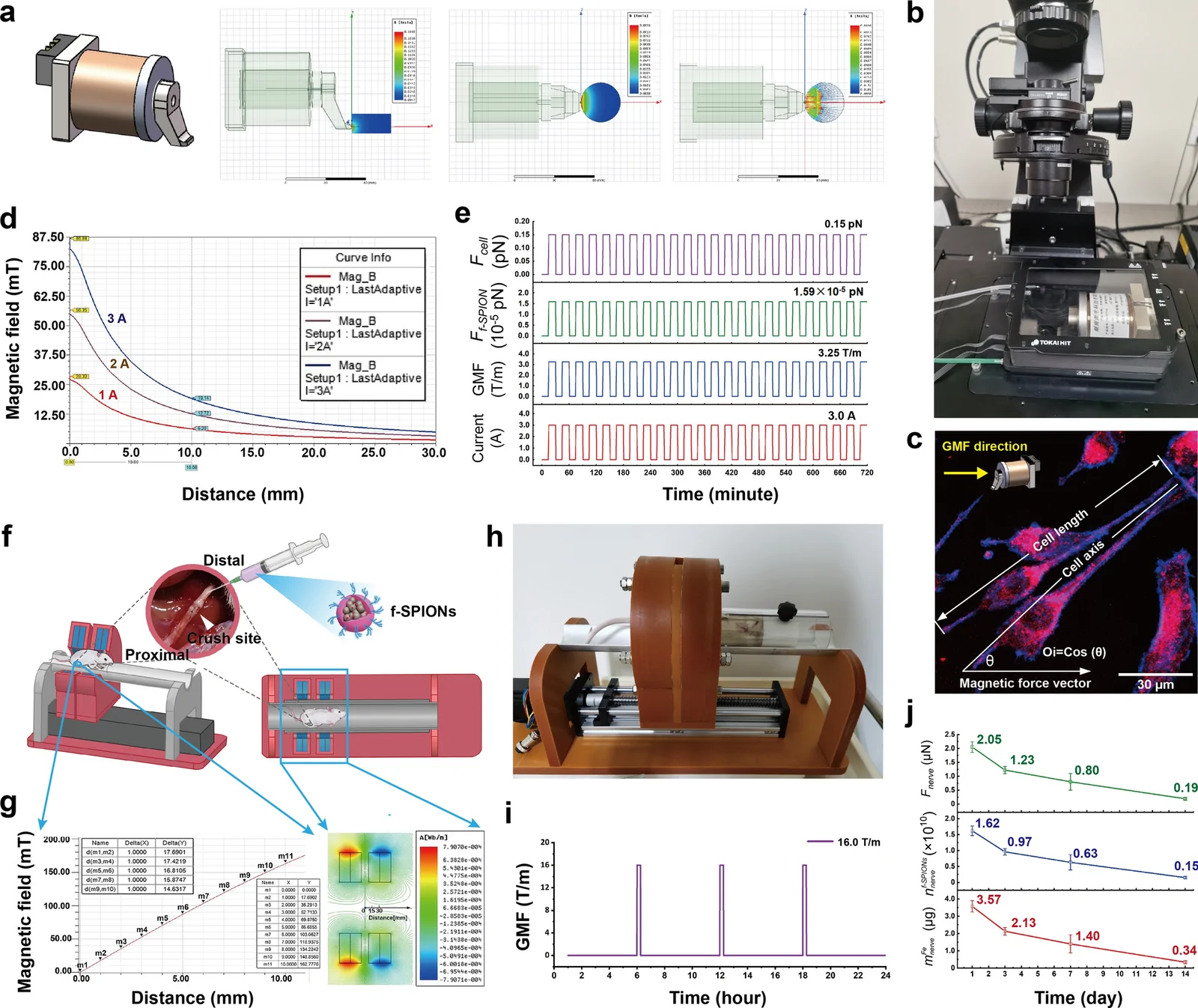
Construction of gradient magnetic field systems and application modes of f-SPION-mediated magnetic force stimulation. **a** For in vitro cell studies, an electromagnetic-based gradient magnetic field generator was developed to produce a static gradient magnetic field targeting the area near the magnetic pole within the culture dish. **b** The culture dish and the magnetic generator were placed inside a CLSM live-cell imaging chamber to enable real-time observation of cells under magnetic stimulation. **c** This system allows continuous, long-term, real-time monitoring of SCs under magnetic stimulation, facilitating assessment of cellular morphology, including cell length and alignment. **d** The gradient strength was tunable by adjusting the input current to the electromagnet (1 A: 1.25 T/m; 2 A: 2.25 T/m; 3 A: 3.25 T/m). **e** The magnetic exposure pattern could be precisely modulated by varying the timing and frequency of current input (15 min ON, 15 min OFF, repeated 24 cycles over 12 h), enabling fine control of f-SPION-mediated nanomechanical stimulation. **f** For in vivo studies, an annular-shaped neodymium magnet system was designed to generate a static GMF targeting the injury site in rat models. **g** The center of the annular magnet array generated a strong static gradient field of 16.0 T/m. **h** Following sciatic nerve crush injury and perineural injection of f-SPIONs, the rats were positioned at the center of the annular magnet daily for magnetic exposure. **i** The exposure protocol involved three sessions per day (6:00, 12:00, and 18:00), each lasting 15 min, for a total of 14 consecutive days. **j** Magnetic force stimulation applied to the sciatic nerve via the combination of f-SPIONs and GMF was assessed throughout the 14-day treatment period. Oi, orientation index; GMF, gradient magnetic field; ***F***_***f-SPION***_, magnetic force exerted on a single f-SPION; ***F***_*cell*_, magnetic force exerted on a single SC; $m_{nerve}^{Fe}$, mass of exogenous Fe within the sciatic nerve; $n_{nerve}^{f - SPIONs}$, number of f-SPIONs within the sciatic nerve; *F*_*nerve*_, magnetic force exerted on the sciatic nerve

To achieve temporally controllable magnetic actuation in vitro, we designed and constructed a compact unipolar electromagnet on the basis of electromagnetic induction principles, which is fully compatible with the inverted CLSM imaging system (Fig. 1a). The electromagnet coil was wound with a 1.1 mm enameled copper wire, featuring an outer diameter of 60 mm, an inner diameter of 20 mm, a length of 110 mm, and a direct current resistance of approximately 1.2 Ω. The electromagnet pole tip measured 2.0 × 2.0 × 1.0 mm (length × width × height) and was fabricated from a 1J50 permalloy. The GMF generator was engineered to fit within the stage-top incubator (TENP&CO₂, TOKAI HIT, Shizuoka, Japan) of the CLSM system (Fig. 1b), enabling real-time cellular imaging during magnetic field exposure (Fig. 1c).

The electromagnet was powered by an external direct current (DC) power supply, with the magnetic field intensity modulated by adjusting the input current. Through a combination of digital magnetic field simulations and empirical measurements via a digital Gauss meter, a static GMF of approximately 3.25 T/m was achieved near the pole tip at an input current of 3 A (Fig. 1d). By varying the current input waveform, both the duration and frequency of magnetic field exposure can be precisely modulated. In this study, the magnetic stimulation protocol involved applying a 3 A current to generate a GMF for 15 min, followed by a 15-min rest period. This cycle was repeated over 12 h for a total of 24 stimulation intervals (Fig. 1e).

For in vivo rat experiments, a GMF generator was custom-built using annular neodymium N48H magnets, as previously described [40] (Fig. 1f). The device comprises four circular neodymium N48H magnets, each with an inner diameter of 70 mm, an outer diameter of 150 mm, and a thickness of 15 mm. Two magnets formed one pair, and two such pairs were spaced 15 mm apart. This configuration generated a gradient magnetic field of up to 16.0 T/m within the central region of the chamber (Fig. 1g). Rats in the magnetic stimulation group were exposed daily to the GMF following sciatic nerve crush injury and subepineurial injection of f-SPIONs (Fig. 1h). The magnetic field exposure was programmed for three sessions per day, each lasting 15 min (scheduled at 6:00, 12:00, and 18:00) (Fig. 1i), and continued for a total duration of 14 days (Fig. 1j).

### Quantitative analysis of f-SPION-mediated magnetic forces

It is well established that a magnetic particle possessing a magnetic moment ***m*** experiences a magnetic force ***F*** in the presence of a magnetic flux density gradient (*∇***B**).

1 $$F = (m \cdot \nabla )B$$

According to this principle, we quantitatively calculated the magnetic force exerted by f-SPIONs on a single SC (***F***_***cell***_) under in vitro electromagnetic field conditions (Fig. 1e) and on the sciatic nerve (***F***_***nerve***_) under in vivo neodymium magnet array field conditions (Fig. 1j). The detailed calculation formulas and derivation procedures are presented in the Supplementary Information [see Additional File 1].

### Microscale thermophoresis assay

Microscale thermophoresis (MST) was conducted via the Monolith NT.115 system (NanoTemper Technologies, GmbH, Germany) to evaluate the binding interaction between f-SPIONs and filamentous actin (F-actin). Phalloidin-Alexa Fluor 350, a commercially available fluorescent probe with high specificity for F-actin, served as the positive control. First, SCs were lysed via a G-Actin: F-Actin In Vivo Assay Kit (Cytoskeleton, Inc., Cat. #BK037, Denver, CO, USA). F-actin was isolated via ultracentrifugation (100,000 × g, 37 °C, 60 min), resuspended in 200 µL of F-actin stabilization buffer (Part #LAS01), and adjusted to a final concentration of 2 mg/mL. F-actin serial dilutions were prepared across 15 concentrations ranging from 2 mg/mL to 0.12 µg/mL. Equal volumes of either f-SPIONs (400 µg/mL) or phalloidin-Alexa Fluor 350 (0.168 µM) were mixed with each F-actin dilution. The mixtures were loaded into premium capillaries (NanoTemper Technologies; ~4 µL per capillary) and analyzed at 25 °C using 20% LED intensity and medium MST power settings. Upon infrared laser activation, F-actin complexes with either f-SPIONs, phalloidin-Alexa Fluor 350 or non phalloidin-conjugated SPIONs (Fe₃O₄·Cy3.5@PDA) migrated toward the cooler periphery of the capillary, and the resulting fluorescence intensity was recorded to determine the binding affinity and calculate the dissociation constant (***K***_***d***_). Data analysis was performed via MO. Affinity Analysis software (version 0.2.2.4). All MST experiments were conducted in triplicate to ensure reproducibility.

### Cell membrane fluorescence staining

To visualize the intracellular internalization of f-SPIONs, RSC96 cell membranes were fluorescently labeled using the Cell Plasma Membrane Staining Kit with DiO (Beyotime Biotechnology, Cat. #C2015, Shanghai, China) with excitation/emission wavelengths of 484/501 nm. RSC96 cells were seeded into confocal imaging ibidi dishes at a density of 5 × 10^4^ cells per dish and cultured under standard conditions for 24 h. The cells were then incubated with 15 μg/mL f-SPIONs for 12 h, followed by three washes with PBS. For membrane staining, 1 mL of staining working solution was added to each dish, consisting of 2.5 μL DiO (400×), 2.5 μL staining enhancer (400×), and 995 μL staining buffer. Cells were incubated at 37 °C for 20 min in the dark. After incubation, the staining solution was removed, and cells were washed three times with PBS. Pre-warmed culture medium (37 °C) was added, followed by Z-stack 3D reconstruction and orthogonal imaging was performed under the CLSM to visualize the internalization of f-SPIONs.

### Actin cytoskeleton staining and tracking

For live-cell staining and tracking of the actin cytoskeleton, procedures were performed according to the manufacturer’s protocol for the CellMask^TM^ Actin Tracking Stain (Invitrogen, Thermo Fisher Scientific, Cat.# A57249, USA). RSC96 cells were seeded into imaging-compatible ibidi µ-Dishes (5 × 10^4^ cells per dish) and incubated for 24 h before receiving the designated experimental treatments. For actin staining, the culture medium was aspirated, and the cells were rinsed three times with phosphate-buffered saline (PBS) to completely remove residual media components. A 1× working solution of CellMask Actin Tracking Stain (Ex/Em: 503/512 nm) was prepared using complete growth medium. A sufficient volume of staining solution was added to ensure complete coverage of the cell monolayer. The cells were incubated at 37 °C in a humidified atmosphere containing 5% CO₂ for 30 min. The cells were subsequently washed four times with prewarmed complete growth medium to remove excess dye. Finally, fluorescence imaging and analysis were performed via CLSM.

For immunofluorescence staining of the actin cytoskeleton, RSC96 cells were rinsed three times with PBS, fixed with 4% paraformaldehyde for 10 min, and permeabilized with 0.1% Triton X-100 for 10 min. Following permeabilization, the cells were blocked with 2% bovine serum albumin (BSA) at room temperature for 1 h. The cells were subsequently incubated with Alexa Fluor^TM^ 350 phalloidin working solution (1 unit per ibidi dish, Ex/Em: 346/442 nm, Invitrogen, Thermo Fisher Scientific, Cat. #A22281, Waltham, MA, USA) at room temperature for 20 min. After two additional washes with PBS, the stained cells were visualized and analyzed via CLSM.

### CCK-8 cytotoxicity assay

RSC96 cells were seeded in 96-well plates at a density of 1 × 10^4^ cells per well and incubated for 24 h at 37 °C in a humidified atmosphere containing 5% CO₂. The cells were subsequently exposed to increasing concentrations of f-SPIONs (5, 10, 20, 50, and 100 µg/mL) for 24 h. After medium removal, the cells were washed three times with PBS and incubated with CCK-8 working solution (Dojindo Laboratories, Cat. #CK04, Kumamoto, Japan) at 37 °C for 2 h. The absorbance at 450 nm (A_sample_) was recorded via a Synergy HT microplate reader (BioTek Instruments, Winooski, VT, USA). Untreated cells served as the control group (the absorbance of which was labeled as the A_control_), and their viability was normalized to 100%. Wells containing only CCK-8 working solution without cells served as blank controls (their absorbance was labeled A_blank_) to eliminate background absorbance from the assay system. Wells without seeded cell, which contained only varying concentrations of f-SPIONs mixed with CCK-8 working solution, were designated the drug control group to eliminate potential interference from the nanomaterial absorbance. The absorbance values were recorded as A_drug_. Cell viability was calculated as a percentage relative to the absorbance of the control group via the following formula:

2 $$\begin{gathered} {\text{Viability (\% )}} \hfill \\ \quad { = }\left[ {{{\left( {{\mathrm{A}}_{{{\mathrm{sample}}}} - {\mathrm{A}}_{{{\mathrm{drug}}}} } \right)} \mathord{\left/ {\vphantom {{\left( {{\mathrm{A}}_{{{\mathrm{sample}}}} - {\mathrm{A}}_{{{\mathrm{drug}}}} } \right)} {\left( {{\mathrm{A}}_{{{\mathrm{control}}}} - {\mathrm{A}}_{{{\mathrm{blank}}}} } \right)}}} \right. \kern-0pt} {\left( {{\mathrm{A}}_{{{\mathrm{control}}}} - {\mathrm{A}}_{{{\mathrm{blank}}}} } \right)}}} \right] \times 100 \hfill \\ \end{gathered}$$

### Reactive oxygen species detection

The levels of intracellular reactive oxygen species (ROS) were quantified via a commercial ROS assay kit (Beyotime Biotechnology, Cat. #S0033S, Shanghai, China). Upon cellular uptake, DCFH-DA is hydrolyzed by intracellular esterases to generate nonfluorescent DCFH, which is subsequently oxidized by ROS to yield fluorescent DCF. DCF fluorescence (Ex/Em: 488/525 nm) was measured to evaluate intracellular ROS accumulation. RSC96 cells were seeded into confocal imaging dishes at a density of 5 × 10^4^ cells per dish and cultured for 24 h. The cells were exposed to different interventions according to their respective experimental groups, followed by three washes with PBS and incubation with DCFH-DA working solution (10 µM) at 37 °C for 20 min. Following an additional three washes with PBS, DCF fluorescence was visualized via CLSM. As a positive control, cells were treated with the ROS inducer RosUp (50 µg/mL) at 37 °C for 30 min to significantly increase intracellular ROS levels, and fluorescence was subsequently visualized via CLSM. RosUp was employed to calibrate and standardize the CLSM excitation parameters for ROS fluorescence detection among different groups, ensuring effective visualization of intracellular ROS-associated green fluorescence.

### Lipid peroxidation assay

Intracellular lipid peroxidation (LPO) levels were assessed via a commercial lipid peroxidation assay kit containing the fluorescent probe BODIPY 581/591 C11 (Beyotime Biotechnology, Cat. #S0043S, Shanghai, China). BODIPY 581/591 C11 is a lipid peroxidation-sensitive fluorescent dye commonly used to detect lipid oxidative stress and antioxidant capacity in live cells. In its reduced form, BODIPY 581/591 C11 exhibits maximum excitation/emission at 581/591 nm and emits red fluorescence; upon oxidation by lipid peroxides, the spectral properties shift to 488/510 nm, resulting in green fluorescence emission. The ratio of red to green fluorescence intensity serves as a quantitative indicator of intracellular lipid peroxide accumulation. RSC96 cells were seeded at a density of 5 × 10^4^ cells per confocal imaging dish and cultured for 24 h. The cells were exposed to different interventions according to their respective experimental groups, followed by three washes with PBS and incubation with BODIPY 581/591 C11 staining solution (2 µM) at 37 °C for 30 min. After two additional PBS washes, the intracellular fluorescence was visualized via CLSM. As a positive control, cells were treated with the LPO inducer LpoUp (1×) at 37 °C for 4 h to markedly increase intracellular lipid peroxidation, followed by BODIPY 581/591 C11 staining and fluorescence imaging via CLSM. LpoUp was used to calibrate and standardize the CLSM excitation parameters for LPO fluorescence detection among different groups, ensuring effective visualization of intracellular oxidized LPO-associated green fluorescence.

### Calcein/PI live/dead cell viability assay

The effect of magnetic stimulation generated by the interaction between f-SPIONs and the external magnetic field on Schwann cell viability was evaluated using the Calcein/PI Live/Dead Viability/Cytotoxicity Assay Kit (Beyotime Biotechnology, Cat. #C2015, Shanghai, China). RSC96 cells were seeded into confocal imaging ibidi dishes at a density of 5 × 10^4^ cells per dish and cultured for 24 h under standard conditions. The cells were then exposed to different interventions according to their respective experimental groups, followed by three washes with PBS to remove residual nanoparticles and serum components. Subsequently, 1 mL of Calcein AM/PI working solution was added to each dish, consisting of 1 μL Calcein AM stock solution (1000×), 1 μL PI (Propidium Iodide) stock solution (1000×), and 1 mL assay buffer. The cells were incubated at 37 °C for 30 min in the dark to prevent photobleaching. After incubation, fluorescence imaging was performed using CLSM equipped with standard filter sets for Calcein AM (Ex/Em: 494/517 nm) and PI (Ex/Em: 535/617 nm) channels. Calcein AM stained live cells exhibiting green fluorescence, while PI stained dead cells exhibiting red fluorescence. All imaging parameters, including exposure time, gain, and laser intensity, were kept identical across all experimental groups to ensure consistency and comparability of fluorescence signals. Quantitative analysis of live and dead cell populations was performed using ImageJ software (National Institutes of Health, Bethesda, MD, USA).

### Flow cytometric analysis

RSC96 cells from each experimental group were harvested and detached using 0.25% trypsin-EDTA, followed by two washes with PBS (pH 7.4). The resulting cell suspension was used for subsequent viability staining. To establish clear discrimination between live and dead cell populations, an apoptosis positive control was prepared by resuspending normally cultured RSC96 cells in the Apoptosis Positive Control Solution provided in the Annexin V-APC/7-AAD Apoptosis Detection Kit (Lianke Bio, Cat. # AP105, Hangzhou, China). The cells were incubated on ice for 30 min and then washed twice with pre-chilled PBS before being mixed with untreated live cells to serve as a positive control. Dead cells were identified using the Live or Dead^™^ Fixable Dead Cell Staining Kit (Green Fluorescence, Ex/Em: 488/525‒540 nm, FITC channel; AAT Bioquest, Cat. #CS22501, USA). Briefly, cells were incubated with the working staining solution at room temperature for 30 min in the dark, followed by two washes with flow cytometry buffer (PBS containing 1% BSA and 0.1% sodium azide). Flow cytometric data were acquired using a CytoFLEX S flow cytometer (Beckman Coulter, Brea, CA, USA) equipped with a 488 nm laser, and analyzed using FlowJo software (version 10.9.0, Becton Dickinson & Company, Franklin Lakes, NJ, USA). Gating of single-cell populations and discrimination of live/dead subsets were performed based on forward (FSC) and side scatter (SSC) characteristics. Detailed flow cytometric gating strategy and analysis procedure are provided in the Supplementary Information [see Additional File 1].

### Fresh-frozen tissue sections of the sciatic nerve

Frozen sectioning coupled with CLSM was employed at defined time points following subepineurial f-SPION injection to assess their spatial distribution and localization within sciatic nerve tissues. The animals were euthanized at 1, 3, 7, and 14 days post-injection, and sciatic nerve segments encompassing the f-SPION injection sites were promptly harvested. Excised nerve tissues were placed into a custom-designed embedding mold (diameter ~3 cm). The samples were embedded in optimal cutting temperature (OCT) compound, stabilized, and rapidly frozen by immersion in liquid nitrogen. Upon complete solidification, the tissue blocks were transferred to a cryostat for cryosectioning. Serial transverse cryosections (10 μm thick) were prepared spanning from the injection site to the distal nerve segments. The cell nuclei were counterstained with 4′,6-diamidino-2-phenylindole (DAPI; Ex/Em: 405/430‒470 nm). CLSM was utilized to evaluate the localization and retention of f-SPIONs (Ex/Em: 561‒568/607 nm) within nerve tissues. Fluorescence images were acquired via a 40× oil-immersion objective lens at 3.2× digital magnification. For ultrastructural validation, TEM was conducted to visualize the presence and subcellular localization of f-SPIONs directly within sciatic nerve tissues.

### Semi-thin and ultrathin sections of the sciatic nerve

Optical microscopy and TEM were employed to examine semi-thin and ultrathin sections for quantitative morphological assessment of sciatic nerve regeneration. At 1, 3, 7, and 14 days post-injury, distal segments of the sciatic nerve were harvested and fixed in a solution of 4% paraformaldehyde (PFA) and 3% glutaraldehyde in 0.1 M phosphate buffer. The tissues were subsequently fixed in 1.5% osmium tetroxide for 90 min, dehydrated and embedded in epoxy resin. Semi-thin transverse sections (5 μm) were cut 10 mm distal to the lesion site via a PowerTome-XL ultramicrotome (RMC, USA), stained with 1% toluidine blue for 2–3 min, and visualized under an optical microscope (IX51, Olympus, Tokyo, Japan). Ultrathin sections (~100 nm) from the same anatomical region were cut via the same ultramicrotome and mounted on 100-mesh copper grids precoated with Formvar. The sections were stained sequentially with saturated aqueous solutions of uranyl acetate and lead citrate. TEM images were acquired and analyzed via ImageJ software (National Institutes of Health, Bethesda, MD, USA). The software facilitated precise quantification of nerve fiber density and classification, as well as measurements of myelin sheath thickness and the G-ratio (axon diameter/fiber diameter) in myelinated axons. For consistency, all nerve crush injuries were induced at the same anatomical location, and tissue sections were collected at an identical distance distal to the injury site across all the animals.

### Transcriptome sequencing and bioinformatics analysis

#### RNA extraction, cDNA library establishment and Illumina sequencing

Total RNA was isolated from cultured cells or nerve tissues via TRIzol reagent (Invitrogen, Thermo Fisher Scientific, Cat. #15596026, Waltham, MA, USA), and its purity, concentration, and integrity were assessed to ensure suitability for downstream applications. Following total RNA isolation, mRNA was enriched via oligo (dT)-conjugated magnetic beads targeting the poly (A) tails and subsequently fragmented into short fragments via fragmentation buffer. First-strand cDNA was synthesized from the fragmented mRNA via random hexamer primers and reverse transcriptase. Second-strand cDNA synthesis was performed via dNTPs, RNase H, and DNA polymerase I. Purified double-stranded cDNA fragments were subjected to end repair, adaptor ligation, and PCR amplification to construct the final cDNA library. Paired-end sequencing (2 × 150 bp) was subsequently conducted on the Illumina NovaSeq^™^ 6000 platform following the manufacturer’s standard protocol. The detailed protocols for library construction and sequencing procedures are available in the Supplementary Information [see Additional file 1].

#### Processing of sequencing data

To ensure the reliability of downstream bioinformatics analyses, raw sequencing reads were subjected to quality control and filtering to generate high-quality clean reads. A total of 75.87 Gbp of high-quality, paired-end reads were successfully obtained after data preprocessing. The raw sequencing datasets have been deposited in the NCBI Gene Expression Omnibus (GEO) under accession numbers GSE305996 and GSE306137. All the clean reads were aligned to the *Rattus norvegicus* reference genome via the HISAT2 aligner (version 2.2.1; https://daehwankimlab.github.io/hisat2/). The detailed procedures for data quality control, read trimming, and filtering are described in the Supplementary Information [see Additional File 1].

#### Differentially expressed gene analysis

Differentially expressed genes (DEGs) analysis was performed via the DESeq2 package for group comparisons, whereas edgeR was utilized for pairwise comparisons between specific sample groups. Genes were classified as differentially expressed if they exhibited a false discovery rate (FDR) < 0.05 and an absolute fold change ≥ 2. Subsequent enrichment analyses of the identified DEGs were conducted to assess their involvement in Gene Ontology (GO) biological processes and Kyoto Encyclopedia of Genes and Genomes (KEGG) pathways.

#### Gene set enrichment analysis

Gene set enrichment analysis (GSEA) was performed via GSEA software (v4.1.0) in conjunction with the Molecular Signatures Database (MSigDB) to evaluate whether predefined gene sets associated with GO terms or KEGG pathways were significantly enriched between experimental groups. Briefly, gene expression matrices were uploaded and ranked on the basis of the Signal2Noise metric for differential expression profiling. Normalized enrichment scores (NESs), nominal p values, and false discovery rates (FDR q values) were computed via default parameters. Gene sets were considered significantly enriched if they met the following criteria: |NES| > 1, nominal *p* value < 0.05, and FDR *q* value < 0.25.

### Assessment of Schwann cell morphology

RSC96 cells were utilized to evaluate the structural remodeling and directional morphological polarization of SCs under magnetomechanical stimulation. The cells were seeded onto ibidi µ-dishes compatible with live-cell imaging systems. After 24 h of incubation, the cells were treated with f-SPIONs (15 μg/mL) and incubated for an additional 2 h to facilitate nanoparticle internalization and cell magnetization. The culture dish and electromagnetic gradient field generator were positioned onto the CLSM stage, which was equipped with a stage-top incubator (TENP&CO₂, TOKAI HIT, Shizuoka, Japan) maintained at 37 °C with 5% CO₂ (Fig. 1b). Magnetic field exposure was programmed in “intermittent pulsed cycle” mode by modulating the input current to the direct current power supply. Each stimulation cycle lasted 30 min, comprising 15 min of 3 A current input to generate a static GMF of 3.25 T/m, followed by 15 min of rest with no current input, allowing the magnetic field to return to baseline (Fig. 1e). A total of 24 pulsed stimulation cycles were administered over a 12-h period (Fig. 1e). CLSM was utilized to examine the morphological effects of f-SPION-mediated magnetic stimulation, including measurement of the longitudinal axis of the cell and the angle θ (0 < θ < π/2) between the cell axis and the magnetic field vector (Fig. 1c). Directional polarization was quantified via the orientation index (Oi), which is defined as Oi = Cos (θ). An angle of θ = 0° indicated complete alignment of the long axis of the cell with the magnetic field vector (Oi = 1), representing maximal morphological polarization along the direction of the magnetic force. Conversely, θ = π/2 denotes a perpendicular orientation, yielding an Oi value of 0 and indicating the absence of directional polarization.

### Quantitative real-time PCR (RT-qPCR) analysis

The cells and sciatic nerve tissue samples from each experimental group were washed three times with ice-cold PBS. Following snap freezing and mechanical homogenization, total RNA was extracted from RSC96 cells and nerve tissues via TRIzol reagent (1 mL per 50 mg of tissue). Phase separation was achieved by adding chloroform, followed by RNA precipitation with isopropanol and subsequent washing with 75% ethanol. Complementary DNA (cDNA) was synthesized via the PrimeScript RT Master Mix Kit (Takara Bio, Cat. #RR036A, Dalian, China) and subsequently subjected to real-time PCR amplification. Quantitative mRNA expression analysis was conducted via the PowerUp SYBR^™^ Green Master Mix Kit (Applied Biosystems, Thermo Fisher Scientific, Cat. #A25742, Waltham, MA, USA). Glyceraldehyde-3-phosphate dehydrogenase (GAPDH) served as the internal housekeeping gene for normalization. All the qPCR primers were synthesized by Invitrogen (Shanghai, China), and their sequences are provided in Supplementary Table S4 [see Additional File 1]. The thermal cycling conditions were set as follows: initial denaturation at 95 °C for 10 min; 40 amplification cycles at 95 °C for 10 s, 60 °C for 30 s, and 72 °C for 1 min; and a final extension step at 72 °C for 10 min. Relative gene expression levels were calculated via the 2^−ΔΔCT^ method. All reactions were conducted in triplicate to ensure reproducibility.

### Western blot analysis

The cells and sciatic nerve tissue samples from each group were washed with PBS and lysed in radioimmunoprecipitation assay (RIPA) buffer (Beyotime Biotechnology, Cat. #P0013B, Shanghai, China). The cell and tissue lysates were collected and centrifuged at 12,000 rpm for 15 min at 4 °C to remove debris. Protein concentrations were quantified via a bicinchoninic acid (BCA) protein assay kit (Thermo Fisher Scientific, Cat. #23225, Waltham, MA, USA). Equal amounts of total protein were separated via 10% SDS–polyacrylamide gel electrophoresis (SDS-PAGE) and transferred onto polyvinylidene difluoride (PVDF) membranes (Millipore, Cat. #IPVH00010, Bedford, MA, USA). The membranes were blocked with 5% nonfat milk in Tris-buffered saline containing 0.1% Tween-20 (TBST) for 1 h at room temperature. The membranes were subsequently incubated overnight at 4 °C with specific primary antibodies: rabbit polyclonal anti-Piezo1 antibody (1:1000, Alomone Labs, Cat. #APC-087, Jerusalem, Israel), rabbit polyclonal anti-Piezo1 antibody (1:1000, Thermo Fisher Scientific, Cat. #PA5-116998, Waltham, MA, USA), rabbit monoclonal anti-NCAM antibody (neural cell adhesion molecule, 1:1000, Abcam, Cat. #ab220360, Cambridge, UK), rabbit monoclonal anti-integrin β1 antibody (1:1000, Abcam, Cat. #ab179471, Cambridge, UK), rabbit monoclonal anti-c-Jun antibody (1:1000, Abcam, Cat. #ab40766, Cambridge, UK), rabbit monoclonal anti-STAT3 antibody (1:1000, Abcam, Cat. #ab68153, Cambridge, UK), and mouse monoclonal anti-GAPDH antibody (1:1000, Proteintech, Cat. #60004-1-Ig, Wuhan, China). After three washes with TBST, the membranes were incubated at room temperature for 1 h with horseradish peroxidase (HRP)-conjugated goat anti-rabbit IgG (H+L; 1:1000, Beyotime Biotechnology, Cat. #A0208, Shanghai, China), followed by three additional washes with TBST. The internal control (GAPDH) membrane was incubated with goat anti-mouse IgG HRP (1:5000, Thermo Fisher Scientific, Cat. #31431, Waltham, MA, USA) following the same procedure. Detailed antibody information can be found in Supplementary Table S5 [see Additional File 1]. The protein bands were visualized via an enhanced chemiluminescence (ECL) detection system (Millipore, Cat. #WBKLS0500, Bedford, MA, USA) and captured with a digital imaging system. Band intensities were quantified via densitometric analysis using ImageJ software (National Institutes of Health, Bethesda, MD, USA). All Western blot experiments were independently performed in triplicate to ensure reproducibility.

### Establishment of a rat model of sciatic nerve crush injury

Following sufficient anesthesia, standard aseptic preparation and disinfection were performed on the lateral aspect of the right hindlimb. A 2.0 cm longitudinal skin incision was made along the posterolateral thigh to expose a substantial segment of the sciatic nerve between the gluteus maximus and quadriceps femoris muscles. The sciatic nerve was crushed twice at the same site—10.0 mm proximal to the bifurcation of the tibial and common peroneal nerves—using fine forceps, with each compression lasting 30 s and a 10-s interval between compressions, resulting in a standardized 2.0 mm-wide crush injury. A 10-0 nylon suture was inserted through the epineurium at the injury site and tied to serve as a marker for precise localization during subsequent procedures. For the administration of f-SPIONs, a 20 µL Hamilton microsyringe was used to slowly inject 25 µL of f-SPION solution (200 µg/mL) into the epineurial space distal to the crush injury site. All surgical manipulations were conducted under a stereomicroscope (Leica Microsystems, Wetzlar, Germany) via standard microsurgical techniques.

### Sciatic functional index analysis

For assessment of the sciatic functional index (SFI), rats were evaluated in a custom-built enclosed walkway measuring 60 cm in length and 10 cm in width, ending in a darkened shelter to encourage continuous forward locomotion. A strip of white paper was affixed to the walkway floor to capture inked hindlimb footprints. All the rats were pretrained to ambulate through the corridor prior to surgical intervention to ensure baseline familiarity and compliance. To obtain footprints, the rats were gently restrained at the torso, and their hind paws were dipped onto an ink-soaked pad containing water-soluble black dye. The rats were then immediately released into the walkway, leaving distinct inked footprints on the paper surface during forward locomotion. Footprint recordings were performed on postoperative days 3, 7, and 14 for quantitative analysis of locomotor functional recovery.

Footprint evaluation was conducted via three parameters: (1) print length (PL), defined as the distance from the heel to the tip of the third toe; (2) toe spread (TS), defined as the span between the first and fifth toes; and (3) intermediary toe spread (ITS), defined as the distance between the second and fourth toes. Each parameter was measured bilaterally on both the experimental limb (E) and the contralateral normal limb (N). The SFI was calculated via the formula proposed by Bain et al. [45]:

3 $$\begin{aligned} {\text{SFI = }} & { - 38}{\mathrm{.3}} \times {\text{(EPL - NPL)/NPL}} \\ & { + 109}{\mathrm{.5}} \times {\text{(ETS - NTS)/NTS}} \\ & { + 13}{\mathrm{.3}} \times {\text{(EIT - NIT)/NIT - 8}}{.8} \\ \end{aligned}$$

Footprint parameters derived from rat gait analysis serve as reliable indicators of hindlimb muscular function [46]. The PL primarily reflects the functionality of the gastrocnemius muscle innervated by the tibial nerve, whereas the TS and ITS indicate the performance of the foot extensor and intrinsic muscles governed by the common peroneal nerve. Following sciatic nerve injury, characteristic footprint alterations include an increase in the PL accompanied by reductions in both the TS and the ITS. Postinjury, the SFI typically yields a negative value, with higher SFI scores indicating better functional recovery of the sciatic nerve. An SFI score near 0 is considered indicative of normal function, whereas a score approaching −100 signifies complete sciatic nerve dysfunction.

### Immunofluorescence staining

For cell immunofluorescence staining, RSC96 cells were rinsed three times with PBS, fixed in 4% PFA for 30 min, permeabilized with 0.1% Triton X-100 for 10 min, and subsequently blocked with 2% BSA at room temperature for 1 h. The cells were incubated overnight at 4 °C with a rabbit polyclonal anti-Piezo1 (Piezo-type mechanosensitive ion channel 1) antibody (1:1000; Alomone Labs, Cat. #APC-087, Jerusalem, Israel) diluted in 0.1% BSA. After washing, the primary antibody was visualized using Alexa Fluor 488-conjugated goat anti-rabbit IgG (Ex/Em: 499/520 nm; 1:2000; Invitrogen, Thermo Fisher Scientific, Cat. #A-11008, Waltham, MA, USA) for 2 h at room temperature. Nuclei were counterstained with DAPI (Ex/Em: 405/430–470 nm).

For gastrocnemius muscle immunofluorescence, rat muscle tissues were dissected, rinsed in PBS, and fixed in 4% PFA at 4 °C for 5 h. The fixed tissues were embedded in paraffin and sectioned via a microtome. The tissue sections were permeabilized with 0.2% Triton X-100 for 15 min and blocked with 10% normal goat serum (1:50; Dako, Agilent Technologies, Cat. #X0907, Santa Clara, CA, USA) at room temperature for 1 h. Immunostaining was carried out via the use of a rabbit polyclonal anti-Laminin beta 1 antibody (1:500; Proteintech, Cat. #23498-1-AP, Wuhan, China). The sections were incubated overnight at 4 °C with the primary antibody, followed by a 2-h incubation at room temperature with Alexa Fluor 488-conjugated goat anti-rabbit IgG (1:200; Invitrogen, Thermo Fisher Scientific, Cat. #A-11008, Waltham, MA, USA). Nuclei were counterstained with DAPI. All washing steps were carried out three times for 5 min each with PBS. Finally, the slides were imaged via CLSM to visualize the muscle microstructure during sciatic nerve regeneration.

### Electrophysiological analysis of the sciatic nerve

At the conclusion of the experimental period, electrophysiological recordings were performed under general anesthesia via a Neuromatic 2000 M/C electromyograph (Dantec Electronic, Skovlunde, Denmark). A pair of low-impedance acupuncture needles (0.25 × 25 mm, <1 Ω) spaced 3 mm apart served as the stimulating electrodes. The electrodes were connected to a data acquisition system, which digitized the analog signals for subsequent software-based analysis. As previously described, bilateral sciatic nerves were surgically exposed under a high-resolution stereomicroscope. A grounding electrode was placed into the quadriceps femoris muscle and connected to the ground terminal of the recording system. For the right hindlimb, the recording electrode was placed in the triceps surae muscle, the reference electrode was positioned in the Achilles tendon, and the stimulating electrode was located proximal to the sciatic nerve crush site. All electrodes were moistened with physiological saline to ensure optimal conductivity. A stimulation intensity of 10 mV was applied to evoke and record compound muscle action potentials (CMAPs). The sciatic nerve was stimulated at two anatomical landmarks: proximally at the sciatic notch and distally at the popliteal fossa. The latency of evoked CMAPs at both stimulation sites was recorded, and the conduction delay between them was calculated. The physical distance between the two stimulation points was precisely measured to calculate the motor nerve conduction velocity (MNCV). Measurements were performed bilaterally on both the experimental (right) and contralateral (left) sciatic nerves.

### In vivo detection of G-actin and F-actin

A G-actin: F-actin In Vivo Assay Kit (Cytoskeleton, Inc., Cat. #BK037, Denver, CO, USA) was used to isolate and extract filamentous actin (F-actin) and globular actin (G-actin) from the various experimental groups. The relative expression levels of F-actin and G-actin were quantitatively assessed via SDS–PAGE followed by Western blot analysis. Briefly, cells were lysed in specialized lysis buffer based on wash buffer, optimized to preserve the native states of both G-actin and F-actin. This buffer selectively solubilized G-actin, while F-actin remained in the insoluble fraction. Ultracentrifugation at 100,000 ×g for 1 h was performed to pellet the F-actin, while the G-actin remained in the supernatant. The supernatant (G-actin) and pellet (F-actin) fractions were subjected to SDS–PAGE, followed by quantitative analysis via Western blotting. The detailed protocols and assay conditions are provided in the Supplementary Information [see Additional File 1].

### Live-cell functional calcium imaging

Time-lapse imaging via CLSM was utilized to monitor the intracellular calcium (Ca^2^⁺) dynamics in SCs. RSC96 cells were loaded with the membrane-permeable calcium indicator Fluo-4 AM (Invitrogen, Thermo Fisher Scientific, Cat. #F14201, Waltham, MA, USA). The cells were incubated at 37 °C for 30 min in Krebs-Ringer buffer (pH 7.4) supplemented with 20% Fluo-4 AM and Pluronic F-127 (Invitrogen, Thermo Fisher Scientific, Cat. #P3000MP, Waltham, MA, USA). Following incubation, the cells were washed twice with Krebs-Ringer buffer and placed onto the CLSM stage for real-time fluorescence imaging. Regions of interest (ROIs) were selected within individual cell, and variations in fluorescence intensity (ΔF) and peak fluorescence (F_max_) were analyzed to assess changes in the intracellular calcium concentration ([Ca^2^⁺]_i_) upon stimulation. Relative changes in ΔF and F_max_ were normalized to the baseline fluorescence (F_₀_). The ratio ΔF/F₀ was calculated as (F_stim_−F_unstim_)/F_unstim_, where F_stim_ denotes the fluorescence intensity after stimulation and F_unstim_ denotes the average baseline fluorescence (F_₀_) before stimulation. Given the typically low levels of free cytosolic Ca^2^⁺ due to sequestration within intracellular organelles, the binding of Fluo-4 to Ca^2^⁺ leads to significant fluorescence enhancement (Ex/Em: 494/506 nm), allowing highly sensitive detection of stimulus-induced increases in cytosolic Ca^2^⁺.

### Statistical analysis

Data distribution normality was evaluated via the Kolmogorov–Smirnov (K–S) test. For continuous variables with a normal distribution—including Western blot protein levels, RT-qPCR gene expression, intracellular iron content, cell viability, Pearson’s R values, nerve fiber density, SFI, CMAPs, and MNCV—the data are presented as the means ± standard deviations, and group differences were assessed via one-way analysis of variance (ANOVA). Post hoc multiple comparisons were performed via the Student–Newman–Keuls (SNK) test when a statistically significant difference was identified.

Kolmogorov-Smirnov tests indicated that the cell length, orientation index (Oi), muscle fiber cross-sectional area, G-ratio of myelinated fibers, cellular fluorescence intensity, and intracellular calcium (Ca^2^⁺) fluorescence intensity were not normally distributed. These variables are expressed as median values with interquartile ranges [Q1–Q3]. Given the violation of normality assumptions, the above parameters were analyzed via the nonparametric Friedman test, followed by Wilcoxon signed-rank tests for pairwise comparisons.

All the statistical analyses were conducted via SPSS software (version 18.0; IBM Corp., Chicago, IL, USA), with a *P* value < 0.05 considered statistically significant.

## Results

### f-SPIONs exhibit favorable physicochemical and biological properties

#### Preparation of f-SPIONs

**Fig. 2:**
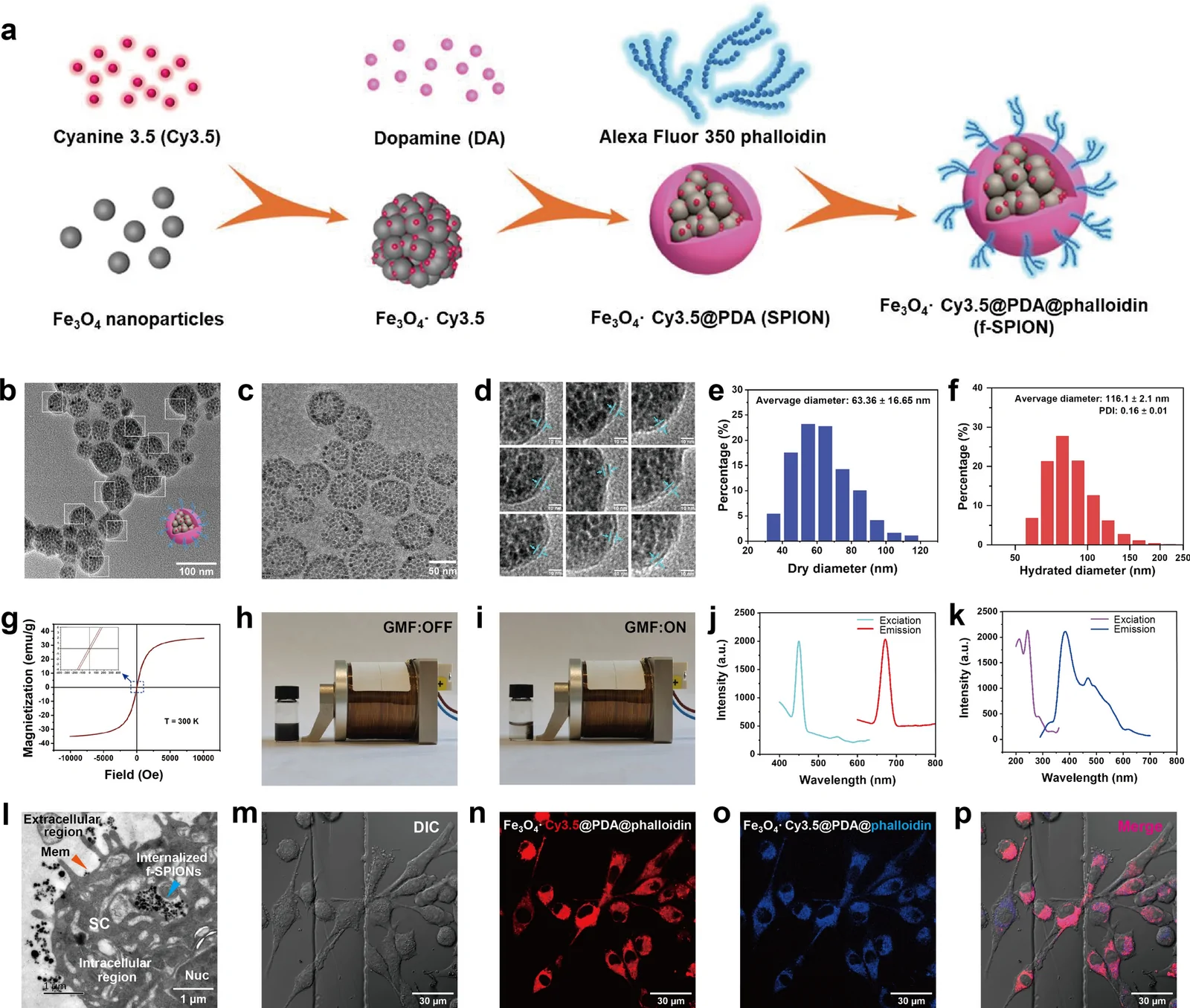
Synthesis and characterization of Fe₃O₄·Cy3.5@PDA@phalloidin superparticles (f-SPIONs). **a** Schematic illustration showing the core–shell architecture and fabrication process of the f-SPIONs. **b** HRTEM image showing the overall structure of f-SPIONs. **c**. HRTEM image of the Fe_3_O_4_·Cy3.5 magnetic core of f-SPIONs. **d** HRTEM image illustrating the outer functionalized shell composed of PDA and phalloidin. **e** Particle size distribution and average dry diameter of the f-SPIONs. **f** DLS analysis showing the hydrodynamic diameter and PDI of the particles in suspension. **g** Magnetic hysteresis loop indicating that f-SPIONs possess strong superparamagnetic behavior and high saturation magnetization (The curve was magnified in the region around the zero magnetizing field). **h** The f-SPIONs remained well-dispersed in solution under nonmagnetic conditions. **i** Upon exposure to a GMF, the f-SPIONs exhibited a rapid and sensitive magnetic response. **j,k** PL spectra confirmed that the particles carried (**j**) red fluorescence derived from Cy3.5 and (**k**) blue fluorescence from phalloidin–Alexa Fluor^™^ 350 conjugates. **l** TEM images demonstrated the efficient cellular uptake of f-SPIONs by SCs via endocytosis. **m–p** Live-cell imaging of SCs after f-SPION internalization, showing (**n**) intracellular red fluorescence from Cy3.5, (**o**) blue fluorescence from phalloidin, and (**p**) strong co-localization of the two signals. *Orange arrow* indicate the cell membrane, *blue arrow* indicate the f-SPIONs internalized by the cells. SC, Schwann cell; Nuc, nucleus; Mem, cytomembrane

As illustrated in Fig. 2a, f-SPIONs were synthesized through a standard multistep protocol, resulting in a core–shell–shell superparticle architecture. Initially, Fe₃O₄ NPs were synthesized via thermal decomposition and subsequently self-assembled with Cy3.5 dye to generate fluorescent–magnetic superparticle cores (Fe₃O₄·Cy3.5). Next, DA underwent polymerization under alkaline conditions to form a PDA shell surrounding the Fe₃O₄·Cy3.5 SPs, yielding Fe₃O₄·Cy3.5@PDA (SPION). Finally, phalloidin-Alexa Fluor 350 was covalently conjugated to the phenolic hydroxyl groups of PDA through a Schiff base reaction, resulting in the final Fe₃O₄·Cy3.5@PDA@phalloidin (f-SPION).

#### Physicochemical Properties of f-SPIONs

HRTEM images reveal that f-SPIONs possess a characteristic core–shell superparticle morphology (Fig. 2b). The Fe₃O₄ NPs, which serve as the fundamental building blocks, exhibited an average diameter of 5.80 nm. The Fe₃O₄·Cy3.5 superparticle cores had an average diameter of 56.60 ± 6.19 nm (Fig. 2c), whereas the PDA/phalloidin coating measured approximately 5.79 ± 1.36 nm in thickness (Fig. 2d). The dry-state diameter of f-SPIONs was determined to be 63.36 ± 16.65 nm (Fig. 2e). DLS analysis of the culture medium revealed a hydrodynamic diameter of 116.10 ± 2.10 nm and a PDI of 0.16 ± 0.01 (Fig. 2f), indicating of excellent colloidal stability and an optimal size for efficient cellular internalization. The synergistic combination of the optimal hydrodynamic size and PDA surface coating facilitated effective endocytic uptake by SCs (Fig. 2l and Fig. S1), thereby enabling subsequent magnetic responsiveness.

#### Magnetic properties of f-SPIONs

The magnetic hysteresis characteristics of the f-SPIONs were evaluated via SQUID magnetometry. As shown in Fig. 2g, the saturation magnetization (***M***_***s***_) at 300 K reached 30 emu/g, accompanied by negligible remanence and coercivity, confirming the superparamagnetic nature of the particles. The intrinsic superparamagnetic properties of the f-SPIONs enabled stable colloidal dispersion in the absence of an external magnetic field (Fig. 2h). Upon exposure to a GMF, the f-SPIONs were rapidly magnetized, with their magnetic moments aligning along the field direction (Fig. 2i). In contrast to ferromagnetic materials, superparamagnetic nanoparticles rapidly lose their magnetization upon removal of the external field, owing to their minimal remanence and coercivity. This field-responsive magnetic behavior enables f-SPIONs to achieve precise temporal control during magnetomechanical stimulation.

#### Optical properties of f-SPIONs

PL spectroscopy was employed to characterize the optical properties of the f-SPIONs. The results revealed distinct red fluorescence originating from Cy3.5, exhibiting an emission peak at approximately 670 nm under 450 nm excitation (Fig. 2j), and blue fluorescence from phalloidin–Alexa Fluor 350, with an emission peak near 386 nm upon 242 nm excitation (Fig. 2k). The presence of both red and blue fluorescence confirmed the successful functionalization of Fe₃O₄·Cy3.5@PDA (SPIONs) with phalloidin–Alexa Fluor 350 (f-SPIONs, Fe₃O₄·Cy3.5@PDA@phalloidin).

To further investigate the fluorescence characteristics of f-SPIONs, live-cell CLSM imaging was conducted in RSC96 cells following uptake of f-SPIONs under the standard Cy3.5 (excitation at 561–568 nm, emission observed at 590–611 nm) and Alexa Fluor 350 (excitation at 350–360 nm, emission observed at 440–460 nm) fluorescence excitation modes (Fig. 2m). The observation of bright intracellular red fluorescence confirmed the efficient uptake of f-SPIONs by RSC96 cells, endowing the cells with magnetic responsiveness (Fig. 2n). We employed plasma membrane staining combined with Z-stack 3D reconstruction (Fig. S2 and Movie S1 [see Additional File 2]) and cross-sectional confocal imaging (Fig. S3), which revealed red fluorescence signals originating from f-SPIONs internalized within SCs. These observations are consistent with the TEM results, further confirming the efficient uptake of f-SPIONs by SCs. Moreover, pronounced blue fluorescence was detected within the cells, confirming the successful surface modification of the SPIONs with phalloidin-Alexa Fluor 350, which conferred it with the ability to specifically bind to F-actin (Fig. 2o). The distinct co-localization of red and blue fluorescence signals further verified the successful surface conjugation of the SPIONs with phalloidin (Fig. 2p). Fluorescence imaging of the normal control and SPIONs control cells in Fig. S4 ruled out the possibility of endogenous cellular autofluorescence, further confirming the reliability of the fluorescence imaging results described above. The stable and robust fluorescence of f-SPIONs enables real-time, in vivo visualization of nanoparticle–cell interactions under dynamic physiological conditions.

#### f-SPIONs demonstrate superior ability to target F-actin

**Fig. 3:**
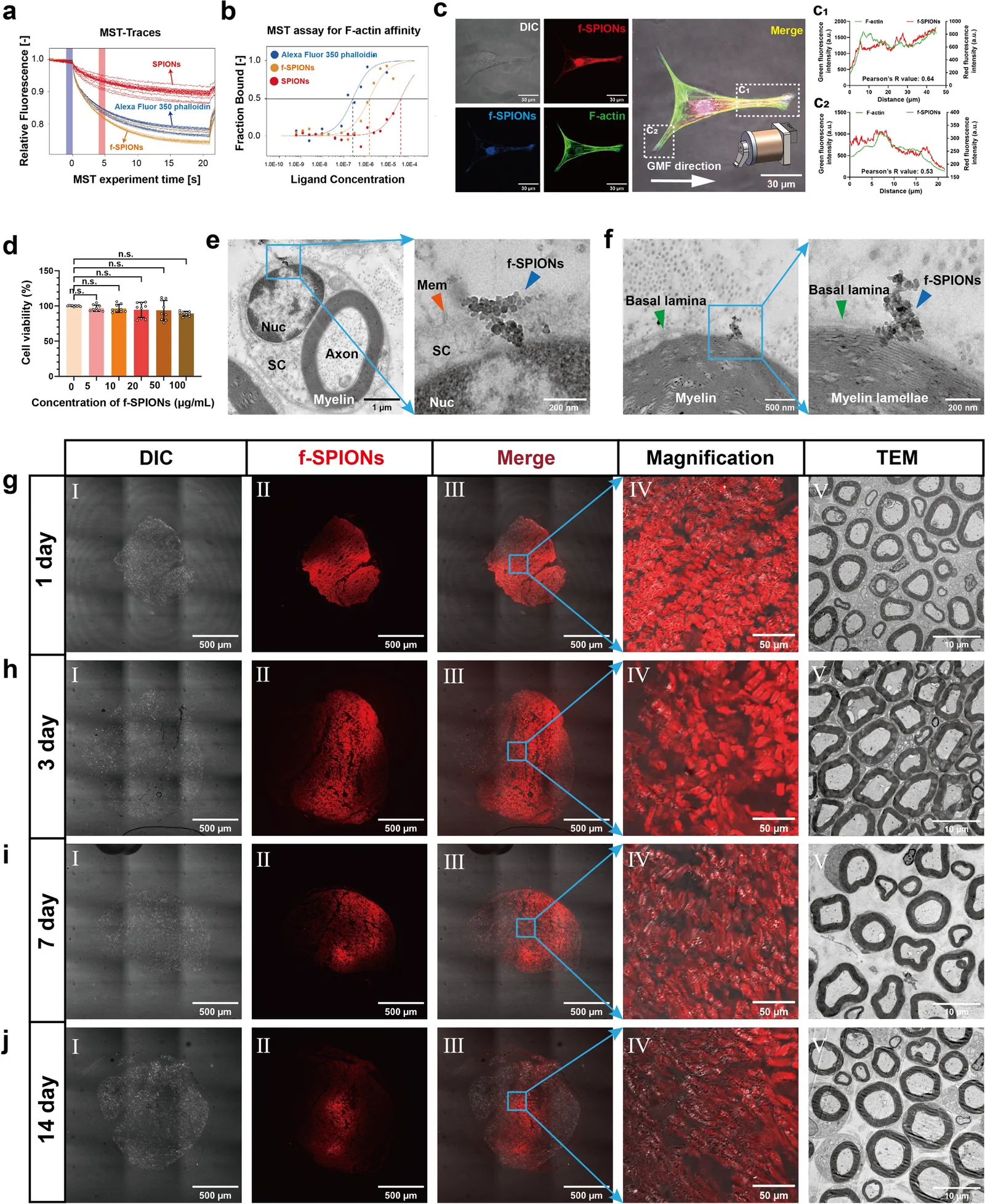
Neuroaffinity and F-actin-targeting capability of f-SPIONs. **a** Relative fluorescence trace changes during MST analysis of the binding affinity between different samples and F-actin. **b** MST assay revealed a high binding affinity between f-SPIONs and F-actin, as evidenced by the calculated dissociation constant (*K*_*d*_). **c** Live-cell actin staining and fluorescence imaging demonstrated distinct co-localization between f-SPIONs and F-actin stress fibers in RSC96 cells, indicating the specific labeling capability of f-SPIONs toward the actin cytoskeleton. (**c**_**1**_) Along the direction of the magnetic field gradient, f-SPIONs displayed higher co-localization with F-actin (Pearson’s R value: 0.64) compared to (**c**_**2**_) the direction perpendicular to the magnetic field (Pearson’s R value: 0.53). **d** CCK-8 cytotoxicity assays confirmed high cell viability after 24 h coincubation with varying concentrations of f-SPIONs. **e,f** TEM images revealed in vivo internalization of f-SPIONs by (**e**) myelinating SCs and (**f**) lamellar myelin structures. **g–j** Fluorescence imaging of fresh-frozen longitudinal sections of rat sciatic nerves at (**g**(**I–IV**)) 1 day, (**h**(**I–IV**)) 3 days, (**i**(**I–IV**)) 7 days, and (**j**(**I–IV**)) 14 days post perineural injection of f-SPIONs combined with magnetic field exposure showed persistent localization and gradual metabolism of f-SPIONs within the nerve tissue. TEM images of sciatic nerve fibers subjected to nanomagnetic stimulation for (**g**(**V**)) 1 day, (**h**(**V**)) 3 days, (**i**(**V**)) 7 days, and (**j**(**V**)) 14 days revealed intact microstructural morphology. Statistical significance: n.s., not significant. SC, Schwann cell; Nuc, nucleus; Mem, cytomembrane

To validate the successful functionalization of the SPIONs (Fe₃O₄·Cy3.5@PDA) with phalloidin–Alexa Fluor 350, their binding affinity to F-actin was quantitatively assessed in vitro via MST. Figure 3a shows the MST fluorescence trace changes upon binding with F-actin in the different experimental groups. The dissociation constant (***K***_***d***_) serves as a quantitative indicator of binding strength, with lower *K*_*d*_ values reflecting higher affinity. As shown in Fig. 3b, the positive control, phalloidin–Alexa Fluor 350, exhibited a *K*_*d*_ of 0.219 ± 0.108 μM for F-actin. f-SPIONs also showed a clear binding affinity toward F-actin, with a *K*_*d*_ of 1.540 ± 0.593 μM. In contrast, the negative control SPIONs displayed a markedly lower affinity (*K*_*d*_ = 44.936 ± 9.481 μM) compared with f-SPIONs, indicating that phalloidin conjugation endowed the superparticles with selective F-actin–targeting capability, thereby enabling efficient cytoskeletal magnetic responsiveness.

To further confirm the F-actin targeting specificity of f-SPIONs, live-cell fluorescence imaging was performed to visualize their co-localization with the actin cytoskeleton in RSC96 cells. As shown in Fig. 3c, upon exposure to a directional GMF, RSC96 cells extended neurite-like protrusions aligned with the magnetic force vector. Within these protrusions, red and blue fluorescence signals from the f-SPIONs preferentially accumulated along the axis of the magnetic field. Notably, green fluorescence from CellMask-labeled actin filaments also realigned along the same axis, forming prominent bundled stress fibers. As shown in Fig. 3c_1_, along the direction of the magnetic field gradient, f-SPIONs displayed higher co-localization with F-actin (Pearson’s R value: 0.64) compared to the direction perpendicular to the magnetic field (Fig. 3c_2_, Pearson’s R value: 0.53). Conversely, in the f-SPIONs control group without a magnetic field (Fig. S5a and S5b) and the SPIONs-mediated magnetic stimulation group (Fig. S5c and S5d), no such spatial-specific co-localization phenomenon was observed. Furthermore, quantitative fluorescence co-localization analysis in Fig. S5e revealed that f-SPIONs exhibited a significantly higher Pearson’s correlation coefficient (Pearson’s R value) with F-actin compared to SPIONs (0.52 ± 0.06 vs. 0.26 ± 0.05, P < 0.001). This suggests that f-SPIONs exhibit spatially targeted distribution within the cell under a magnetic field, enabling the directional application of magnetic force specifically to cytoskeletal structures in defined subcellular regions.

#### f-SPIONs demonstrate minimal cytotoxicity in vitro

To assess biocompatibility, the cytotoxicity of the f-SPIONs was quantitatively evaluated via the CCK-8 assay. RSC96 cells were exposed to increasing concentrations of f-SPIONs (0–100 μg/mL) for 24 h under standard culture conditions. The results of the CCK-8 assay demonstrated that RSC96 cells maintained high viability across the tested concentration range of f-SPIONs (5–100 µg/mL) (Fig. 3d). These findings confirm the excellent cytocompatibility of f-SPIONs, as high cell viability was retained even at elevated nanoparticle concentrations. On the basis of these results, a concentration of 15 μg/mL was selected for in vitro cell magnetization.

To evaluate the potential cytotoxicity and ferroptosis-associated oxidative stress induced by f-SPIONs and magnetic stimulation, the intracellular levels of ROS were measured in RSC96 cells. As shown in Fig. S6, no significant accumulation of ROS or detectable green fluorescence was observed in the normal control group, the f-SPIONs-only control group, or the GMF-only control group. More importantly, no increase in ROS levels was detected in the magnetic stimulation group exposed to the combined treatment of f-SPIONs and GMF.

LPO is a pivotal event in various pathological processes and is a hallmark of ferroptosis. To assess potential cellular damage induced by f-SPIONs and magnetically mediated stimulation, intracellular LPO levels were measured in RSC96 cells. As shown in Fig. S7, BODIPY 581/591 C11 remained predominantly in its reduced form, exhibiting intense red fluorescence, whereas the oxidized form displayed minimal green fluorescence in the normal control group, the f-SPIONs-only control group, and the GMF-only control group. In the magnetic stimulation group exposed to the combined treatment of f-SPIONs and GMF, the red-to-green fluorescence ratio remained comparable to that of the untreated controls, with consistently strong red fluorescence, indicating the absence of LPO accumulation.

As shown in Fig. S8, the Calcein/PI Live/Dead Cell Viability Assay demonstrated that all groups—including the f-SPIONs-only control, the GMF-only control, and the magnetic stimulation group combining f-SPIONs with GMF—exhibited high cell viability (> 97.0%), with no statistically significant difference compared with the normal control group (P > 0.05). To further validate this finding, we performed a quantitative analysis of viable cell rates in the aforementioned experimental groups using flow cytometry. As shown in Fig. S9, highly consistent results were obtained: both the magnetic stimulation group, the f-SPIONs control group, and the GMF control group displayed similarly high cell viability (> 98.0%) relative to the normal control group (P > 0.05). These results further confirm that the concentration of f-SPIONs and the magnetic stimulation system employed in this study possess excellent biocompatibility and exert minimal cytotoxicity toward Schwann cells.

To more intuitively demonstrate the effects of f-SPION-mediated magnetic stimulation on SC viability and physiological activity, RSC96 cells were co-incubated with 15 μg/mL f-SPIONs while being simultaneously exposed to a gradient magnetic field. Long-term time-lapse imaging demonstrated that the cells maintained high viability and exhibited active mitotic activity, further supporting the safety of f-SPION-mediated magnetic stimulation [Movie S2 see Additional File 3]

#### f-SPIONs demonstrate excellent biocompatibility and biosafety in vivo

Following the confirmation of the negligible in vitro cytotoxicity of f-SPIONs, we conducted a systematic evaluation of their in vivo toxicological profile and pharmacokinetics in rats. f-SPIONs were diluted to a concentration of 1 mg/mL in sterile distilled water and administered intravenously via tail vein injection at a dosage of 1 mg/kg body weight. The control animals received an equivalent volume of sterile distilled water. General health status and body weight were monitored for 14 days following injection. On days 1, 3, 7, and 14 post-injection, liver and kidney tissues were harvested for histopathological examination and quantification of iron (Fe) content. The results revealed that no mortality occurred during the 14-day observation period, and all the rats remained in good general condition with steadily increasing body weight (Fig. S10). Histological analysis of the liver and kidney revealed no abnormalities (Fig. S11a and S11b), and ICP-AES revealed no significant exogenous iron accumulation in these organs (Fig. S11d and S11e), confirming the favorable biocompatibility of the f-SPIONs.

To evaluate the biodistribution of intravenously administered f-SPIONs within the nervous system and their potential to cross the blood–brain barrier, rat brain tissues were analyzed. Histopathological examination revealed no evidence of inflammation or tissue damage in the brain sections (Fig. S11c), and ICP-AES analysis confirmed the absence of significant exogenous iron accumulation (Fig. S11f). These findings demonstrate the excellent in vivo biocompatibility and biosafety of f-SPIONs, supporting their further development for localized application within the peripheral nervous system.

#### f-SPIONs demonstrate excellent neural affinity in vivo

Before initiating in vivo nerve magnetostimulation, the uptake and internalization of f-SPIONs into nerve fibers were examined via TEM. A volume of 25 μL of f-SPIONs (200 μg/mL, equivalent to 20% of the systemic dose) was precisely injected beneath the epineurium of the rat sciatic nerve under microscopic guidance. The animals were then subjected to daily exposure to GMF. As shown in Fig. 3e and f, TEM imaging revealed that f-SPIONs crossed the basal lamina of the myelin sheath and were subsequently internalized by SCs (Fig. 3e), where they were distributed within the myelin lamellae (Fig. 3f). These observations confirm the selective uptake and intracellular localization of f-SPIONs within SCs, confirming their strong neural affinity in vivo.

#### f-SPIONs demonstrate excellent stability within neural tissue

We further assessed the in vivo stability and spatiotemporal biodistribution of f-SPIONs within neural tissue. Sciatic nerves were collected at 1 day, 3 days, 7 days, and 14 days following magnetostimulation, and cryosections were examined via CLSM. As shown in Fig. 3g–j, strong red fluorescence signals corresponding to f-SPIONs were observed in cross-sections of the sciatic nerve. The fluorescence intensity gradually decreased over time (Fig. 3h–j); nevertheless, residual red fluorescence remained detectable at 14 days following local epineurial injection (Fig. 3j(Ⅰ–Ⅳ)). These findings suggest that f-SPIONs remain stably localized in neural tissue for up to 14 days before undergoing gradual clearance.

#### f-SPIONs demonstrate minimal neurotoxicity in the peripheral nervous system

After establishing the neuroaffinity and pharmacokinetics of f-SPIONs, we finally investigated the potential neurotoxicity and tissue damage induced by f-SPIONs and their associated magnetic stimulation in vivo. Ultrathin sectioning and TEM imaging were performed at various time points (1, 3, 7, and 14 days) following f-SPION injection combined with magnetic field exposure to further assess the effects of f-SPION-mediated magnetic stimulation on the nerve microstructure. As shown in Fig. 3g(Ⅴ)–j(Ⅴ), no discernible pathological alterations were detected in the microstructure of the sciatic nerve throughout the 14-day period of magnetostimulation. The normal architecture of nerve fibers was preserved, supporting the excellent biocompatibility and minimal neurotoxicity of f-SPIONs under the tested conditions.

### Application of temporally and spatially specific magnetomechanical stimulation via f-SPIONs and gradient magnetic fields

#### In vitro, f-SPION-mediated piconewton (pN)-scale magnetomechanical stimulation of SCs

Under a static gradient magnetic field of 3.25 T/m generated by an electromagnet (Fig. 1d), each f-SPION exerted a magnetic force (***F***_***f-SPION***_) of approximately 1.59 × 10⁻^5^ pN (Fig. 1e). Taking into account the core–shell structure, particle size, and iron content of Fe₃O₄, the iron mass per f-SPION ($m_{f - SPION}^{Fe}$) was estimated to be approximately 2.21 × 10⁻^4^ pg. After 24 h of incubation with f-SPIONs (15 μg/mL), the average intracellular iron uptake per RSC96 cell ($m_{cell}^{Fe} \,$) was determined to be 2.05 ± 0.64 pg via ICP-AES. Dividing $m_{cell}^{Fe} \,$ by $m_{f - SPION}^{Fe}$ yielded an estimated uptake of ~9.30 ± 2.89 × 10^3^ f-SPIONs per cell ($n_{cell}^{f - SPIONs}$). Therefore, following treatment with 15 μg/mL f-SPIONs, SCs were effectively magnetized and subjected to an average mechanical force (***F***_***cell***_) of ~0.15 ± 0.05 pN per cell under a 3.25 T/m magnetic field gradient (Fig. 1e). The detailed calculation formulas and derivation procedures are presented in the Supplementary Information [see Additional File 1].

#### In vivo, f-SPION-mediated micronewton (μN)-scale magnetomechanical stimulation to the rat sciatic nerve

Following subepineurial injection of f-SPIONs (200 μg/mL, 25 μL), ICP-AES analysis revealed that the exogenous iron content in sciatic nerve tissue ($m_{nerve}^{Fe}$) was 3.57 ± 0.33 μg, 2.13 ± 0.22 μg, 1.40 ± 0.52 μg, and 0.34 ± 0.09 μg at 1, 3, 7, and 14 days post-injection, respectively (Fig. 1j). By dividing the $m_{nerve}^{Fe}$ by the $m_{f - SPION}^{Fe}$, the estimated number of residual f-SPIONs per nerve ($n_{nerve}^{f - SPIONs}$) was calculated to be 1.62 ± 0.15 × 10^10^, 0.97 ± 0.10 × 10^10^, 0.63 ± 0.24 × 10^10^, and 0.15 ± 0.04 × 10^10^ at days 1, 3, 7, and 14, respectively (Fig. 1j). Within a 16.0 T/m gradient field generated by an annular neodymium magnet array (Fig. 1g), the magnetic force exerted per f-SPION (***F***_***f-SPION***_) was approximately 1.27 × 10⁻^4^ pN. Consequently, the cumulative magnetic force exerted on the sciatic nerves (***F***_***nerve***_) was estimated to be approximately 2.05 ± 0.19 μN, 1.23 ± 0.13 μN, 0.80 ± 0.30 μN, and 0.19 ± 0.05 μN at 1, 3, 7, and 14 days, respectively (Fig. 1j; see the Supplementary Information for detailed derivations and calculations).

### f-SPION-mediated magnetomechanical stimulation induces rSC reprogramming in vitro

The rSCs are molecularly distinct from myelinating and nonmyelinating (Remak) Schwann cells, as evidenced by their unique gene expression profiles [6, 9–13, 47–52].

#### f-SPION-mediated magnetomechanical stimulation reprograms the gene expression profile of SCs

**Fig. 4:**
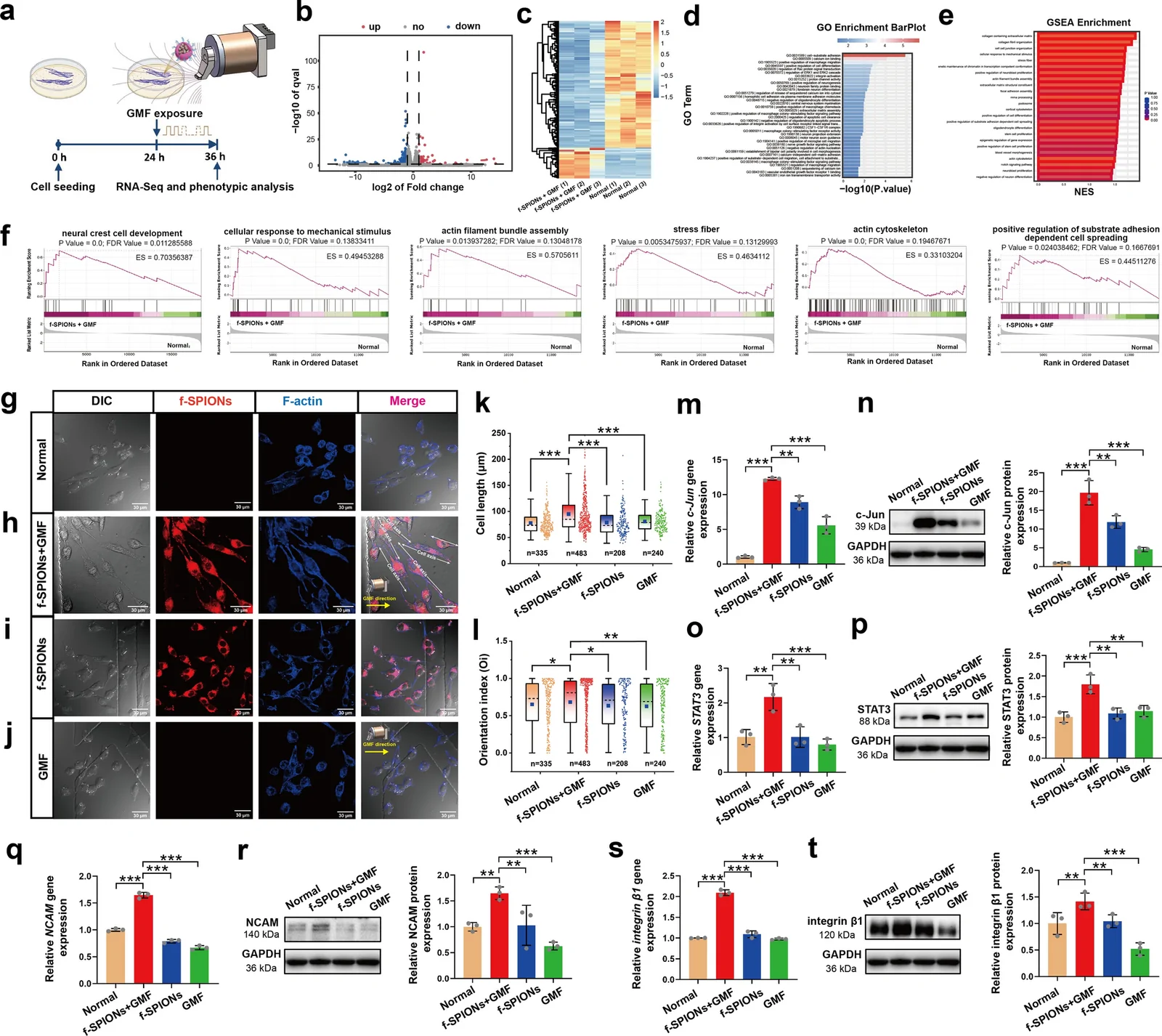
f-SPION-mediated magnetic actuation induces rSC reprogramming in vitro. **a** Schematic workflow illustrating the magnetic stimulation of SCs by the interaction between f-SPIONs and an electromagnetic gradient field. The cells were collected post-stimulation for mRNA transcriptomic sequencing and phenotypic analyses. **b** Volcano plot displaying the global distribution of DEGs between the magnetic stimulation group and the untreated control group. **c** Hierarchical heatmap showing the expression trends of DEGs across the magnetic force-treated and control groups. **d** GO enrichment analysis indicating the biological processes and functions associated with DEGs induced by magnetic stimulation. **e** GSEA of GO terms highlighting the biological pathways and processes differentially regulated under magnetic stimulation. **f** Gene sets related to neural crest development, actin cytoskeleton remodeling, and cellular mechanotransduction were significantly upregulated in the magnetic stimulation group. **g–j** Representative immunofluorescence images showing SC morphology in the (**g**) normal control, (**h**) magnetic stimulation, (**i**) f-SPION-only control, and (**j**) magnetic field-only control groups. **k,l** Quantification of (**k**) cell length and (**l**) orientation index across experimental groups. **m,n**. Analysis of c-Jun transcription factor expression by (**m**) RT-qPCR and (**n**) Western blotting. **o,p** STAT3 expression levels assessed by (**o**) RT-qPCR and (**p**) Western blotting. **q,r** Expression of NCAM evaluated by (**q**) RT-qPCR and (**r**) Western blotting. **s,t** Integrin β1 expression levels analyzed using (**s**) RT-qPCR and (**t**) Western blotting. Data are representative of three independent replicates. “n” indicates the total number of cells measured. *Yellow arrows* indicate the direction of the GMF. Statistical significance: * *p* < 0.05; ** *p* < 0.01; *** *p* < 0.001

To evaluate how magnetomechanical stimulation influences SC phenotypic reprogramming, we conducted mRNA transcriptomic profiling of RSC96 cells following exposure to magnetic actuation. As depicted in Fig. 4a, an in vitro setup was established in which f-SPIONs interacted with a GMF generated by an electromagnet, thereby delivering magnetomechanical stimulation to cultured cells. After administering intermittent pulsed magnetic stimulation (0.15 pN per pulse, every 15 min) for a total duration of 12 h, RSC96 cells were collected for RNA sequencing and phenotypic analysis.

As illustrated in Fig. 4b, a volcano plot was generated to depict the global distribution of DEGs between the magnetically stimulated and untreated normal control groups. A total of 348 genes exhibited differential expression, including 68 upregulated and 280 downregulated genes. Hierarchical clustering visualized via a heatmap further emphasized group-specific gene expression patterns (Fig. 4c). These findings confirm that magnetomechanical stimulation mediated by f-SPIONs elicits pronounced changes in the gene expression profile of SCs. GO enrichment analysis was subsequently conducted to identify biological processes associated with the DEGs. The highly responsive genes were significantly enriched in GO categories associated with SC differentiation, cell adhesion, macrophage chemotaxis, calcium ion regulation, and axon regeneration guidance (Fig. 4d). GSEA was subsequently performed to further investigate how magnetomechanical stimulation modulates SC functional phenotypes (Fig. 4e). Compared with those in normal controls, gene sets related to neural crest cell development, actin cytoskeleton remodeling, and cellular responses to mechanical stimulus were significantly upregulated in the magnetically stimulated group (Fig. 4f). These characteristic changes in gene expression profiles indicate that Schwann cells underwent reparative phenotype reprogramming in response to magnetic stimulation.

#### f-SPION-mediated magnetomechanical stimulation induces SC elongation and branching

A defining function of rSCs is their ability to form regenerative pathways called bands of Büngner, which serve as scaffolds to direct regenerating axons toward their target tissues. Through dynamic cytoskeletal remodeling, rSCs undergo elongation and extend interlacing cellular processes that form compact longitudinal columns—an essential architecture for effective axonal regeneration [9, 15, 16, 53, 54].

As shown in Fig. 4g, RSC96 cells in the normal control group exhibited a typical spindle-shaped morphology with a median cell length of 73.54 [62.87–89.31] μm (Fig. 4k). In contrast, magnetically stimulated RSC96 cells exhibited marked bipolar elongation and extensive branching, forming conspicuous neurite-like extensions (Fig. 4h), with a significantly longer median length of 85.15 [70.71–111.98] μm (Fig. 4k). In contrast, RSC96 cells treated with f-SPIONs alone (Fig. 4i) or exposed solely to the magnetic field (Fig. 4j) failed to exhibit the pronounced structural remodeling observed under combined magnetomechanical stimulation. These cells retained a spindle-shaped morphology comparable to that of untreated controls, with median lengths of 73.10 [60.70–92.60] μm and 79.00 [66.69–92.46] μm—both of which were significantly shorter than those of magnetically stimulated cells (Fig. 4k).

In addition to assessing cell length, we quantified the orientation index (Oi) to evaluate cellular alignment under different conditions. As shown in Fig. 4l, cells in the normal control, f-SPION-only, and magnetic field-only groups presented comparable Oi values of 0.73 [0.44–0.93], 0.71 [0.39–0.92], and 0.69 [0.35–0.93], respectively. In contrast, magnetically stimulated cells presented a significantly elevated Oi of 0.81 [0.41–0.96], reflecting preferential alignment of RSC96 cells along the direction of the GMF during f-SPION-mediated actuation. Movie S2 dynamically illustrates the directed extension of neurites and lamellipodia in RSC96 cells under targeted magnetic stimulation [see Additional File 3].

Collectively, these findings confirm that f-SPION-mediated magnetomechanical stimulation triggers profound structural remodeling in RSC96 cells, promoting elongation, neurite-like outgrowth, and spatial alignment along the magnetic field gradient. This structural polarization is recognized as a hallmark of the repair-supportive phenotype of SCs.

#### f-SPION-mediated magnetomechanical stimulation upregulates the expression of key transcriptional regulators associated with the rSC

The activation of the SC repair program is orchestrated by the transcription factors c-Jun (Jun proto-oncogene, AP-1 transcription factor subunit) and signal transducer and activator of transcription 3 (STAT3), which are indispensable for the induction and maintenance of the repair phenotype [9, 15, 16, 53–56]. As shown in Fig. 4m–p, magnetomechanical stimulation significantly increased the expression of both c-Jun (Fig. 4m and n) and STAT3 (Fig. 4o and p) at both the mRNA and protein levels, relative to those in the untreated, f-SPION-only, and magnetic field-only control groups.

#### f-SPION-mediated magnetomechanical stimulation upregulates the expression of axon regeneration-related adhesion molecules in SCs

During axonal regeneration, adhesion molecules involved in axon growth and elongation—such as neural cell adhesion molecule (NCAM) and integrins—are markedly upregulated in rSCs, constituting a hallmark of their regenerative support function [57]. The expression levels of NCAM and integrin β1 were assessed across the experimental groups via RT-qPCR and western blot analyses. As shown in Fig. 4q–t, both NCAM (Fig. 4q and r) and integrin β1 (Fig. 4s and t) expression levels were significantly upregulated in the magnetically stimulated group compared with those in the normal control, f-SPIONs control, and magnetic field control groups.

Collectively, these in vitro findings demonstrate that SCs can sense piconewton (***pN***)-scale intermittent pulsed magnetomechanical stimulation via f-SPIONs and respond by reprogramming gene expression to adapt to the mechanical microenvironment, thereby adopting a repair-supportive phenotype.

### f-SPION-mediated magnetomechanical stimulation induces rSC reprogramming within the sciatic nerve

Building upon our in vitro findings that f-SPION-mediated magnetic actuation facilitates SC transition toward a repair phenotype, we performed mRNA transcriptome sequencing to assess the in vivo impact of magnetomechanical stimulation on SC gene expression in a rat sciatic nerve crush injury model. Studies have shown that at the distal stump of injured nerves, proliferating and migrating SCs constitute the predominant cell population [15, 58]. Therefore, distal nerve segments were collected from both the magnetic stimulation and crush injury control groups for mRNA sequencing to investigate how f-SPION-mediated magnetomechanical stimulation modulates gene expression in denervated SCs within injured sciatic nerves.

**Fig. 5:**
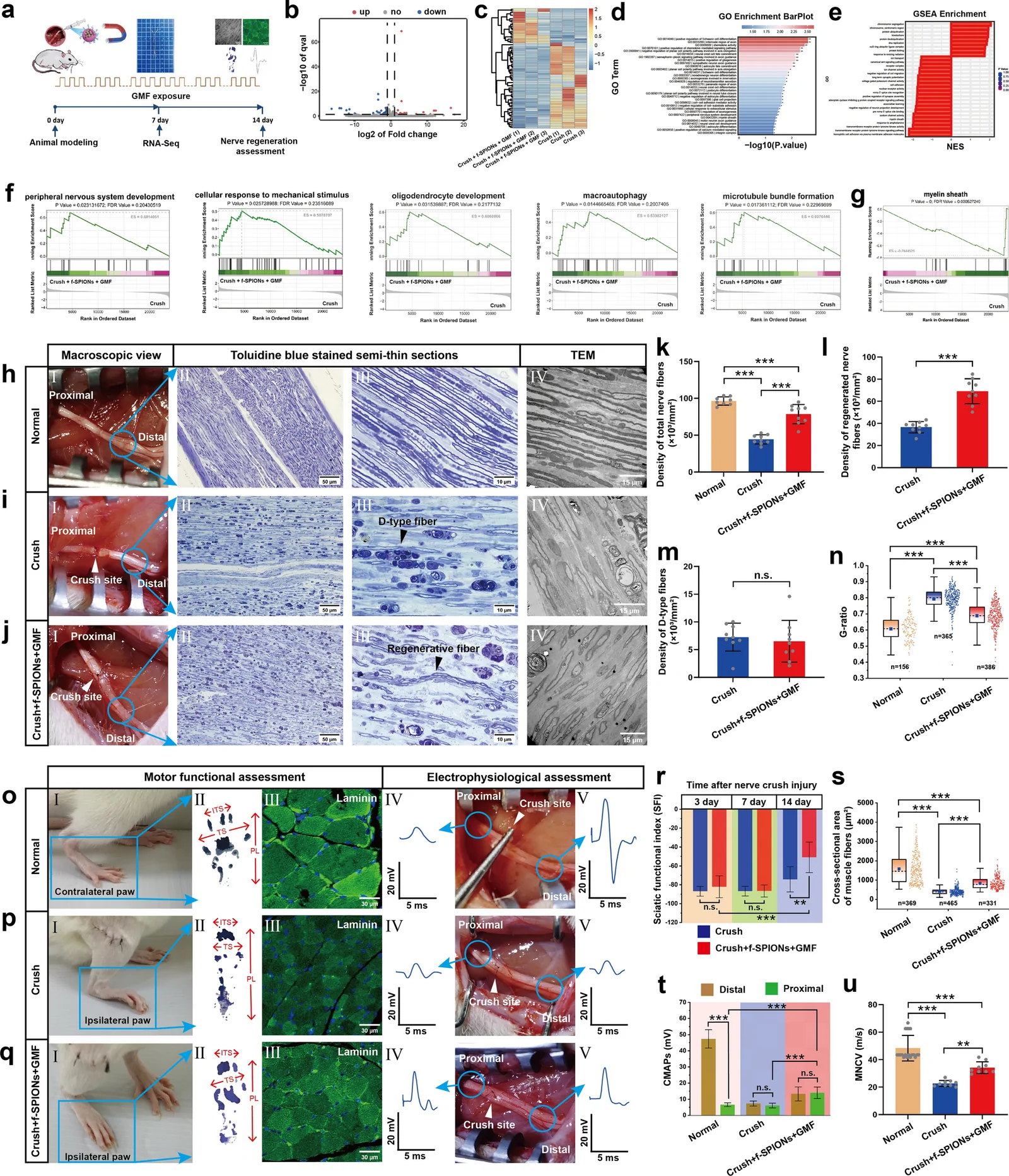
f-SPION-mediated magnetic actuation induces sciatic nerve SC reprogramming and promotes nerve regeneration in vivo. **a** Workflow of magnetic stimulation in a rat sciatic nerve crush injury model using f-SPIONs and a neodymium magnet-based gradient field. Distal sciatic nerve tissues were collected on day 7 for mRNA transcriptomic analysis, and regenerative outcomes were evaluated on day 14 post-injury. **b** Volcano plot showing the global distribution of DEGs between the magnetic stimulation group and the crush injury control group. **c** Heatmap of hierarchical clustering showing DEG expression trends between the magnetic stimulation group and the crush injury control group. **d** GO enrichment analysis revealed biological processes and functions associated with magnetic stimulation-induced DEGs. **e** GSEA further highlighted significantly enriched GO terms regulated by magnetic force stimulation. **f** GSEA identified elevated expression of gene sets associated with peripheral nervous system development, mechanotransduction, cAMP-mediated signaling, and autophagy in the magnetic stimulation group. **g** The gene sets related to myelination were significantly downregulated. **h** Gross and ultrastructural images of normal rat sciatic nerves. **i** Injury control group: (**i**(**I**)) gross view of the sciatic nerve immediately after crush injury; (**i**(**II–III**)) semi-thin sections and (**i**(**IV**)) TEM images of nerve regeneration at 14 days post-injury. **j** Magnetic stimulation group: (**j**(**I**)) gross appearance of the sciatic nerve immediately after crush injury; (**j**(**II–III**)) semi-thin sections and (**j**(**IV**)) TEM images of nerve regeneration on day 14 post-stimulation. **k–n** Quantitative analysis of (**k**) total nerve fiber density, (**l**) newly regenerated nerve fiber density, (**m**) D-type fiber density, and (**n**) G-ratio across experimental groups on day 14. **o** Normal control group: representative images of (**o**(**I**)) hind paw appearance, (**o**(**II**)) footprint morphology, (**o**(**III**)) immunofluorescence staining of laminin in gastrocnemius muscle, and (**o**(**IV**)) proximal and (**o**(**V**)) distal CMAP waveforms immediately after sciatic nerve crush injury. **p** Representative images from the injury control group at 14 days post-modeling, including (**p**(**I**)) hind paw appearance, (**p**(**II**)) footprint morphology, (**p**(**III**)) immunofluorescence staining of laminin in the gastrocnemius muscle, and CMAP waveforms at the (**p**(**IV**)) proximal and (**p**(**V**)) distal sites of the sciatic nerve crush. **q** Representative images from the magnetic stimulation group at 14 days after modeling and magnetic stimulation, including (**q**(**I**)) hind paw appearance, (**q**(**II**)) footprint pattern, (**q**(**III**)) immunofluorescence staining of laminin in the gastrocnemius muscle, and CMAP waveforms at the (**q**(**IV**)) proximal and (**q**(**V**)) distal sites of the sciatic nerve crush. **r–u** Quantitative assessment of functional and structural recovery, including (**r**) SFI, (**s**) cross-sectional area of gastrocnemius muscle fibers, (**t**) CMAP amplitude, and (**u**) MNCV across experimental groups on day 14 post-injury. The data are representative of three independent replicates. Each replicate experiment included 3 rats per group. “n” indicates the total number of nerve fibers or muscle fibers measured. Statistical significance: n.s., not significant; * *p* < 0.05; ** *p* < 0.01; *** *p* < 0.001. PL, print length; TS, toe spread; ITS, intermediary toe spread; CMAPs: compound muscle action potentials; MNCV: motor nerve conduction velocity

A schematic diagram depicting the in vivo magnetic stimulation process, in which f-SPIONs interact with a GMF emitted by neodymium magnets, is presented in Fig. 5a. As shown in the volcano plot (Fig. 5b), 76 DEGs were identified between the magnetic stimulation and crush injury control groups, comprising 22 upregulated and 54 downregulated genes. Hierarchical clustering heatmaps revealed distinct group-specific gene expression patterns (Fig. 5c), further confirming that f-SPION-mediated magnetomechanical stimulation elicited significant transcriptomic remodeling in the SCs of the sciatic nerve. GO enrichment analysis was conducted to associate DEGs with specific biological processes, thereby elucidating the functional implications of magnetic stimulation. As shown in Fig. 5d, the upregulated genes were significantly enriched in GO terms associated with SC dedifferentiation, migration, and peripheral nerve regeneration. Conversely, the downregulated genes were enriched primarily in GO terms related to SC differentiation and myelination. To further elucidate functional changes, GSEA was subsequently performed (Fig. 5e). As shown in Fig. 5f, GSEA revealed significant enrichment of gene sets related to peripheral nervous system development, cellular responses to mechanical stimulus, cAMP-mediated signaling, and autophagy in the magnetically stimulated group compared with the crush injury control group. In contrast, gene sets associated with myelination were significantly downregulated in the magnetic stimulation group (Fig. 5g).

Together, these findings indicate that in a rat sciatic nerve crush model, f-SPION-mediated micronewton (***μN***)-scale magnetic stimulation effectively reprograms SCs at the distal injury site toward a repair phenotype.

### f-SPION-mediated magnetomechanical stimulation promotes sciatic nerve regeneration and functional recovery following injury

After validating the in vivo reprogramming of SCs toward a repair phenotype induced by f-SPION-mediated magnetomechanical stimulation, we investigated its effects on nerve regeneration and functional recovery in a rat sciatic nerve injury model.

#### Morphological assessment of sciatic nerve regeneration

Fig. 5h presents the gross and microscopic morphology of an uninjured normal rat sciatic nerve. Following sciatic nerve crush injury, all the samples showed localized flattening at the lesion site, but preserved epineurial integrity and continuity (Fig. 5i(Ⅰ) and j(Ⅰ)). The ipsilateral hindlimb exhibited complete flaccid paralysis after injury. All the animals survived without signs of infection or autotomy.

At 14 days post-injury, sciatic nerves were harvested for morphological analysis. Macroscopic, semi-thin section optical microscopy and ultrathin section TEM images of the samples from the magnetic stimulation and crush injury control groups are shown in Fig. 5i(Ⅱ–Ⅳ) and j(Ⅱ–Ⅳ). In the crush injury control group, numerous demyelinated degenerating fibers (D-type) and characteristic features of Wallerian degeneration were still evident in longitudinal sections (Fig. 5i(Ⅲ)). In contrast, the magnetic stimulation group exhibited prominent axonal regeneration, with newly formed fibers demonstrating reduced diameters and thinner myelin relative to intact nerves (Fig. 5j(Ⅲ)). At 14 days post-sciatic nerve crush injury, the total nerve fiber density (78.50 ± 13.04 × 10^3^/μm^2^) (Fig. 5k) and newly regenerated nerve fiber density (69.01 ± 11.35 × 10^3^/μm^2^) (Fig. 5l) were significantly greater in the magnetic stimulation group than in the injury control group (44.33 ± 6.26 × 10^3^/μm^2^ and 36.60 ± 5.10 × 10^3^/μm^2^). In contrast, the density of D-type fibers in the magnetic stimulation group (6.51 ± 3.76×10^3^/μm^2^) was slightly lower than that in the injury control group (7.26 ± 2.52×10^3^/μm^2^), but the difference was not statistically significant (*p* > 0.05) (Fig. 5m). These findings suggest that f-SPION-mediated magnetic actuation accelerates Wallerian degeneration, promotes efficient clearance of myelin debris and axonal remnants, and significantly enhances the number and elongation of regenerating axons.

To further evaluate the effect of magnetic force-driven stimulation on the quality of regenerated nerve fibers, we performed TEM to examine the ultrastructures of nerve fibers across different experimental groups (Fig. 5i(Ⅳ) and j(Ⅳ)). The G-ratio, an established indicator of remyelination in regenerated fibers, was significantly lower in the magnetic stimulation group (0.69 [0.65–0.74]) than those in the injury control group (0.80 [0.76–0.83]) (Fig. 5n). These findings suggest that magnetic stimulation promotes improved myelination of regenerating axons.

#### Functional evaluation of sciatic nerve regeneration

After confirming the microstructural benefits of magnetic stimulation, we further assessed its impact on the functional recovery of the sciatic nerve in vivo.

The SFI serves as a quantitative measure of hindlimb muscle function—including the gastrocnemius, flexor digitorum longus, and tibialis posterior—innervated by the sciatic nerve (Fig. 5o(Ⅰ–Ⅱ)). Prior to injury, all the rats exhibited normal hindlimb function, with SFI scores of approximately 0. By day 7 after injury, complete sciatic nerve dysfunction resulted in pronounced hindlimb deficits in all groups, with SFI scores decreasing to approximately −100 (Fig. 5r). Movie S3 and Movie S4 show the gait videos for SFI assessment in rats at 3 and 7 days post-injury, respectively [see Additional File 4 and 5]. By day 14, the rats in the magnetic stimulation group exhibited substantial functional recovery of the hindlimb (Fig. 5q(Ⅰ–Ⅱ)) compared with those in the crush injury control group (Fig. 5p(Ⅰ–Ⅱ)), as indicated by significantly elevated SFI scores (−50.94 ± 16.21 in the “Crush + f-SPIONs + GMF” group vs. −74.18 ± 13.44 in the “Crush” group, *P* < 0.05) (Fig. 5r). Movie S5 presents the gait comparison video of rats between the “f-SPIONs + GMF” group and the “Crush” group at 14 days post-injury [see Additional File 6]. These findings indicate that magnetomechanical stimulation mediated by f-SPIONs significantly accelerated functional recovery of the sciatic nerve within 14 days following crush injury.

To further evaluate the effect of f-SPION-mediated magnetic stimulation on reinnervation of target muscles by the sciatic nerve, the ipsilateral gastrocnemius muscle was harvested from rats and analyzed by laminin immunofluorescence staining to assess muscle fiber morphology. Figure 5o(Ⅲ) displays the baseline morphology of the gastrocnemius muscle fibers innervated by an intact sciatic nerve. On day 14 post-injury, the magnetic stimulation group (Fig. 5q(Ⅲ)) displayed noticeably thicker muscle fibers compared to the injury control group (Fig. 5p(Ⅲ)). The cross-sectional area of gastrocnemius fibers in the magnetic stimulation group (775.87 [605.37–1027.51] μm^2^) was significantly larger than that in the injury control group (383.04 [298.37–484.08] μm^2^) (Fig. 5s). These morphological findings regarding target muscle reinnervation were consistent with improvements observed in SFI-based functional recovery.

#### Electrophysiological analysis of sciatic nerve regeneration

In addition to morphological and functional assessments, electrophysiological analysis was conducted to evaluate nerve regeneration in the rat model.

As shown in Fig. 5o(Ⅴ), the CMAP waveform recorded immediately distal to the sciatic nerve crush site represents a typical pattern of normal sciatic nerve conduction. In contrast, the proximal CMAP amplitude (Fig. 5o(Ⅳ)) recorded immediately after injury was markedly reduced compared to the distal site, indicating substantial disruption of axonal continuity at the crush site and confirming the success of the injury model. During the first week post-injury, no effective CMAP signals were elicited in any experimental group. However, by day 14 after nerve crush, the magnetic stimulation group exhibited significant recovery of electromyographic signals both proximal and distal to the injury site (Fig. 5q(Ⅳ–Ⅴ)), in contrast to the injury control group (Fig. 5p(Ⅳ–Ⅴ)). The magnetic stimulation group showed significantly higher CMAP amplitudes (13.28 ± 4.32 mV) (Fig. 5t) and MNCV values (34.17 ± 4.24 m/s) (Fig. 5u) compared to the injury control group (7.34 ± 1.54 mV and 22.68 ± 2.18 m/s, respectively).

These results indicate that magnetic stimulation significantly accelerates the electrophysiological recovery of the sciatic nerve within 2 weeks post-injury.

### f-SPION-mediated magnetomechanical stimulation activates actin cytoskeleton dynamics in SCs

Following confirmation of the regulatory effects of f-SPION-mediated magnetic stimulation on SC repair phenotype induction and peripheral nerve regeneration, we further investigated the underlying mechanisms of mechanotransduction.

**Fig. 6:**
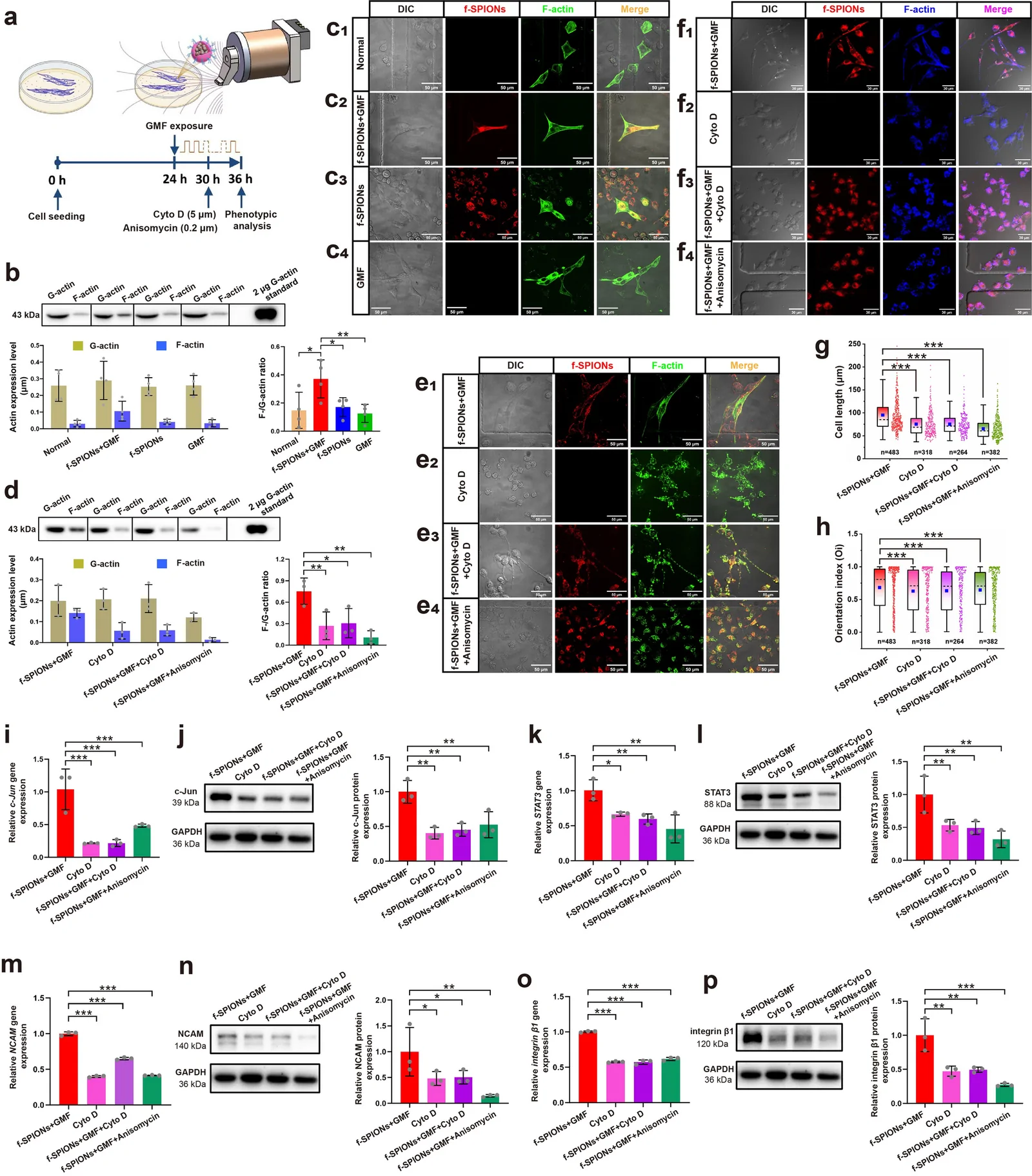
f-SPION-mediated magnetic actuation regulates SCs into a repair phenotype via activation of actin cytoskeletal dynamics. **a** Experimental workflow for investigating how magnetic force–dependent actin cytoskeletal dynamics regulate the SC repair phenotype. **b** Western blot quantification of F-actin and G-actin expression levels and F/G-actin ratios in SCs from normal control, magnetic stimulation, f-SPION control, and magnetic field control groups. **c** Live-cell actin staining revealed changes in F-actin cytoskeletal morphology under different conditions: (**c**_**1**_) normal control, (**c**_**2**_) magnetic stimulation, (**c**_**3**_) f-SPIONs only, and (**c**_**4**_) magnetic field only. **d** Western blot quantification of F-actin and G-actin expression levels and F/G-actin ratios in SCs from the “f-SPIONs + GMF”, “Cyto D”, “f-SPIONs + GMF + Cyto D”, and “f-SPIONs + GMF + Anisomycin” groups. **e** Live-cell actin staining visualized cytoskeletal dynamics under (**e**_**1**_) magnetic stimulation, (**e**_**2**_) Cyto D, (**e**_**3**_) magnetic stimulation + Cyto D, and (**e**_**4**_) magnetic stimulation + Anisomycin treatments. **f** Immunofluorescence imaging of SCs showed structural remodeling and morphological polarization in (**f**_**1**_) “f-SPIONs + GMF”, (**f**_**2**_) “Cyto D”, (**f**_**3**_) “f-SPIONs + GMF + Cyto D”, and (**f**_**4**_) “f-SPIONs + GMF + Anisomycin” groups. **g,h** Quantitative analysis of (**g**) cell length and (**h**) Oi under different treatments. **i,j** Expression levels of transcription factor c-Jun assessed by (**i**) RT-qPCR and (**j**) Western blot. **k,l** Expression levels of STAT3 measured by (**k)** RT-qPCR and (**l)** Western blot. **m,n** NCAM expression analyzed by (**m**) RT-qPCR and (**n**) Western blot. **o,p** Expression of integrin β1 evaluated by (**o**) RT-qPCR and (**p**) Western blot. Each experiment was independently repeated three or four times. “n” indicates the total number of cells measured. Statistical significance: * *p* < 0.05; ** *p* < 0.01; *** *p* < 0.001

Actin, a core structural component of cytoskeletal microfilaments, functions as a dynamic and mechanosensitive element essential for cellular force sensing and mechanical load distribution [59]. Within eukaryotic cells, actin exists in two primary forms: monomeric globular actin (G-actin) and polymerized filamentous actin (F-actin). In highly motile and deformable cells, G-actin monomers are actively transported to the leading edge, where they polymerize into F-actin networks that drive membrane protrusions and facilitate cell migration [60]. In this section, we investigated how magnetic actuation by f-SPIONs activates actin cytoskeleton dynamics and how this mechanotransduction event contributes to the induction of SC repair phenotypes. The experimental workflow is outlined in Fig. 6a.

#### f-SPION-mediated magnetomechanical stimulation enhances actin cytoskeletal remodeling in SCs

Western blot analysis using specialized detection kits remains the most reliable and precise method for quantifying F-actin and G-actin levels in cell populations [61–64]. In this study, we utilized the G-actin: F-actin In Vivo Assay Kit to quantify and compare actin levels in RSC96 cells across multiple experimental groups. As shown in Fig. 6b, compared with the normal control group, the f-SPION-mediated magnetic actuation significantly upregulated F-actin expression (0.030 ± 0.019 μg in the control group vs. 0.105 ± 0.059 μg in the magnetic stimulation group, p < 0.05) and increased the F/G-actin ratio (0.149 ± 0.128 vs. 0.371 ± 0.135, p < 0.05). In contrast, neither f-SPION exposure alone (0.041 ± 0.015 μg, 0.172 ± 0.067) nor magnetic field exposure alone (0.033 ± 0.022 μg, 0.126 ± 0.064) induced significant changes in F-actin expression or the F/G-actin ratio. Similarly, no comparable increase in the F-/G-actin ratio was observed in the SPIONs-mediated magnetic stimulation group (Fig. S12), as seen in the f-SPIONs-mediated magnetic stimulation group. This suggests that the magnetically induced activation of actin cytoskeleton dynamics depends on the targeted binding of f-SPIONs to F-actin.

To further confirm the effects of various interventions on actin cytoskeleton dynamics, live-cell actin staining and tracking followed by high-resolution imaging were conducted in RSC96 cells. As shown in Fig. 6c_2_, magnetic stimulation markedly promoted cellular elongation and neurite extension in RSC96 cells, with F-actin filaments aligning along the neurite axis to form organized stress fibers providing cytoskeletal support. In contrast, RSC96 cells in the normal control (Fig. 6c_1_), f-SPION control (Fig. 6c_3_), and magnetic field control groups (Fig. 6c_4_) maintained a spindle-like or oval morphology, with F-actin distributed as diffuse punctate structures lacking organized stress fiber assembly.

These results indicate that f-SPION-mediated magnetic stimulation significantly promotes F-actin polymerization, shifts the F/G-actin ratio toward filamentous structures, and activates cytoskeletal dynamics in RSC96 cells.

#### Reversal of magnetic force-induced actin cytoskeletal remodeling by pharmacological intervention

To further assess the role of magnetic force-induced actin cytoskeleton dynamics in SC reprogramming, we first verified the regulatory effects of pharmacological inhibitors (cytochalasin D and anisomycin) on actin cytoskeleton dynamics. Cytochalasin D (Cyto D) is a membrane-permeable mycotoxin that disrupts actin filament polymerization by inhibiting actin monomer assembly, whereas anisomycin inhibits protein synthesis by targeting ribosomal peptidyl transferase activity.

RSC96 cells were treated with Cyto D (5 μM) for 45 min, after which the intracellular F/G-actin ratio was assessed via a G-actin: F-actin In Vivo Assay Kit and Western blotting. As depicted in Fig. 6d, compared with magnetic stimulation, Cyto D treatment markedly reduced F-actin expression and decreased the F/G-actin ratio. The expression level of F-actin was 0.056 ± 0.039 μg in the “Cyto D” group compared with 0.142 ± 0.022 μg in the “f-SPIONs+GMF” group (*p* < 0.05), and the F/G-actin ratio was 0.270 ± 0.193 in the “Cyto D” group versus 0.754 ± 0.184 in the “f-SPIONs+GMF” group (*p* < 0.05). Furthermore, cotreatment with magnetic stimulation and Cyto D significantly reversed the actin upregulation induced by f-SPION-mediated stimulation. The expression level of F-actin and the F/G-actin ratio in the “f-SPIONs+GMF+Cyto D” group were 0.059 ± 0.026 μg and 0.308 ± 0.204, respectively, both of which were significantly lower than those in the “f-SPIONs+GMF” group (*p* < 0.05). A comparable reversal effect was also observed in the “f-SPIONs+GMF+Anisomycin” group, in which 0.2 μM anisomycin treatment for 12 h suppressed the magnetic force-induced increase in actin expression (Fig. 6d). The expression level of F-actin and the F/G-actin ratio in the “f-SPIONs+GMF+Anisomycin” group were 0.013 ± 0.010 μg and 0.108 ± 0.093, respectively, both of which were significantly lower than those in the “f-SPIONs+GMF” group (*p* < 0.05). As shown in Fig. 6e_2_, Cyto D treatment disrupted the integrity of F-actin stress fibers, resulting in fragmented and punctate structures scattered throughout the cytoplasm. Similarly, cotreatment with magnetic stimulation and either Cyto D (Fig. 6e_3_) or anisomycin (Fig. 6e_4_) abolished the magnetic force-induced formation of actin stress fibers.

Collectively, these findings confirm that both Cyto D and anisomycin effectively counteract the magnetic force-induced activation of actin cytoskeletal dynamics.

#### f-SPION-mediated magnetomechanical stimulation initiates rSC reprogramming via the activation of actin cytoskeleton dynamics

After confirming the reversal effects of Cyto D and anisomycin on magnetic force-induced actin cytoskeleton activation, we further investigated their impact on magnetic force-induced SC repair phenotype reprogramming.

First, we employed immunofluorescence imaging to assess the morphology of RSC96 cells—specifically cell length and Oi—in the “f-SPIONs+GMF”, “Cyto D”, “f-SPIONs+GMF+Cyto D”, and “f-SPIONs+GMF+Anisomycin” groups (Fig. 6f). The results showed that, compared to the magnetic stimulation group, treatment with Cyto D or anisomycin significantly attenuated the magnetic force-induced elongation of RSC96 cells (85.15 [70.71–111.98] μm in the “f-SPIONs+GMF” group vs. 71.22 [59.58–88.29] μm in the “f-SPIONs+GMF+Cyto D” group vs. 60.67 [48.53–77.02] μm in the “f-SPIONs+GMF+Anisomycin” group) (Fig. 6g), and reduced their Oi (0.807 [0.410–0.964] in the “f-SPIONs+GMF” group vs. 0.703 [0.336–0.923] in the “f-SPIONs+GMF+Cyto D” group vs. 0.705 [0.426–0.913] in the “f-SPIONs+GMF+Anisomycin” group) (Fig. 6h). These findings indicate that Cyto D and anisomycin suppress the magnetic force-dependent structural remodeling and morphological polarization characteristic of the SC repair phenotype by counteracting the activation of actin cytoskeletal expression and dynamics.

RT-qPCR and Western blot analysis were subsequently performed to examine how the actin cytoskeletal status influences the expression of key regulatory molecules involved in SC repair phenotypes. As shown in Fig. 6i–l, both Cyto D and anisomycin treatments reversed the magnetic force-induced upregulation of key repair-associated transcription factors, significantly reducing the expression levels of c-Jun (Fig. 6i and j) and STAT3 (Fig. 6k and l), compared with those in the magnetic stimulation group. Moreover, the expression of adhesion molecules critical for axonal regeneration—NCAM (Fig. 6m and n) and integrin β1 (Fig. 6o and p)—was similarly downregulated following Cyto D and anisomycin treatment.

Collectively, these results demonstrate that inhibition of actin cytoskeletal dynamics effectively abrogates the magnetic force-induced reprogramming of the rSC. Accordingly, we propose that in the magnetic stimulation group, f-SPION-mediated magnetomechanical stimulation initiates rSC reprogramming through the modulation of actin cytoskeletal organization and dynamics.

### f-SPION-mediated magnetomechanical stimulation upregulates the expression of mechanosensitive Piezo1 ion channels in SCs

Piezo1 is a mechanosensitive ion channel that functions as a cellular mechanoreceptor and is capable of detecting and transducing mechanical stimuli. Recent structural studies have shown that Piezo channels sense changes in membrane curvature and tension, thereby activating mechanically gated responses and modulating channel functional states [65].

#### Mechanosensitive Piezo1 ion channels are highly and specifically expressed in the SCs of peripheral nerves

**Fig. 7:**
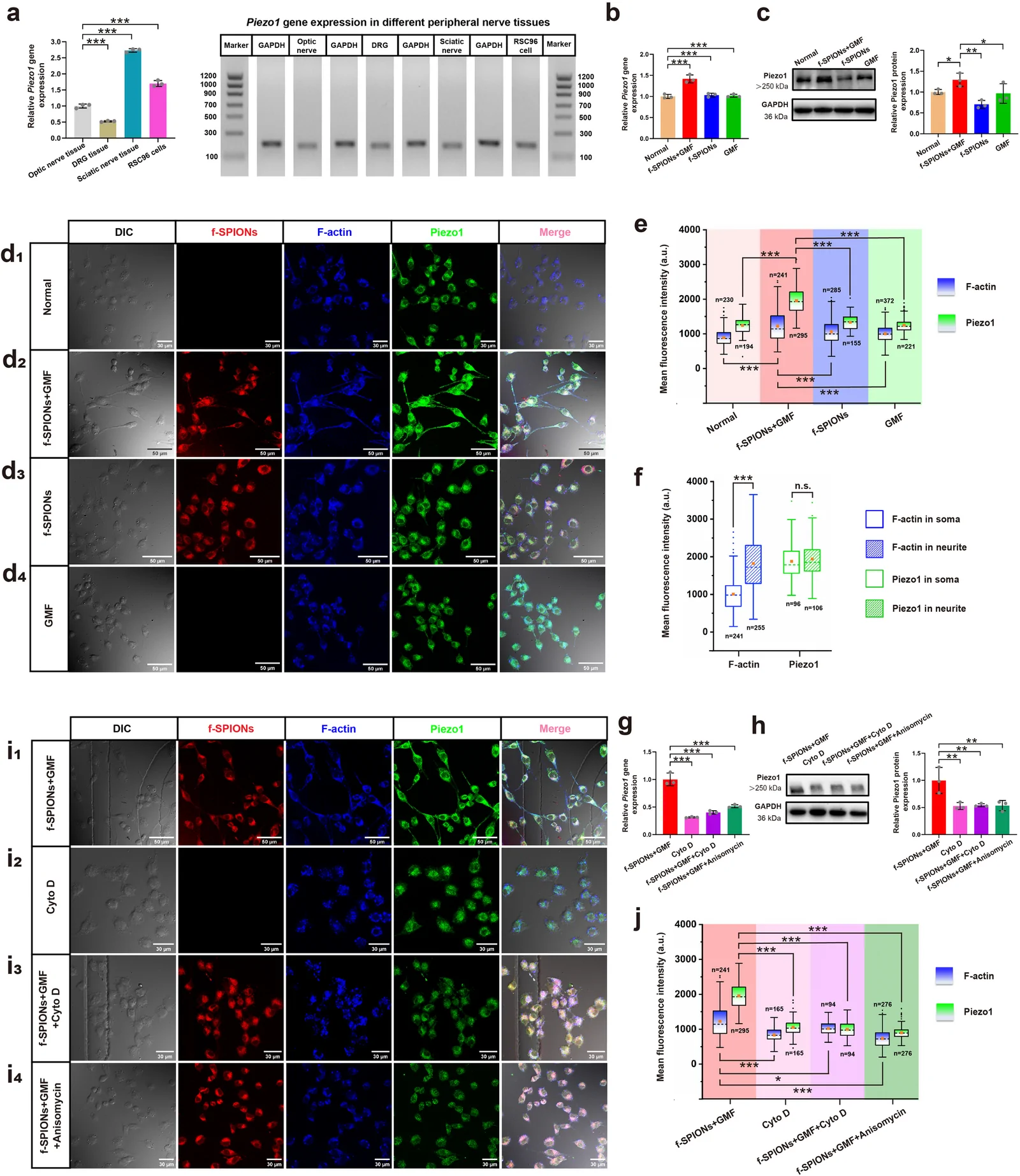
f-SPIONs-mediated magnetic actuation upregulates Piezo1 expression via activation of actin cytoskeleton dynamics. **a** RT-qPCR analysis of Piezo1 mRNA expression levels across different peripheral nerve tissues. **b,c** (**b**) RT-qPCR and (**c**) Western blot analyses of Piezo1 expression in Schwann cells from normal control, magnetic stimulation, f-SPIONs control, and magnetic field control groups. **d** Immunofluorescence imaging of F-actin and Piezo1 expression and subcellular distribution across different treatment groups: (**d**_**1**_) normal control, (**d**_**2**_) magnetic stimulation, (**d**_**3**_) f-SPIONs only, and (**d**_**4**_) magnetic field only groups. **e** Quantitative analysis of fluorescence intensity of F-actin and Piezo1 across different treatment groups. **f** Subcellular fluorescence distribution of F-actin and Piezo1 within soma and neurite compartments under magnetic stimulation. **g,h** Piezo1 expression levels measured by (**g**) RT-qPCR and (**h**) Western blot in magnetic stimulation, Cyto D, magnetic + Cyto D, and magnetic + Anisomycin groups. **i** Immunofluorescence imaging of F-actin and Piezo1 expression in SCs treated with (**i**_**1**_) magnetic stimulation, (**i**_**2**_) Cyto D, (**i**_**3**_) magnetic stimulation + Cyto D, and (**i**_**4**_) magnetic stimulation + Anisomycin. **j** Quantitative comparison of F-actin and Piezo1 fluorescence intensity across different treatment groups. Data are representative of three independent replicates. “n” indicates the total number of cells measured. Statistical significance: * *p* < 0.05; ** *p* < 0.01; *** *p* < 0.001

We first assessed Piezo1 mRNA expression across various tissues and cell types of the peripheral nervous system via RT-qPCR. The tested samples included dorsal root ganglia (DRG), which are composed of primary sensory neurons; sciatic nerve tissue, a distal segment rich in SCs; and RSC96 cells, a rat-derived SC line of the peripheral nervous system. The optic nerve, which is known for expressing various mechanosensitive ion channels, served as a positive control [66, 67]. As shown in Fig. 7a, Piezo1 mRNA displayed a distinct tissue-specific expression pattern, with significantly higher levels in the sciatic nerve and RSC96 cells than in the optic nerve and DRG. These findings suggest that Piezo1 channels may serve as primary molecular mechanosensors in SCs within the peripheral nervous system.

#### f-SPIONs-mediated magnetomechanical stimulation regulates Piezo1 ion channel expression

To evaluate the responsiveness of Piezo1 to magnetomechanical cues, we measured its expression levels via RT-qPCR and Western blotting across four groups: the untreated normal control, magnetic stimulation, f-SPIONs control, and magnetic field control groups. As shown in Fig. 7b and c, f-SPION-mediated magnetomechanical stimulation significantly increased Piezo1 expression at both the mRNA and protein levels in the magnetic stimulation group.

Furthermore, we employed immunofluorescence imaging to investigate the expression and subcellular distribution of F-actin and Piezo1 under different intervention conditions (Fig. 7d). Quantitative analysis of fluorescence intensity revealed that both F-actin and Piezo1 signals were significantly elevated in the magnetic stimulation group compared with those in the control groups (Fig. 7e), suggesting a potential correlation between magnetic force-induced Piezo1 upregulation and the activation of actin cytoskeletal dynamics.

These findings suggest that Piezo1, a mechanosensitive ion channel, is highly responsive to modulation by f-SPION-mediated magnetomechanical stimulation.

#### f-SPION-mediated magnetomechanical stimulation regulates Piezo1 ion channel expression via the activation of cytoskeletal dynamics

In the preceding section, we demonstrated that f-SPION-mediated magnetomechanical stimulation activated actin cytoskeletal dynamics. Thus, we hypothesized that cytoskeletal activation functions upstream in mediating the magnetic stimulation-induced upregulation of Piezo1 expression.

To test this hypothesis, we assessed Piezo1 expression via RT-qPCR and Western blotting across four groups—magnetic stimulation, Cyto D treatment, magnetic stimulation combined with Cyto D treatment, and magnetic stimulation combined with anisomycin treatment—to evaluate the effect of cytoskeletal inhibition on Piezo1 regulation. As shown in Fig. 7g and h, treatment with either Cyto D or anisomycin significantly attenuated the magnetic force-induced upregulation of Piezo1, confirming that its expression is dependent on activated cytoskeletal dynamics.

To further validate the RT-qPCR and Western blot results, we performed immunofluorescence imaging to examine the expression and distribution of F-actin and Piezo1 across different experimental groups (Fig. 7i). Quantitative analysis of the immunofluorescence signals revealed that treatment with Cyto D and anisomycin not only suppressed magnetic force-induced cytoskeletal remodeling, but also significantly downregulated the expression of the Piezo1 ion channel (Fig. 7j). These results confirm that the magnetic stimulation-induced upregulation of Piezo1 is regulated upstream by the dynamic state of the actin cytoskeleton.

Collectively, these results indicate that f-SPION-mediated magnetomechanical stimulation regulates the downstream Piezo1 ion channel in a gated manner by activating the dynamic state of the actin cytoskeleton.

### f-SPION-mediated magnetomechanical stimulation activates SC Ca^2+^ dynamics via Piezo1

#### ***f-SPION-mediated magnetomechanical stimulation activates SC Ca***^***2+***^*** dynamics***

**Fig. 8:**
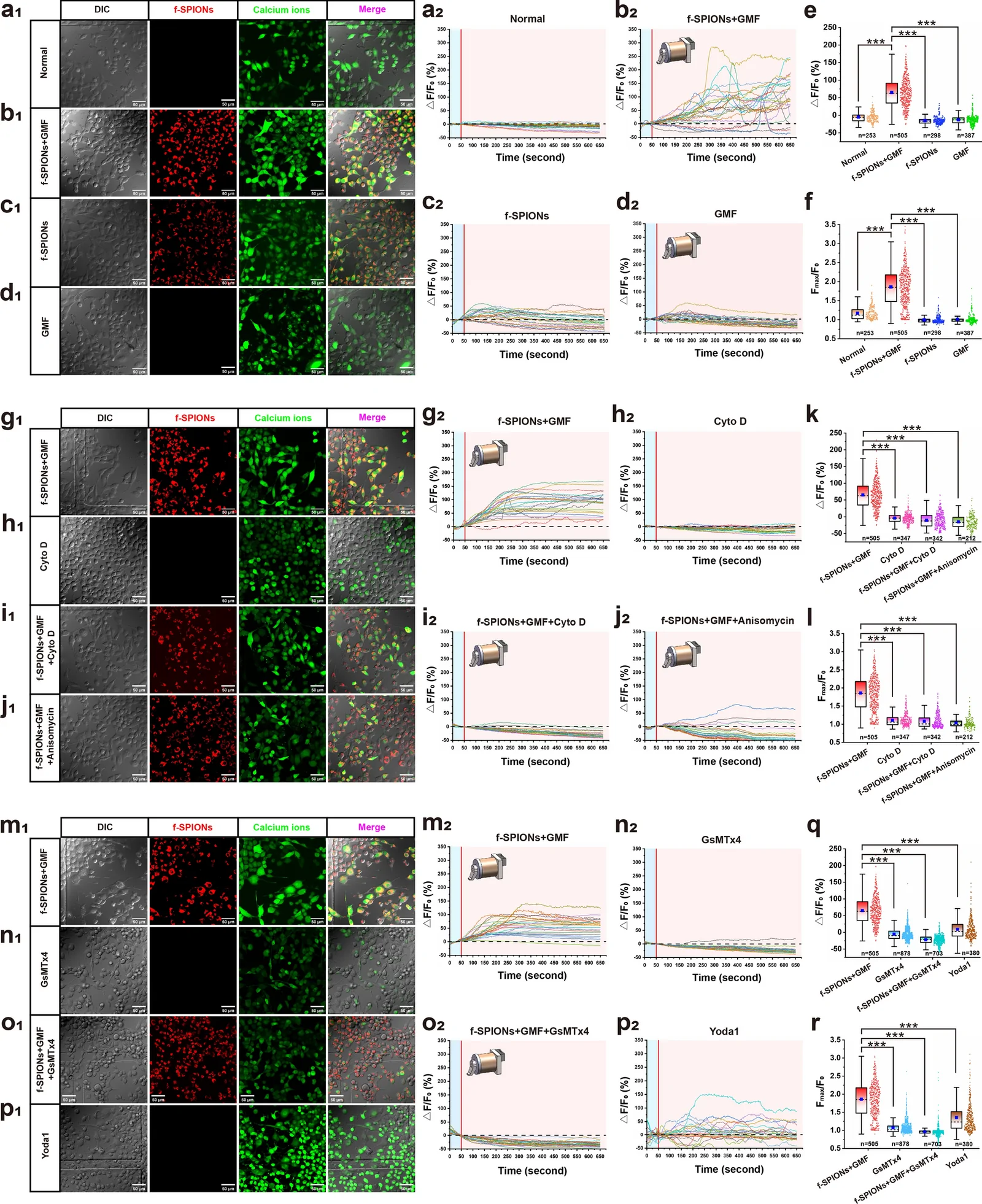
f-SPION-mediated magnetic actuation activates Ca^2^⁺ dynamics in Schwann cells via Piezo1 ion channel signaling. **a**_**1**_-**d**_**1**_ Representative Ca^2^⁺ fluorescence images of SCs in the (**a**_**1**_) normal control, (**b**_**1**_) magnetic stimulation, (**c1**) f-SPIONs control, and (**d**_**1**_) magnetic field control groups. **a**_**2**_**-d**_**2**_ Corresponding Ca^2^⁺ signal trajectories from the four groups, showing temporal changes in intracellular calcium. **e,f** Quantification of (**e**) relative fluorescence intensity change (ΔF/F₀) and (**f**) peak fluorescence intensity (F_max_/F_0_) of Ca^2^⁺ signaling in different treatment groups. **g**_**1**_**-j**_**1**_ Representative Ca^2^⁺ fluorescence images in (**g**_**1**_) magnetic stimulation, (**h**_**1**_) Cyto D, (**i**_**1**_) magnetic stimulation + Cyto D, and **(j**_**1**_) magnetic stimulation + Anisomycin groups. **g**_**2**_**-j**_**2**_ Corresponding Ca^2^⁺ signal trajectories showing real-time intracellular Ca^2^⁺ fluctuations under the above treatments. **k,l** Quantification of (**k**) ΔF/F₀ and (**l**) F_max_/F_0_ in different cytoskeleton intervention groups. **m**_**1**_**-p**_**1**_ Representative Ca^2^⁺ fluorescence imaging of SCs in (**m**_**1**_) magnetic stimulation, (**n**_**1**_) GsMTx4 (Piezo1 inhibitor), (**o**_**1**_) magnetic stimulation + GsMTx4, and (**p**_**1**_) Yoda1 (Piezo1 agonist) groups. **m**_**2**_**-p**_**2**_ Corresponding Ca^2^⁺ signal trajectories under the above intervention groups. **q,r** Quantification of (**q**) ΔF/F₀ and (**r**) F_max_/F_0_ across Piezo1-specific intervention groups. Each experiment was independently repeated three times. “n” indicates the total number of cells measured. Baseline fluorescence intensity (F₀) was normalized to the first 50 s of imaging. GMF exposure was initiated after 50 s, and Ca^2^⁺ time-lapse imaging continued for a total of 650 s. Statistical significance: * *p* < 0.05; ** *p* < 0.01; *** *p* < 0.001

To investigate the effect of f-SPION-mediated magnetic actuation on Ca^2^⁺ dynamics, we employed live-cell functional calcium imaging to monitor real-time changes in Ca^2^⁺ signaling across different experimental groups. As shown in Fig. 8a–d, representative Ca^2^⁺ fluorescence images and corresponding Ca^2^⁺ signal trajectories were recorded for the normal control (Fig. 8a_1_ and a_2_), magnetic stimulation (Fig. 8b_1_ and b_2_), f-SPIONs control (Fig. 8c_1_and c_2_), and magnetic field control groups (Fig. 8d_1_ and d_2_). Under f-SPION-mediated magnetic stimulation, RSC96 cells exhibited significantly elevated Ca^2^⁺ fluorescence signals (Fig. 8b_1_) and pronounced Ca^2^⁺ transients (Fig. 8b_2_). Quantitative analysis revealed that both the change in Ca^2^⁺ fluorescence intensity (ΔF/F₀) (62.96 [35.07–91.67]) and peak amplitude Ca^2^⁺ fluorescence intensity (F_max_/F₀) (1.85 [1.48–2.18]) in the magnetic stimulation group were markedly greater than those in the normal control (−5.74 [−15.66–1.20] and 1.12 [1.03–1.26]), f-SPIONs control (−16.70 [−22.02 to −12.00] and 0.97 [0.94–1.01]), and magnetic field control groups (−12.90 [−21.24 to −6.97] and 0.99 [0.96–1.02]) (Fig. 8e and f). Movie S6 presents the dynamic monitoring of Ca^2+^ kinetics in the experimental groups mentioned above [see Additional File 7].

#### ***f-SPION-mediated magnetomechanical stimulation activates Ca***^***2+***^*** signaling by modulating cytoskeletal dynamics***

To elucidate the mechanism underlying Ca^2^⁺ activation by magnetic stimulation, we investigated the regulatory interplay between cytoskeletal dynamics and Ca^2^⁺ signaling. We analyzed Ca^2^⁺ signaling responses across four conditions: magnetic stimulation (Fig. 8g_1_ and g_2_), Cyto D treatment (Fig. 8h_1_ and h_2_), magnetic stimulation with Cyto D (Fig. 8i_1_ and i_2_), and magnetic stimulation with anisomycin (Fig. 8j_1_ and j_2_). Compared with the robust Ca^2^⁺ activation observed under magnetic stimulation, the inhibition of actin cytoskeletal dynamics via Cyto D or anisomycin markedly attenuated the intracellular Ca^2^⁺ fluorescence intensity and suppressed Ca^2^⁺ transients. The ΔF/F₀ values in the “f-SPIONs+GMF+Cyto D” and “f-SPIONs+GMF+Anisomycin” groups were −12.36 [−27.24–4.21] and −16.49 [−29.08 to −1.23], respectively, which were both significantly lower than the value of 62.96 [35.07–91.67] in the “f-SPIONs+GMF” group (p < 0.05) (Fig. 8k). F_max_/F_₀_ also exhibited a consistent intergroup trend with ΔF/F₀ (Fig. 8l). Movie S7 presents the dynamic monitoring of Ca^2+^ kinetics in the experimental groups mentioned above [see Additional File 8].

These findings confirm that f-SPION-mediated magnetomechanical stimulation activates intracellular Ca^2^⁺ dynamics in SCs through cytoskeletal modulation.

#### ***f-SPION-mediated magnetomechanical stimulation triggers Ca***^***2***^***⁺ signaling via cytoskeletal dynamics-gated Piezo1 ion channels***

In previous studies, we demonstrated that f-SPION-mediated magnetomechanical stimulation enables mechanical gating of Piezo1 ion channels through the modulation of F-actin cytoskeleton dynamics. We subsequently investigated the regulatory role of Piezo1 channels activated by cytoskeletal dynamics in mediating intracellular Ca^2^⁺ responses. To selectively manipulate Piezo1 channel activity, we employed the pharmacological agents Yoda1 and GsMTx4. Yoda1 is a selective agonist of mechanosensitive Piezo1 channels that promotes channel opening and Ca^2^⁺ influx. RSC96 cells treated with Yoda1 (10 μM) served as the positive control group (Fig. 8p_1_ and p_2_). Live-cell calcium imaging revealed that Yoda1-treated cells presented comparable increases in intracellular Ca^2^⁺ fluorescence intensity and transient amplitude to those observed in the magnetic stimulation group (Fig. 8q and r). These findings indicate that magnetic stimulation mediated by f-SPIONs activates the Piezo1 ion channel with efficacy comparable to that of Yoda1.

GsMTx4, a peptide toxin derived from spider venom, functions as an inhibitor of mechanosensitive Piezo channels. The cells treated with GsMTx4 (10 μM) served as the negative control group (Fig. 8n_1_ and n_2_). Live-cell calcium imaging demonstrated that GsMTx4 treatment markedly suppressed intracellular Ca^2^⁺ fluorescence signals (Fig. 8q and r). In the magnetic stimulation combined with GsMTx4 group, GsMTx4 treatment significantly diminished Ca^2^⁺ fluorescence in SCs (Fig. 8o_1_ and o_2_), effectively reversing magnetically induced Ca^2^⁺ activation (Fig. 8q and r). These findings suggest that magnetically triggered Ca^2^⁺ dynamic activation is mediated through mechanical gating of the Piezo1 ion channel. Movie S8 presents the dynamic monitoring of Ca^2+^ kinetics in the experimental groups mentioned above [see Additional File 9].

Collectively, these findings demonstrate that the f-SPION-mediated magnetomechanical stimulation effect activates intracellular Ca^2^⁺ dynamics by regulating cytoskeletal dynamics, which subsequently leads to the mechanical gating of Piezo1 ion channels.

### Magnetomechanical stimulation triggered Ca^2+^ dynamics to induce rSC reprogramming

As key second messengers, transmembrane Ca^2^⁺ dynamics orchestrate diverse biological processes—such as gene transcription, mRNA translation, and post-translational modifications—and are critically involved in peripheral nerve injury responses and regeneration. We therefore hypothesized that the activation of Ca^2^⁺ dynamics induced by f-SPION-mediated magnetomechanical stimulation plays a pivotal role in initiating the transition of SCs toward a repair-supportive phenotype.

**Fig. 9:**
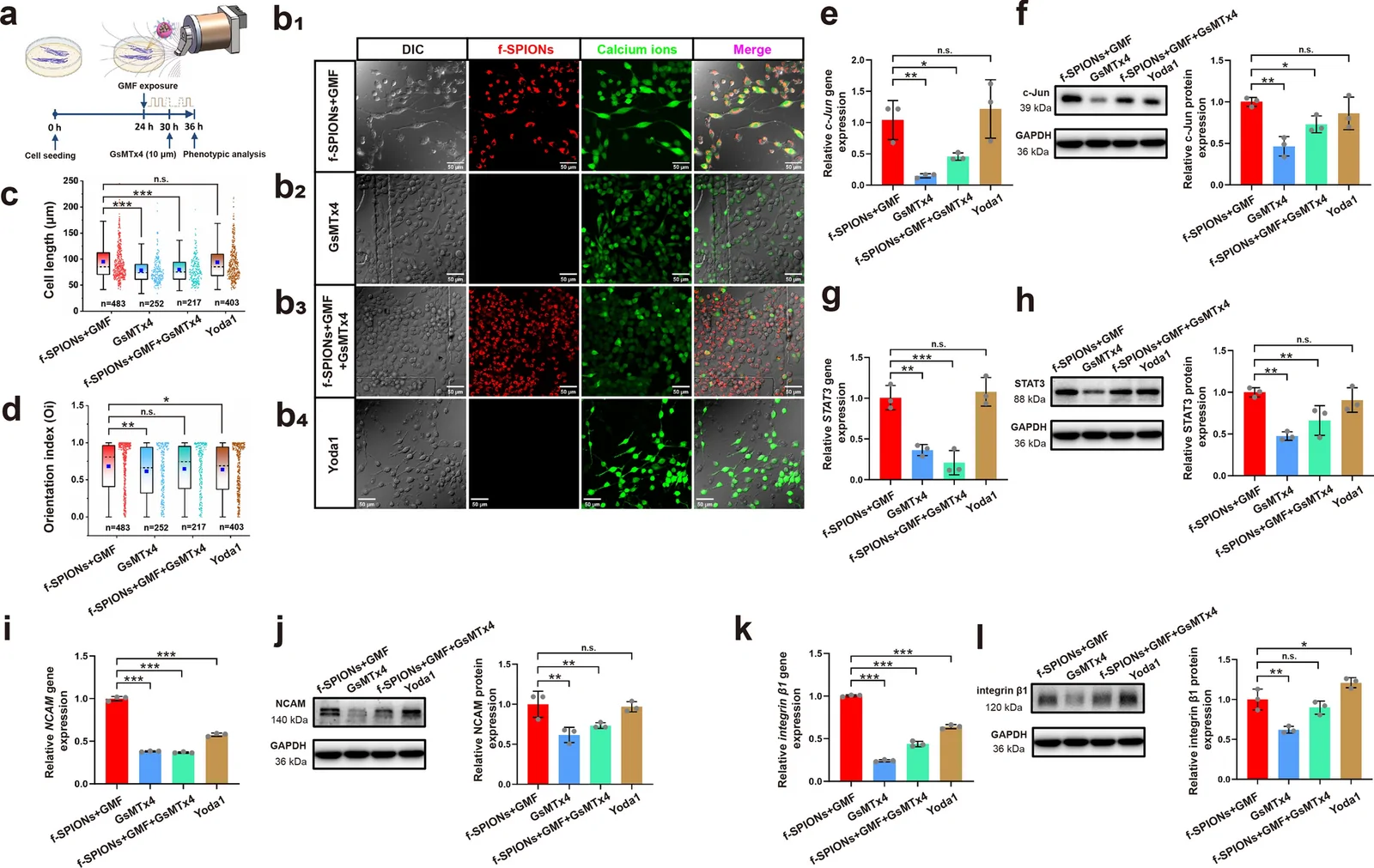
Magnetically triggered Ca^2^⁺ dynamics induce Schwann cell reprogramming toward a reparative phenotype. **a** Schematic diagram of the experimental workflow investigating the regulatory effect of magnetically triggered Ca^2^⁺ dynamics activation on the reparative phenotype of SCs. **b** Live-cell Ca^2^⁺ fluorescence imaging showing the effects of different treatments on SC structural remodeling and morphological polarization: (**b**_**1**_) magnetic stimulation, (**b**_**2**_) GsMTx4 (Piezo1 inhibitor), (**b**_**3**_) magnetic stimulation + GsMTx4, and (**b**_**4**_) Yoda1 (Piezo1 agonist). **c**, **d** Quantification of (**c**) cell length and (**d**) Oi across Piezo1-specific intervention groups. **e**, **f** Expression of the transcription factor c-Jun measured by (**e**) RT-qPCR and (**f**) Western blot. **g**, **h** STAT3 expression levels evaluated using (**g**) RT-qPCR and (**h**) Western blot. **i**, **j** NCAM expression assessed by (**i**) RT-qPCR and (**j**) Western blot. **k**, **l** Expression of integrin β1 determined via (**k**) RT-qPCR and (**l**) Western blot. Each experiment was independently repeated three times. “n” indicates the total number of cells measured. Statistical significance: n.s., not significant; * *p* < 0.05; ** *p* < 0.01; *** *p* < 0.001

Figure 9a illustrates the experimental workflow designed to investigate the effects of modulating Ca^2+^ dynamics on the repair phenotype of SCs. We first evaluated how Ca^2^⁺ dynamic activation affects structural remodeling and morphological polarization in SCs. Morphometric parameters, including cell length and orientation index (Oi), were quantified in RSC96 cells across four groups: magnetic stimulation (Fig. 9b_1_), GsMTx4 treatment (Fig. 9b_2_), magnetic stimulation combined with GsMTx4 (Fig. 9b_3_), and Yoda1 treatment (Fig. 9b_4_). The results demonstrated that the Yoda1 group exhibited a similar degree of morphological elongation in RSC96 cells as the magnetic stimulation group (85.09 [68.41–109.49] μm in the “Yoda 1” group vs. 85.15 [70.71–111.98] μm in the “f-SPIONs+GMF” group, *p* > 0.05) (Fig. 9c). However, notably, the magnetic stimulation group showed a higher Oi compared to the Yoda1 group (0.69 [0.37–0.94] in the “Yoda 1” group vs. 0.81 [0.41–0.96] in the “f-SPIONs+GMF” group) (Fig. 9d). In contrast, GsMTx4 effectively counteracted the magnetic stimulation-induced cellular structural remodeling (75.34 [61.42–94.12] μm in the “f-SPIONs+GMF+GsMTx4” group) (Fig. 9c) and directional polarization (0.75 [0.38–0.95] in the “f-SPIONs+GMF+GsMTx4” group) (Fig. 9d). These findings indicate that the activation of Ca^2^⁺ dynamics orchestrates cytoskeletal remodeling and directional polarization in rSCs.

We next assessed how Ca^2^⁺ dynamic activation influences the expression of key regulatory molecules involved in SC repair phenotypes via RT-qPCR and Western blotting. Both the magnetic stimulation and Yoda1 treatment groups showed similar upregulation of the repair-associated transcription factors c-Jun (Fig. 9e and f) and STAT3 (Fig. 9g and h), along with increased expression of the axonal regeneration-promoting molecules NCAM (Fig. 9i and j) and integrin β1 (Fig. 9k and l).

In summary, these results demonstrate that f-SPION-mediated magnetomechanical stimulation promotes SC phenotypic reprogramming by sequentially activating actin cytoskeletal dynamics, opening Piezo1 ion channels, and triggering intracellular Ca^2^⁺ signaling.

## Discussion

### Advances in mechanobiology offer novel insights into the regulation of nerve regeneration

Mechanobiology is an emerging discipline that has advanced rapidly over the past two decades [30, 33, 68]. It primarily investigates the interactions between cells and their mechanical microenvironment, focusing on how cells sense and respond to mechanical stimuli, and elucidates the interplay between mechanical processes and biological responses in living systems [34–37, 69, 70]. Today, mechanobiology has become one of the most dynamic and promising fields in biomedical engineering. Recent advances have demonstrated that physical mechanical stimulation plays a critical role in the development and regeneration of neural tissues, offering novel perspectives for regenerative therapies following nerve injury [28, 29, 71].

A variety of techniques have been developed in neuroscience to apply microscale mechanical forces to tissues or cells, which can be broadly categorized as follows: (1) Application of mechanical forces via cell stretch devices. Shan et al. [72] employed a microtissue stretching system (Flexcell^®^ FX Tension System) to mechanically stretch cultured Schwann cells in vitro, resulting in the upregulation of glycolytic and oxidative metabolism, and promoting nerve regeneration through enhanced glia-axon metabolic coupling. (2) Modulation of the cellular mechanical microenvironment via culture substrates with adjustable stiffness or nanotopography. Lei et al. [73] cultured DRG neurons on polyacrylamide substrates of varying stiffness to explore the influence of extracellular mechanical signals on axonal regeneration. (3) Application of mechanical stress via optical tweezers (OTs). Falleroni et al. [28] applied localized vertical compressive forces of 5–50 piconewtons to hippocampal neurons by oscillating optical tweezers, which induced CAMKII and RhoA activation and resulted in growth cone morphological changes. (4) Application and measurement of mechanical stress via atomic force microscopy (AFM) or nanoindentation. Hu et al. [74] used AFM to characterize the mechanical properties of amyloid-β plaque-associated tissue in the brains of Alzheimer’s disease model mice and investigated how microglia sense the mechanical properties of plaque-containing tissues. (5) The application of mechanical stimulation via micropipette aspiration (MPA), which alters the geometry and tension of the cell membrane [75, 76].

While these technologies for applying microscale mechanical forces to tissues and cells have been widely adopted in mechanobiology research, they are fundamentally dependent on direct physical manipulation or culturing of cells in artificially engineered mechanical microenvironments. Therefore, the translation of these mechanical modulation techniques into clinical applications for nervous system disorders remains a major challenge, with significant barriers yet to be addressed.

### Advantages and biomedical potential of f-SPION-mediated magnetomechanical neuromodulation

In neuroscience, there has been a longstanding pursuit developing an effective and clinically translatable neuromodulation strategy for promoting nerve regeneration. An ideal mechanotransduction approach for neural regeneration or neuromodulation should be applicable within the in vivo neural microenvironment and capable of delivering remote, noninvasive stimulation with cell-type specificity and high spatiotemporal resolution. Magnetic nanoparticles (MNPs)-based magnetic neuromodulation has emerged as a promising and minimally invasive technology. This approach leverages the negligible magnetic susceptibility of biological tissues and the deep tissue penetrability of magnetic fields, allowing for remote, noninvasive transmission of magnetic forces to internal target organs and cellular structures in vivo [77–83]. Wheeler et al. [84] engineered a magnetically responsive cation channel by fusing the mechanosensitive TRPV4 ion channel with paramagnetic ferritin, enabling remote modulation of calcium signaling in striatal dopamine receptor 1-expressing neurons under an external magnetic field, ultimately influencing reward-related behaviors in mice. Falconieri et al. [85] cocultured iron oxide MNPs with mouse DRG neurons to magnetize both neuronal somata and axons. Under an external static magnetic field, MNP-mediated nanoscale tensile forces enhance microtubule stability, activate local axonal translation, and consequently promote axonal regeneration. Xia et al. [86] designed a magnetomechanical stimulation system utilizing iron oxide MNPs and external magnetic fields to apply mechanical stimulation to SCs. Subsequent miRNA sequencing of exosomes derived from these mechanically stimulated SCs revealed mechanosensitive upregulation of microRNA-23b-3p, which promoted nerve regeneration by downregulating the expression of its neuronal target gene, neuropilin-1. Semeano et al. [81] cultured mouse embryonic stem cells (mESCs) and human induced pluripotent stem cells (hiPSCs) on MNP-loaded polymer substrates, and applied mechanical stimulation via an external static magnetic field, which increased stem cell proliferation and facilitated their neural differentiation.

In this study, to achieve spatially controlled magnetic manipulation—precisely targeting magnetomechanical stimulation to the mechanosensitive cytoskeletal structures within SCs—we designed and synthesized a novel fluorescent-superparamagnetic superparticle with F-actin-specific biological targeting capability (Fe_3_O_4_·Cy3.5@PDA@phalloidin, termed f-SPIONs). These engineered f-SPIONs possess several key functional advantages: (1) Iron oxide nanoparticles are well-known for their excellent biosafety profile, and the addition of a polydopamine (PDA) shell further enhances their biocompatibility and affinity toward neural tissues. (2) Cyanine 3.5 (Cy3.5), a widely used red-emitting cyanine dye, provides bright and stable fluorescence for real-time in vivo tracking of f-SPION–cell interactions. (3) The Fe₃O₄ nanoparticles self-assemble into a superparticle magnetic core, exhibiting high saturation magnetization while preserving superparamagnetic behavior. Under an external magnetic field gradient, f-SPIONs demonstrate highly responsive magnetic behavior, allowing for precise and efficient delivery of mechanical forces to cells. (4) Surface functionalization with Phalloidin-Alexa Fluor 350—a highly specific probe for F-actin—enables selective targeting of the actin cytoskeleton. This targeted functionalization enhances the spatial resolution of magnetic force delivery, offering a promising approach for precision magnetomechanical stimulation at the subcellular level in SCs.

To enable temporal control of magnetic actuation, we designed and constructed a custom electromagnetic field generator. By harnessing the rapid field-generating capabilities of electromagnets and modulating the amplitude and waveform of the input current, we achieve high temporal resolution control over the gradient magnetic field. In combination with the superparamagnetic characteristics of f-SPIONs—which exhibit rapid magnetization under a gradient magnetic field and near-instantaneous demagnetization upon field removal owing to negligible remanence and coercivity—this system offers a robust platform for delivering spatiotemporally precise magnetomechanical stimulation to SCs.

In this study, we demonstrated that SCs within peripheral nerve tissues can perceive magnetomechanical stimulation mediated by f-SPIONs and transduce these mechanical cues into intracellular biochemical signals, thereby reprogramming gene and molecular expression profiles and ultimately inducing a functional phenotypic shift. This process—by which cells detect and convert mechanical stimuli into specific biological responses—is termed mechanotransduction.

However, magnetomechanical neuromodulation remains a nascent field, and the mechanotransduction mechanisms by which magnetic stimulation influences SC regenerative responses and phenotypic transitions are still poorly understood. The molecular mechanisms and signaling cascades that transduce magnetic inputs into intracellular biochemical signals remain largely undefined. In this study, in addition to confirming that f-SPION-mediated magnetomechanical stimulation modulates the SC repair phenotype, we systematically investigated the associated mechanotransduction mechanisms underlying this process.

### Cytoskeletal dynamics as the initiating event in cellular mechanotransduction

The cytoskeleton plays a fundamental role in enabling eukaryotic cells to withstand mechanical deformation, facilitating intracellular transport, and dynamically altering their morphology during migration [60, 87, 88]. As a highly dynamic architecture, the cytoskeleton functions as the principal mechanosensitive and load-bearing element of the cell, and is widely recognized as the physical foundation of cellular mechanotransduction [27, 59, 89]. Recent evidence indicates that both intrinsic and extrinsic mechanical forces can modulate cellular mechanical properties and behavior by targeting the cytoskeletal network.

Our findings demonstrate that f-SPIONs, by selectively binding to F-actin, can directly transduce magnetomechanical stimulation to the actin cytoskeletal framework. This targeted mechanical input elicits dynamic remodeling of actin filament organization and polymerization states, initiating a cascade of downstream responses—including Piezo1 ion channel upregulation and gating, as well as the activation of transmembrane Ca^2^⁺ signaling. These findings suggest that the cytoskeleton possesses the capacity to sense external mechanical stimuli—and to subsequently undergo dynamic modulation and structural reorganization—serves as the initiating event in the mechanotransduction cascade.

### Cytoskeletal dynamics-gated mechanosensitive Piezo1 channels as critical mediators of cellular mechanotransduction

Mechanosensitive ion channels (MSICs) are recognized as fundamental molecular sensors that detect and transduce mechanical stimuli across a wide range of cell types [90–92]. The recent identification of the evolutionarily conserved PIEZO channel family, including PIEZO1 and PIEZO2, has significantly advanced our understanding of how mechanical forces are sensed and converted into biological signals via mechanically gated cation channels [65, 93, 94]. Our results demonstrate that Piezo1, a key member of the MSIC family, has pronounced tissue-specific expression in the peripheral nervous system, particularly in SCs. These findings suggest that Piezo1 plays a pivotal role in mediating both mechanosensation and mechanotransduction in peripheral nerve tissues. In our experimental paradigm, f-SPION-mediated magnetic force stimulation led to a marked upregulation of Piezo1 expression in SCs. Previous studies have shown that PIEZO1 expression is subject to positive feedback regulation in response to mechanical cues [95], thereby supporting our observations.

Recent structural and functional studies of PIEZO channels have revealed that their unique structural plasticity enables direct detection of membrane curvature and tension, resulting in highly mechanosensitive and selective cation permeability [65, 96–98]. Owing to their intrinsic curvature-sensing and adaptive properties, activated PIEZO1 channels preferentially localize to plasma membrane regions with pronounced curvature. PIEZO1 has been reported to be expressed in astrocytic processes, where it likely serves as a mechanosensor that monitors the local microenvironment and initiates intracellular Ca^2^⁺ signaling [65]. Consistent with these findings, our study demonstrated that magnetic force-induced structural remodeling and morphological polarization in SCs led to significantly enhanced Piezo1 expression and localization within neurite-like protrusions than in the cell soma.

Actin-based cytoskeletal microfilaments are tethered to the inner leaflet of the plasma membrane through linker proteins—such as ankyrin and focal adhesion complexes—providing mechanical integrity and regulating membrane fluidity [99]. Concurrently, PIEZO channels interact directly with the actin cytoskeleton via mechanotransduction complexes—such as E-cadherin, β-catenin, and vinculin—enabling cytoskeleton-dependent gating modulation. We therefore hypothesize that magnetic force-induced activation of actin cytoskeletal dynamics transmits mechanical cues to membrane-resident Piezo1 channels, either directly through mechanotransduction complexes or indirectly by altering membrane tension, ultimately enhancing Piezo1 channel activity. In pharmacological rescue experiments, treatment with Cyto D or anisomycin effectively inhibited magnetic force-induced cytoskeletal activation and consequently abolished Piezo1 upregulation. These findings support our hypothesis that magnetic force-induced activation of actin cytoskeletal dynamics acts as an upstream regulatory mechanism that gates the mechanosensitive Piezo1 channel.

### Piezo1-triggered Ca^2^⁺ dynamics serve as a key regulator of cellular mechanotransduction

Calcium ions (Ca^2^⁺), as essential secondary messengers, regulate a broad range of biological processes, including gene transcription, mRNA translation, and post-translational protein modifications. To execute its signaling functions, intracellular Ca^2^⁺ is tightly compartmentalized, with cytosolic concentrations (100–300 nM) substantially lower than those in the extracellular milieu (1–3 mM) and endoplasmic reticulum stores (10–100 μM) [100]. In the context of neuroscience, calcium channels and their associated Ca^2^⁺ signaling dynamics are critical for neuronal excitability, functional maintenance, injury responses, and regenerative processes [101–103].

Piezo1, a mechanosensitive cation channel, plays a vital role in mediating transmembrane calcium influx ([Ca^2^⁺]_i_) [65]. Our findings revealed that transmembrane Ca^2^⁺ flux, driven by magnetically gated activation of Piezo1 channels, is a central regulator of mechanotransduction. This discovery aligns with the conclusions drawn by other scholars, who reported that “Ca^2^⁺ influx serves as an essential pathway for cells to achieve mechanosensation and mechanotransduction” [104, 105]. Pharmacological inhibition of Piezo1-mediated Ca^2^⁺ influx in rescue experiments abolished the magnetically induced characteristic structural reorganization and morphological polarization of SCs, as well as the activation of key transcription factors and signaling pathways. This finding confirms a clear upstream regulatory relationship between intracellular magnetically triggered Ca^2^⁺ signaling dynamics and the reprogramming process of the SC transition toward the repair phenotype.

## Conclusion

In this study, we developed a remote and noninvasive strategy for delivering high spatiotemporal resolution magnetomechanical stimulation to neural cells or tissues by using fluorescent-superparamagnetic superparticles functionalized for F-actin targeting (f-SPIONs) to apply piconewton-scale intracellular mechanical forces under an intermittent pulsed magnetic field. Within the magnetically actuated environment established in this study, SCs demonstrated highly responsive behavior characterized by transcriptional reprogramming toward a repair phenotype, which in turn facilitated peripheral nerve regeneration and functional recovery.

Mechanotransduction mechanism investigations revealed a potential signaling cascade whereby f-SPION-mediated magnetomechanical stimulation with temporal and spatial specificity activated actin cytoskeletal remodeling, which subsequently induced the mechanical activation of Piezo1 ion channels. This activation triggers transmembrane Ca^2^⁺ flux, with intracellular Ca^2^⁺ acting as a second messenger to regulate transcriptional reprogramming, ultimately actuating the phenotypic conversion of SCs into a repair state.

In summary, this work presents a comprehensive and innovative framework for elucidating how physical mechanical stimuli modulate the functional phenotype of SCs. These findings not only confirm that the peripheral nervous system perceives and responds to external mechanical cues during regeneration and repair, but also delineate a mechanotransduction pathway through which physical forces are transduced into biochemical signals that orchestrate nerve regeneration. This work advances our understanding of how living systems adaptively self-regulate via mechanotransduction, and introduces a novel “mechanotherapy” perspective and paradigm for future biomedical research and clinical treatment of peripheral nerve injuries.

## Supplementary Information

Additional file1

Additional file2

Additional file3

Additional file4

Additional file5

Additional file6

Additional file7

Additional file8

Additional file9

## Data Availability

The datasets generated and discussed in this publication have been deposited in NCBI’s Gene Expression Omnibus and the NCBI Short Read Archive. The data are accessible through GEO series accession numbers GSE305996 (https://www.ncbi.nlm.nih.gov/geo/query/acc.cgi?acc=GSE305996) and GSE306137 (https://www.ncbi.nlm.nih.gov/geo/query/acc.cgi?acc=GSE306137).

## Abbreviations

SCs: Schwann cells
rSCs: Repair Schwann cells
SPIONs: Superparamagnetic iron oxide nanoparticles
GMF: Gradient magnetic field
MNPs: Magnetic nanoparticles
f-SPIONs; Fe₃O₄·Cy3.5@PDA@phalloidin: Phalloidin F-actin–targeted functionalized superparamagnetic iron oxide nanoparticles
DMEM: Dulbecco’s modified Eagle’s medium
FBS: Fetal bovine serum
SD: Sprague Dawley
CO₂: Carbon dioxide
Cyto D: Cytochalasin D
OA: Oleic acid
NPs: Nanoparticles
OLA: Oleylamine
SPs: Superparticles
SDS: Sodium dodecyl sulfate
Cy3.5: Cyanine 3.5
O/W: Oil-in-water
DA: Dopamine
PDA: Polydopamine
f-SPIONs: Fe₃O₄**·**Cy3.5@PDA@phalloidin
HRTEM: High-resolution transmission electron microscopy
PDI: Polydispersity index
DLS: Dynamic light scattering
SQUID: Superconducting quantum interference device
PL: Photoluminescence
CLSM: Confocal laser scanning microscopy
TEM: Transmission electron microscopy
DC: Direct current
MST: Microscale thermophoresis
PBS: Phosphate-buffered saline
ROS: Reactive oxygen species
LPO: Lipid peroxidation
PI: Propidium Iodide
FSC: Forward scatter
SSC: Side scatter
cDNA: Complementary DNA
GAPDH: Glyceraldehyde-3-phosphate dehydrogenase
RIPA: Radioimmunoprecipitation assay
BCA: Bicinchoninic acid
TBST: Tris-buffered saline containing 0.1% Tween-20
PVDF: Polyvinylidene difluoride
SDS-PAGE: SDS–polyacrylamide gel electrophoresis
HRP: Horseradish peroxidase
ECL: Enhanced chemiluminescence
F-actin: Filamentous actin
G-actin: Globular actin
ROIs: Regions of interest
[Ca^2^⁺]_i_: Intracellular calcium concentration
***F***_***₀***_: Baseline fluorescence
***F***_***max***_: Peak fluorescence
ΔF: Variations in fluorescence intensity
Oi: Orientation index
OCT: Optimal cutting temperature
DAPI: 4′,6-Diamidino-2-phenylindole
PFA: Paraformaldehyde
Piezo1: Piezo-type mechanosensitive ion channel 1
BSA: Bovine serum albumin
GEO: Gene Expression Omnibus
SRA: Short Read Archive
DEGs: Differentially expressed genes
FDR: False discovery rate
GO: Gene Ontology
KEGG: Kyoto Encyclopedia of Genes and Genomes
GSEA: Gene Set Enrichment Analysis
NES: Normalized enrichment scores
SFI: Sciatic functional index
PL: Print length
TS: Toe spread
ITS: Intermediary toe spread
E: Experimental limb
N: Normal limb
CMAPs: Compound muscle action potentials
MNCV: Motor nerve conduction velocity
K–S: Kolmogorov–Smirnov
SNK test: Student–Newman–Keuls test
ANOVA: One-way analysis of variance
*∇****B***: Magnetic field gradient
***F***: Magnetic force
***B***: Magnetic flux density
***M***_***m***_: Mass magnetization
***M***_***s***_: Saturation magnetization
***m***: Magnetic dipole moment
***ρ***: Density
***V***: Volume
ICP-AES: Inductively coupled plasma atomic emission spectroscopy
Fe: Iron
$n_{cell}^{f - SPIONs}$: Number of f-SPIONs internalized per cell
$m_{cell}^{Fe}$: Iron mass per cell
$m_{nerve}^{Fe}$: Iron mass per nerve
$m_{f - SPION}^{Fe}$: Iron content per f-SPION
***F***_***f-SPION***_: Magnetic force exerted on a single f-SPION
***F***_***cell***_: Magnetic force exerted on a single cell
***F***_***nerve***_: Magnetic force exerted on the sciatic nerve
***m***_***nerve***_: Mass of sciatic nerve tissue
***m***_***Fe***_: Iron mass
$n_{nerve}^{f - SPIONs}$: Number of f-SPIONs within the sciatic nerve
RIN: RNA Integrity Number
***K***_***d***_: Dissociation constant
c-Jun: Jun proto-oncogene, AP-1 transcription factor subunit
STAT3: Signal transducer and activator of transcription 3
NCAM: Neural cell adhesion molecule
ECM: Extracellular matrix
D-type: Demyelinated degenerating fibers
DRG: Dorsal root ganglia
MSICs: Mechanosensitive ion channels
Ca^2^⁺: Calcium ions
[Ca^2^⁺]i: Calcium influx
OT: Optical tweezers
AFM: Atomic force microscopy
MPA: Micropipette aspiration
hiPSCs: Human induced pluripotent stem cells
mESCs: Mouse embryonic stem cells
