# Correction: f-SPION-mediated magnetic stimulation induces reparative Schwann cell reprogramming via cytoskeletal dynamics - gated activation of Piezo1

**DOI:** 10.1186/s12951-026-05167-7

**Published:** 2026-11-05

**Authors:** Ting Liu, Mingxi Yang, Wantao Tian, Jingyan Ren, Laijin Lu, Yang Wang

**Affiliations:** 1 Department of Geriatrics, The First Hospital of Jilin University, Changchun, 130021 P. R. China; 2 Department of Hand Surgery, Orthopedic Center, The First Hospital of Jilin University, Changchun, 130021 P. R. China; 3 Key Laboratory of Tissue Repair, Reconstruction and Regeneration of Jilin Province, Changchun, 130021 P. R. China

**Correction: Journal of Nanobiotechnology (2026) 24:298**

**https://doi.org/10.1186/s12951-026-04105-x**

Following publication of the original article, the authors identified two inadvertent image-placement errors introduced during figure assembly. In Fig. 6f, the DIC image from the f3 group (f-SPIONs + GMF + Cyto D) was inadvertently duplicated and placed in the DIC position of the f4 group (f-SPIONs + GMF + anisomycin). The duplicated image has been replaced with the correct original DIC image corresponding to the f4 group. The other fluorescence-channel images and the associated quantitative data remain unchanged. In Fig. 7h, the representative Piezo1 and corresponding GAPDH Western blot panel from Fig. 7c was inadvertently inserted during figure assembly. The incorrect panel has been replaced with the correct representative Piezo1 and corresponding GAPDH bands for the experimental groups shown in Fig. 7h: f-SPIONs + GMF, Cyto D, f-SPIONs + GMF + Cyto D, and f-SPIONs + GMF + anisomycin. The quantitative analysis and statistical results reported in the original Fig. 7h were derived from the correct original experimental data, comprising three independent experimental replicates, and not from the inadvertently inserted representative blot panel. Therefore, no changes to the quantitative results or statistical analyses are required. 

For completeness and transparency, the old incorrect and correct versions of Figs. 6 and 7 are displayed below.

Incorrect Fig. 6.

**Figure:**
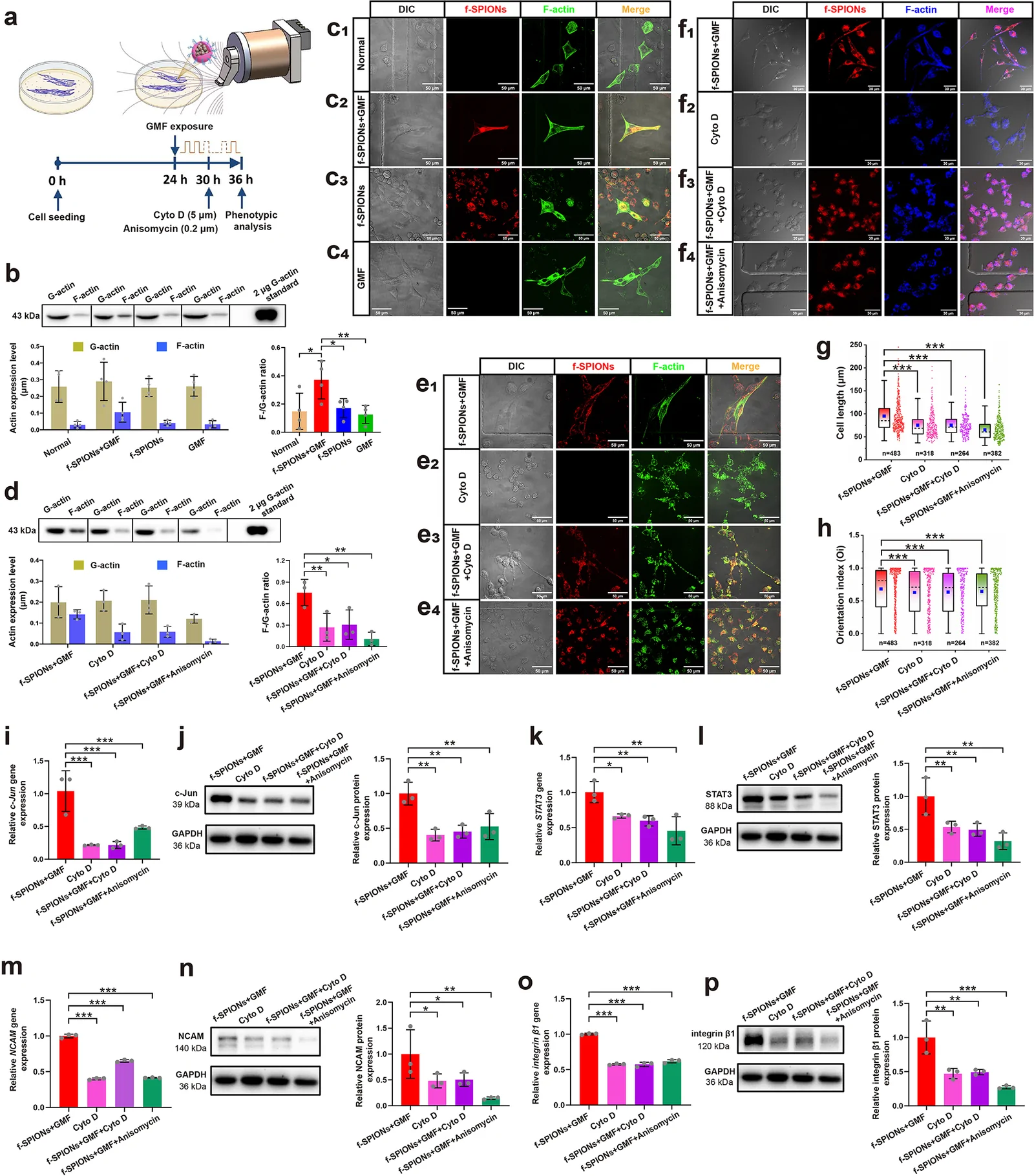

Correct Fig. 6.

**Fig. 6:**
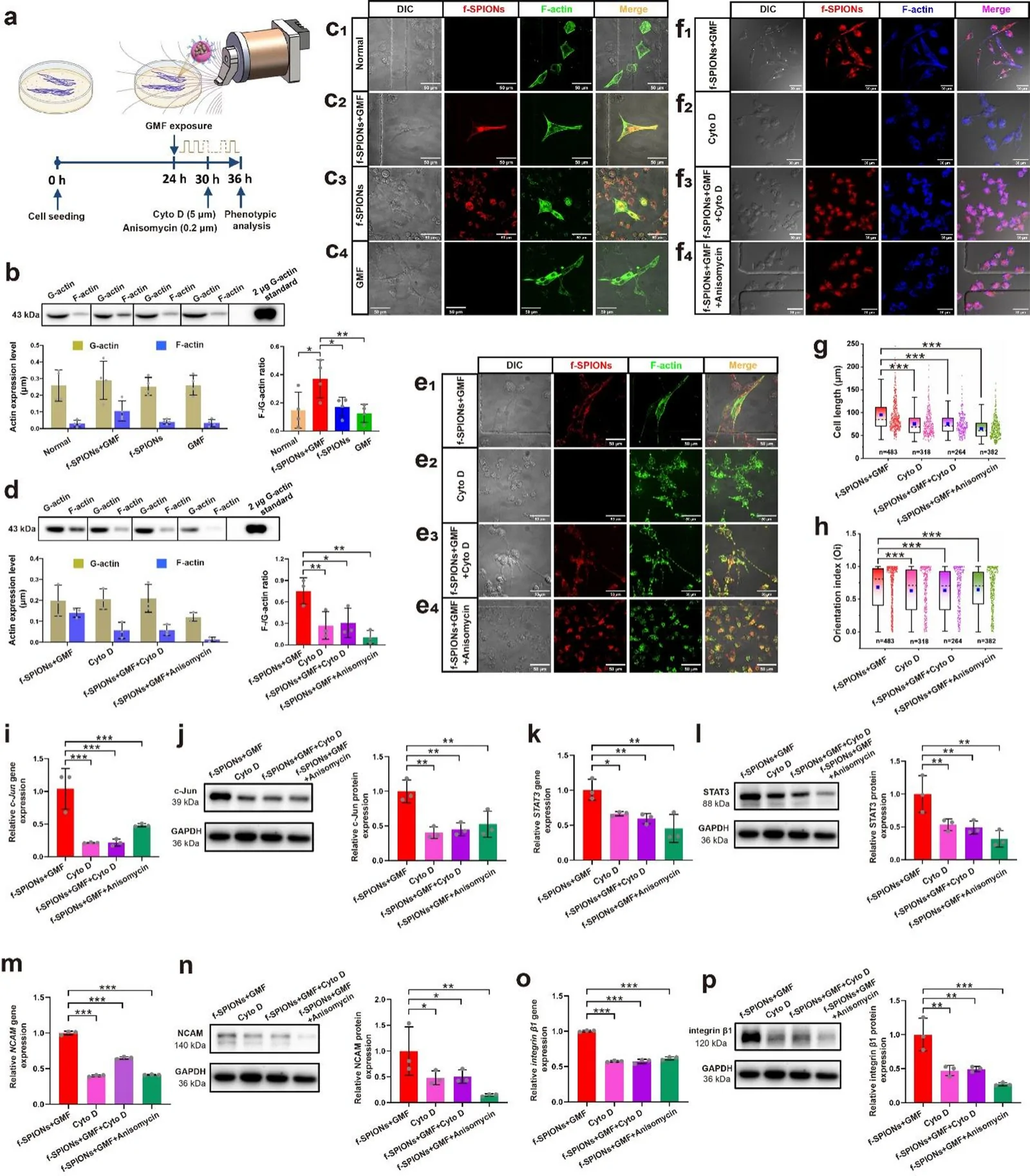
f-SPION-mediated magnetic actuation regulates SCs into a repair phenotype via activation of actin cytoskeletal dynamics **a**. Experimental workflow for investigating how magnetic force–dependent actin cytoskeletal dynamics regulate the SC repair phenotype. **b**. Western blot quantification of F-actin and G-actin expression levels and F/G-actin ratios in SCs from normal control, magnetic stimulation, f-SPION control, and magnetic field control groups. **c**. Live-cell actin staining revealed changes in F-actin cytoskeletal morphology under different conditions: (**c**_1_) normal control, (**c**_2_) magnetic stimulation, (**c**_3_) f-SPIONs only, and (**c**_4_) magnetic field only. **d**. Western blot quantification of F-actin and G-actin expression levels and F/G-actin ratios in SCs from the “f-SPIONs + GMF”, “Cyto D”, “f-SPIONs + GMF + Cyto D”, and “f-SPIONs + GMF + Anisomycin” groups. **e**. Live-cell actin staining visualized cytoskeletal dynamics under (**e**_1_) magnetic stimulation, (**e**_2_) Cyto D, (**e**_3_) magnetic stimulation + Cyto D, and (**e**_4_) magnetic stimulation + Anisomycin treatments. **f**. Immunofluorescence imaging of SCs showed structural remodeling and morphological polarization in (**f**_1_) “f-SPIONs + GMF”, (**f**_2_) “Cyto D”, (**f**_3_) “f-SPIONs + GMF + Cyto D”, and (**f**_4_) “f-SPIONs + GMF + Anisomycin” groups. **g**, **h**. Quantitative analysis of (**g**) cell length and (**h**) Oi under different treatments. **i**, **j**. Expression levels of transcription factor c-Jun assessed by (**i**) RT-qPCR and (**j**) Western blot. **k**,** l**. Expression levels of STAT3 measured by (**k**) RT-qPCR and (**l**) Western blot. **m**, **n**. NCAM expression analyzed by (**m**) RT-qPCR and (**n**) Western blot. **o**, **p**. Expression of integrin β1 evaluated by (**o**) RT-qPCR and (**p**) Western blot. Each experiment was independently repeated three or four times. “n” indicates the total number of cells measured. Statistical significance: * *p* < 0.05; ** *p* < 0.01; *** *p* < 0.001

Incorrect Fig. 7.

**Figure:**
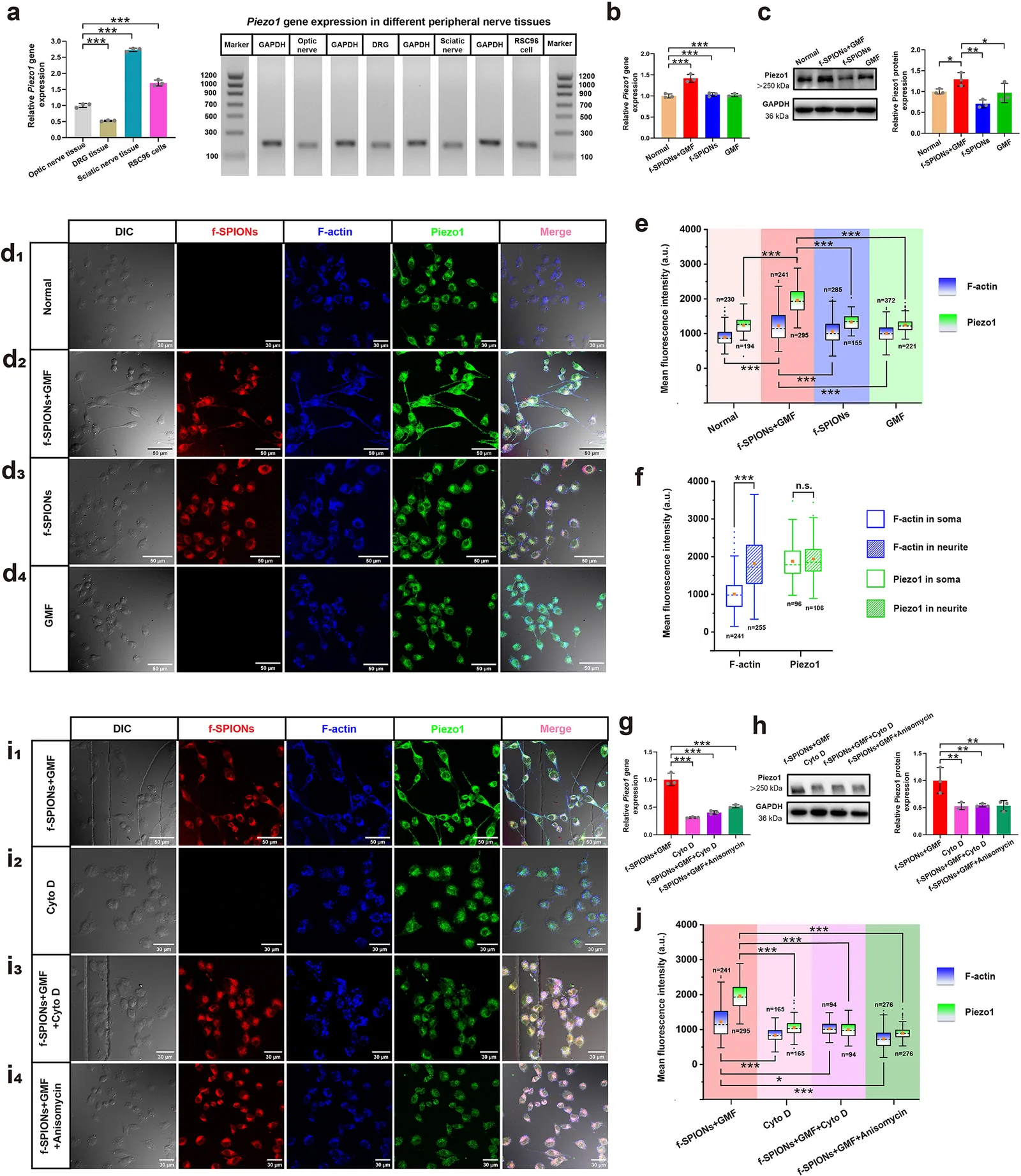

Correct Fig. 7.

**Fig. 7:**
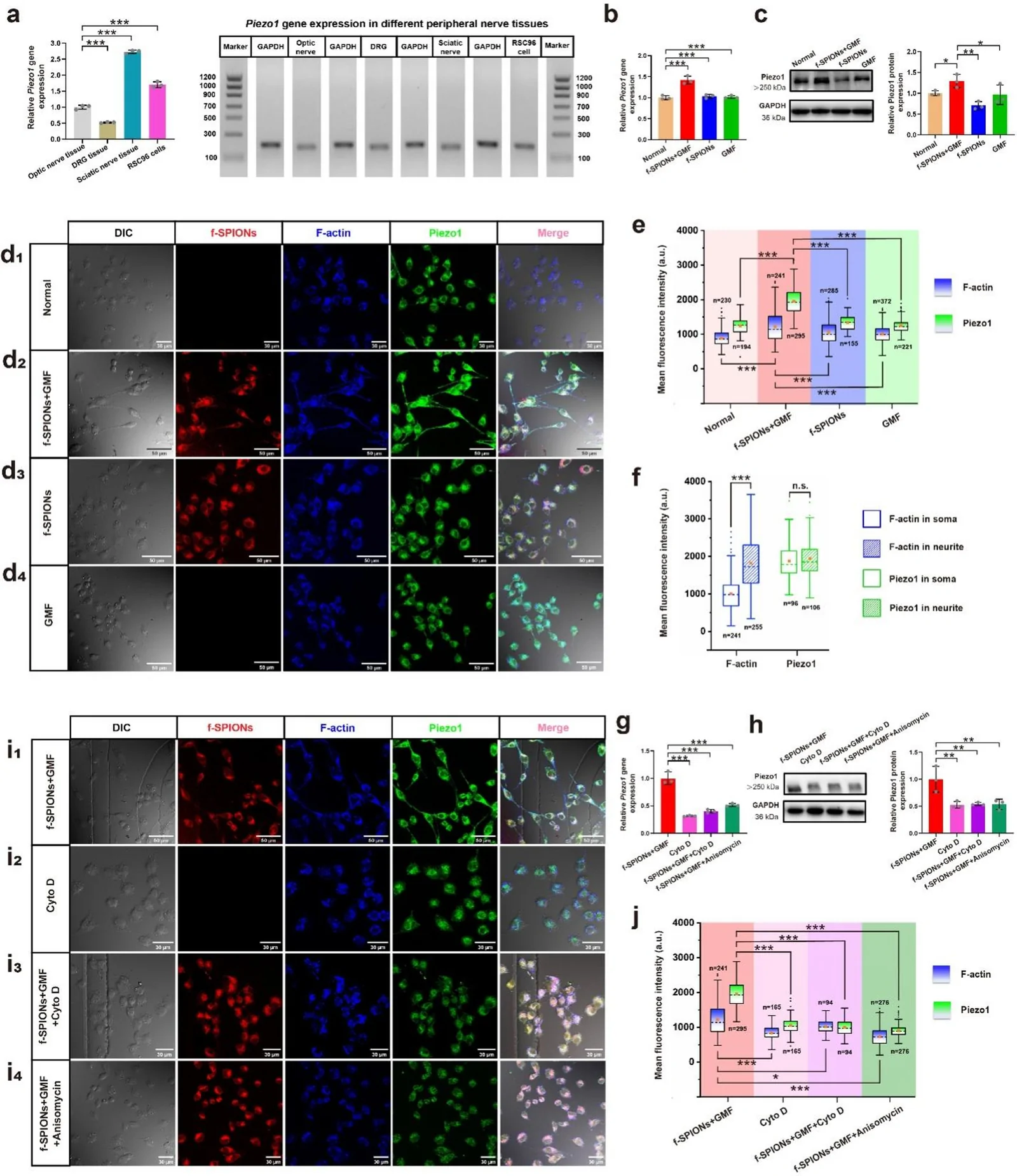
f-SPIONs-mediated magnetic actuation upregulates Piezo1 expression via activation of actin cytoskeleton dynamics. **a** RT-qPCR analysis of Piezo1 mRNA expression levels across different peripheral nerve tissues. **b**, **c** (**b**) RT-qPCR and (**c**) Western blot analyses of Piezo1 expression in Schwann cells from normal control, magnetic stimulation, f-SPIONs control, and magnetic field control groups. **d** Immunofluorescence imaging of F-actin and Piezo_1_ expression and subcellular distribution across different treatment groups: (**d**_1_) normal control, (**d**_2_) magnetic stimulation, (**d**_3_) f-SPIONs only, and (**d**_4_) magnetic field only groups. **e** Quantitative analysis of fluorescence intensity of F-actin and Piezo1 across different treatment groups. **f** Subcellular fluorescence distribution of F-actin and Piezo1 within soma and neurite compartments under magnetic stimulation. **g**, **h** Piezo1 expression levels measured by (**g**) RTqPCR and (**h**) Western blot in magnetic stimulation, Cyto D, magnetic + Cyto D, and magnetic + Anisomycin groups. **i** Immunofluorescence imaging of F-actin and Piezo1 expression in SCs treated with (**i**_1_) magnetic stimulation, (**i**_2_) Cyto D, (**i**_3_) magnetic stimulation + Cyto D, and (**i**_4_) magnetic stimulation + Anisomycin. **j** Quantitative comparison of F-actin and Piezo1 fluorescence intensity across different treatment groups. Data are representative of three independent replicates. “n” indicates the total number of cells measured. Statistical significance: * *p* < 0.05; ** *p* < 0.01; *** *p* < 0.001

The original article has been corrected.

