# Supplementary material for: f-SPION-mediated magnetic stimulation induces reparative Schwann cell reprogramming via cytoskeletal dynamics - gated activation of Piezo1
Source: J Nanobiotechnology. 2026 Feb 24;24:298. doi: 10.1186/s12951-026-04105-x (PMC13037106; doi:10.1186/s12951-026-04105-x)
Supplement: Supplementary file 1 — Additional file1 [file 12951_2026_4105_MOESM1_ESM.pdf]

Supplementary Information

f-SPION-Mediated Magnetic Stimulation Induces Reparative Schwann Cell Reprogramming via Cytoskeletal Dynamics–Gated Activation of Piezo1

Ting Liu[1], Mingxi Yang[2,3], Wantao Tian[2], Jingyan Ren[2,3], Laijin Lu[2,3], Yang Wang[2,3]*

[1] Department of Geriatrics, the First Hospital of Jilin University, Changchun 130021, P. R. China.

[2] Department of Hand Surgery, Orthopedic Center, the First Hospital of Jilin University, Changchun 130021, P. R. China.

[3] Key Laboratory of Tissue Repair, Reconstruction and Regeneration of Jilin Province, Changchun 130021, P. R. China.

* Corresponding Authors: Yang Wang

Address correspondence to wangy19851022@jlu.edu.cn

Table of the Electronic Supporting Information

1. Quantitative calculation of the nanoscale magnetic forces mediated by f-SPIONs

2. Quantitative estimation of f-SPION-mediated magnetic forces in Schwann cells

3. Quantitative evaluation of f-SPION-mediated magnetic forces in the sciatic nerve

4. Flow cytometric assessment of cytotoxicity induced by magnetic stimulation

5. RNA extraction, cDNA library construction, and transcriptome sequencing

6. Quality control and filtering of the clean reads from the RNA-seq data

7. In vivo quantification of G-actin and F-actin

Table of the Supplemental Figures

Supplementary Figure S1. Multiple TEM images showing the internalization of f-SPIONs within SCs

Supplementary Figure S2. Z-stack 3D reconstruction and cross-sectional imaging of f-SPION internalization in SCs

Supplementary Figure S3. CLSM imaging of f-SPION internalization by SCs

Supplementary Figure S4. Live-cell CLSM fluorescence imaging of the normal control and SPIONs control groups

Supplementary Figure S5. Live-cell F-actin tracing imaging of the f-SPIONs control and SPIONs-mediated magnetic stimulation groups

Supplementary Figure S6. Detection of ROS in SCs under magnetic stimulation mediated by f-SPIONs

Supplementary Figure S7. LPO detection in SCs under magnetic stimulation mediated by

f-SPIONs

Supplementary Figure S8. Evaluation of the cytotoxicity of magnetic stimulation using the Calcein/PI live/dead cell viability assay

Supplementary Figure S9. Evaluation of cytotoxicity of magnetic stimulation via Flow cytometry

Supplementary Figure S10. Body weight monitoring of rats following systemic intravenous administration of f-SPIONs

Supplementary Figure S11. Histopathological and pharmacokinetic evaluation of major organs following systemic intravenous administration of f-SPIONs

Supplementary Figure S12. F-/G-actin expression analysis in the SPIONs-mediated magnetic stimulation group

Table of the Supplementary Movies

Movie S1. Z-stack 3D reconstruction of f-SPIONs internalization by SCs

Movie S2. Magnetomechanical stimulation via f-SPIONs induces directional neurite outgrowth

Movie S3. SFI gait analysis video of crush injury control and magnetic stimulation groups at 3 days post-surgery

Movie S4. SFI gait analysis video of crush injury control and magnetic stimulation groups at 7 days post-surgery

Movie S5. SFI gait analysis video of crush injury control and magnetic stimulation groups at 14 days post-surgery

Movie S6. Magnetomechanical stimulation via f-SPIONs activates intracellular Ca^{2+} dynamics in Schwann cells

Movie S7. Activation of actin cytoskeleton dynamics regulates Ca^{2+} responses in Schwann cells

Movie S8. Piezo1 functional activation induces intracellular Ca^{2+} dynamics in Schwann cells

Table of the Supplementary Tables

Table S1. Chemical and peptide reagent information used in this study

Table S2. ICP-AES detection of endogenous iron content in Schwann cells of the normal control group

Table S3. ICP-AES detection of exogenous iron content in Schwann cells from the magnetic stimulation group

Table S4. Sequences of the primers used in this study

Table S5. Antibody reagent information used in this study

1. Quantitative calculation of the nanoscale magnetic forces mediated by f-SPIONs

In a spatially nonuniform magnetic field, a magnetic field gradient ($\nabla \boldsymbol{B}$, in T/m) arises, exerting a force on magnetic nanoparticles (MNPs) possessing a magnetic dipole moment ($\boldsymbol{m}$). Under such conditions, the magnetic force ($\boldsymbol{F}$) experienced by the nanoparticles is defined as:

$$F = (m \cdot \nabla)B \qquad \text{Equation (1)}$$

In this study, the spatial gradients of magnetic flux density (B, in tesla) generated by the magnetic field apparatuses were quantified by measuring the horizontal rate of change (***dB/dr***). The GMF produced by the electromagnetic coil for in vitro cell experiments was determined to be 3.25 T/m **(Fig. 1d)**, whereas the field generated by the neodymium magnet system for in vivo animal experiments reached 16.0 T/m **(Fig. 1g)**. Magnetic characterization of the f-SPIONs revealed negligible remanence and coercivity, confirming their pronounced superparamagnetic behavior. In superparamagnetic nanoparticles, the magnetic dipole moment ($\boldsymbol{m}$) aligns parallel to the direction of the applied magnetic field under equilibrium conditions. Under these ideal conditions, the magnetic force equation can be simplified to a scalar form:

$$F_{f\text{-}SPION}=m\frac{dB}{dr} \qquad \text{Equation (2)}$$

As shown in the magnetic hysteresis loop of the f-SPIONs **(Fig. 2g)**, the saturation magnetization ($\boldsymbol{M_s}$) reached approximately 35 Am ?kg under an applied magnetic field of 8000 Oe (0.8 T). On the basis of both simulation and empirical measurements, the maximum

magnetic field strengths generated within the in vitro and in vivo magnetic stimulation devices were approximately 82.5 mT and 200 mT, respectively. Under these magnetic field conditions, the mass magnetization (***M_m***) of the f-SPIONs was estimated to be approximately 16 Am^2/kg for the in vitro setup and 26 Am^2/kg for the in vivo configuration. The magnetic dipole moment (***m***) of an individual f-SPION can be determined by the product of its mass magnetization (***M_m***), material density (***ρ***), and particle volume (***V***), according to the following relation:

$$m = \rho \cdot V \cdot M_m \qquad \text{Equation (3)}$$

High-resolution transmission electron microscopy (HRTEM) analysis revealed that the f-SPIONs had an average overall diameter of 63.36 nm, comprising a spherical magnetic Fe_3O_4 core of approximately 56 nm in diameter and a surface shell composed of polydopamine (PDA) and phalloidin–Alexa Fluor 350, with an average thickness of 5.79 nm **(Fig. 2b–2e)**. Using the standard formula for the volume of a sphere ($V = 4/3\pi r^3$), the calculated volume of the magnetic core was approximately 7.36×10^{-17} cm^3. Given that Fe_3O_4 constituted approximately 80% of the core material, the estimated volume specifically attributable to Fe_3O_4 was 5.89×10^{-17} cm^3. The density (***ρ***) of the magnetic Fe_3O_4 core was 5.24 g/cm^3, in accordance with standard material constants. On the basis of these parameters, the net magnetic force exerted on a single f-SPION (***$F_{f\text{-}SPION}$***) under the specified magnetic field gradient was calculated via the following equation:

$$F_{f\text{-}SPION} = \rho \cdot V \cdot M_m \frac{dB}{dr} \qquad \text{Equation (4)}$$

where:

$\boldsymbol{F_{f\text{-}SPION}}$ denotes the magnetic force (N) exerted on an individual f-SPION within the magnetic field gradient (dB/dr).

$\boldsymbol{dB/dr}$ is the magnetic field gradient (T/m) generated by the corresponding magnetic field generation device.

$\boldsymbol{M_m}$ represents the mass magnetization of the f-SPIONs (Am^2/kg) under the given field intensity;

$\boldsymbol{\rho}$ is the density of Fe_3O_4 (5.24 g/cm^3).

V is the volume of the Fe_3O_4 magnetic core within each f-SPION (cm^3).

On the basis of Equation (4), the calculated magnetic force ($\boldsymbol{F_{f\text{-}SPION}}$) acting on each f-SPION under the 3.25 T/m field gradient produced by the electromagnetic coil in the in vitro experiments was approximately 1.59×10^{-5} pN **(Fig. 1e)**. In contrast, under the 16.0 T/m field gradient generated by the neodymium magnet assembly for in vivo studies, the magnetic force increased to approximately 1.27×10^{-4} pN.

2. Quantitative estimation of f-SPION-mediated magnetic forces in Schwann cells

The volume of Fe_3O_4 within the magnetic core of each f-SPION was estimated to be approximately 5.89×10^{-17} cm^3, with a material density of 5.18 g/cm^3. On the basis of these parameters, the mass of Fe_3O_4 per individual f-SPION was calculated to be approximately 3.05×10^{-4} pg. Taking into account that iron (***Fe***) constitutes 72.36% of the molar mass of Fe_3O_4, the iron content per f-SPION ($\boldsymbol{m^{Fe}_{f\text{-}SPION}}$) was estimated at approximately 2.21×10^{-4} pg.

RSC96 cells were plated at a density of 5 × 10^5 cells per well in 6-well culture plates and incubated for 24 hours. The cells were subsequently coincubated with 15 μg/mL f-SPIONs for an additional 24 hours. Following incubation, the cells were washed twice with ice-cold PBS supplemented with 1 mM deferoxamine to remove noninternalized, membrane-bound iron. The cells were then collected and counted. The intracellular endogenous iron content was quantified via inductively coupled plasma atomic emission spectroscopy (ICP-AES) on a PerkinElmer Optima 3300 DV instrument **(Table S2)**. The mean mass of internalized exogenous iron per RSC96 cell ($\boldsymbol{m_{cell}^{Fe}}$) was determined to be 2.05 ± 0.64 pg/cell on the basis of the total cell count **(Table S3)**.

Dividing the average iron content per cell ($\boldsymbol{m_{cell}^{Fe}}$) by the iron mass per f-SPION ($\boldsymbol{m_{f\text{-}SPION}^{Fe}}$) yielded the number of f-SPIONs internalized per cell ($\boldsymbol{n_{cell}^{f\text{-}SPIONs}}$). These calculations revealed that, RSC96 cells internalized approximately 9.30 ± 2.88 × 10^3 f-SPIONs per cell following 15 μg/mL f-SPION exposure.

The total magnetic force exerted on a single Schwann cell ($\boldsymbol{F_{cell}}$) was derived by multiplying the force per f-SPION ($\boldsymbol{F_{f\text{-}SPION}}$) by the number of particles internalized per cell ($\boldsymbol{n_{cell}^{f\text{-}SPIONs}}$), as shown in Equation (5):

$$F_{cell}=n_{cell}^{f\text{-}SPIONs}\cdot F_{f\text{-}SPION} \qquad \text{Equation (5)}$$

Accordingly, under the 3.25 T/m magnetic field gradient generated by the in vitro electromagnetic device, the estimated mechanical force exerted on each Schwann cell due to f-SPION internalization was approximately 0.15 ± 0.05 pN **(Fig. 1e)**.

Table S2. ICP-AES detection of endogenous iron content in Schwann cells of the normal control group

Sample ID	Total number of cells in sample	Iron mass detected in sample by ICP-AES (pg)	Endogenous iron content per cell (pg/cell)
No. 1	3.18×10^{6}	0.253×10^{6}	0.080
No. 2	3.96×10^{6}	0.249×10^{6}	0.063
No. 3	3.66×10^{6}	0.262×10^{6}	0.072
Mean ± SD			0.072 ± 0.008

Table S3. ICP-AES detection of exogenous iron content in Schwann cells from the magnetic stimulation group

Sample ID	Total number of cells in sample	Iron mass detected in sample by ICP-AES (pg)	The total iron content in each cell (pg/cell)[a]	Exogenous iron content per cell (pg/cell)[b]
No. 1	12.50×10^{5}	1.991×10^{6}	1.588	1.516
No. 2	9.27×10^{5}	2.624×10^{6}	2.831	2.759
No. 3	9.60×10^{5}	1.882×10^{6}	1.960	1.888
Mean ± SD			2.126 ± 0.638	2.054 ± 0.638

[a] Includes both endogenous iron and iron from internalized exogenous f-SPIONs.

[b] Calculated as the total cellular iron minus the endogenous iron in normal cells.

3. Quantitative evaluation of f-SPION-mediated magnetic forces in the sciatic nerve

ICP-AES was employed to quantify the iron content in sciatic nerve tissues at various time points following epineurial administration of f-SPIONs (200 μg/mL, 25 μL). The relative iron concentrations (iron mass per gram of nerve tissue, $\boldsymbol{m_{Fe}/m_{nerve}}$) at 1, 3, 7, and 14 days post-injection were measured to be 434.42 ± 37.12 μg/g, 231.77 ± 20.06 μg/g, 145.41 ± 40.19 μg/g, and 105.50 ± 17.39 μg/g, respectively. In the untreated control group, the baseline iron content was 37.73 ± 1.60 μg/g. By subtracting the baseline values, the residual exogenous iron content in nerve tissues at 1, 3, 7, and 14 days post-injection was estimated to be

396.72 ± 37.12 μg/g, 194.07 ± 20.06 μg/g, 107.71 ± 40.19 μg/g, and 67.80 ± 17.39 μg/g, respectively.

Using the recorded mass of sciatic nerve tissue ($\boldsymbol{m_{nerve}}$), the absolute amount of residual exogenous iron ($\boldsymbol{m^{Fe}_{nerve}}$) was calculated as 3.57 ± 0.33 μg, 2.13 ± 0.22 μg, 1.40 ± 0.52 μg, and 0.34 ± 0.09 μg at 1, 3, 7, and 14 days post-injection, respectively **(Fig. 1j)**. By dividing the total iron mass ($\boldsymbol{m^{Fe}_{nerve}}$) by the mass of a single f-SPION ($\boldsymbol{m^{Fe}_{f\text{-}SPION}}$), the number of f-SPIONs retained in nerve tissue ($\boldsymbol{n^{f\text{-}SPIONs}_{nerve}}$) was estimated to be $1.62 \pm 0.15 \times 10^{10}$, $0.97 \pm 0.10 \times 10^{10}$, $0.63 \pm 0.24 \times 10^{10}$, and $0.15 \pm 0.04 \times 10^{10}$ at 1, 3, 7, and 14 days, respectively **(Fig. 1j)**.

The total magnetic force exerted on the sciatic nerve ($\boldsymbol{F_{nerve}}$) was calculated by multiplying the magnetic force of an individual f-SPION ($\boldsymbol{F_{f\text{-}SPION}}$) by the number of f-SPIONs within the tissue ($\boldsymbol{n^{f\text{-}SPIONs}_{nerve}}$):

$$F_{nerve} = n^{f\text{-}SPIONs}_{nerve} \cdot F_{f\text{-}SPION} \qquad \text{Equation (6)}$$

As established in Equation (4), each f-SPION exposed to the gradient magnetic field generated by the annular NdFeB magnet array exerted an estimated force ($\boldsymbol{F_{f\text{-}SPION}}$) of approximately 1.27×10^{-4} pN. According to Equation (6), the cumulative magnetic force ($\boldsymbol{F_{nerve}}$) exerted on the sciatic nerve by the internalized f-SPIONs was estimated to be 2.05 ± 0.19 μN, 1.23 ± 0.13 μN, 0.80 ± 0.30 μN, and 0.19 ± 0.05 μN at 1, 3, 7, and 14 days post-epineurial injection, respectively **(Fig. 1j)**.

4. Flow cytometric assessment of cytotoxicity induced by magnetic stimulation

Flow cytometric gating strategy for RSC96 cells

To ensure accurate identification of live, single-cell populations, a standardized flow cytometric gating strategy was applied to all samples. First, cell debris and non-cellular events were excluded based on forward scatter (FSC) and side scatter (SSC) characteristics in the FSC-A vs. SSC-A plot. Next, single cells were distinguished from doublets and aggregates by gating along the FSC-H vs. FSC-A plot, thereby ensuring that only single-cell events were included in subsequent analyses.

Flow cytometric detection of intracellular fluorescence

The internalized fluorescence derived from Cy3.5-labeled f-SPIONs (Ex/Em: 561/607 nm, PE channel) was quantified using a CytoFLEX S flow cytometer (Beckman Coulter, Brea, CA, USA) equipped with a 561 nm laser. For each sample, at least 10,000 single-cell events were recorded. Background fluorescence was subtracted based on the signal obtained from untreated control cells, and all measurements were performed under identical detector gain and voltage settings. Fluorescence intensity were analyzed using FlowJo software (version 10.9.0, Becton Dickinson & Company, Franklin Lakes, NJ, USA). The mean fluorescence intensity of each group was calculated to evaluate the relative degree of f-SPIONs uptake by Schwann cells.

Flow cytometric analysis of live and dead cells

To establish clear discrimination between live and dead cell populations, an apoptosis positive control was prepared by resuspending normally cultured RSC96 cells in the Apoptosis Positive Control Solution provided in the Annexin V-APC/7-AAD Apoptosis Detection Kit (Lianke Bio, Cat. # AP105, Hangzhou, China). The cells were incubated on ice

for 30 min and then washed twice with pre-chilled PBS before being mixed with untreated live cells to serve as a positive control.

Dead cells were identified using the Live or Dead™ Fixable Dead Cell Staining Kit (Green Fluorescence, AAT Bioquest, Cat. #CS22501, USA; Ex/Em: 488/525–540 nm, FITC channel). Briefly, cells were incubated with the working staining solution at room temperature for 30 min in the dark, followed by two washes with flow cytometry buffer (PBS containing 1% BSA and 0.1% sodium azide). Flow cytometric data were acquired using a CytoFLEX S flow cytometer (Beckman Coulter, Brea, CA, USA) equipped with a 488 nm laser, and analyzed using FlowJo software (version 10.9.0, Becton Dickinson & Company, Franklin Lakes, NJ, USA).

5. RNA extraction, cDNA library construction, and transcriptome sequencing

Total RNA was extracted via TRIzol reagent (Invitrogen, Thermo Fisher Scientific, Cat. #15596026, Waltham, MA, USA) according to the manufacturer's instructions. The RNA concentration and integrity were assessed via an Agilent 2100 Bioanalyzer and an RNA 6000 Nano LabChip Kit (Agilent Technologies, Cat. #5067-1511, Santa Clara, CA, USA). Only RNA samples with an RNA integrity number (RIN) greater than 7.0 were used for subsequent library construction. Polyadenylated mRNA was isolated from 5 μg of total RNA via Dynabeads Oligo (dT) (Thermo Fisher Scientific, Cat. #61005, Waltham, MA, USA) through two rounds of magnetic purification. The purified mRNA was thermally fragmented using divalent cations at 94 ℃ for 5–7 minutes with the Magnesium RNA Fragmentation Module (New England Biolabs, Cat. #E6150, Ipswich, MA, USA). The fragmented RNA was reverse

transcribed into first-strand cDNA via SuperScript™ II Reverse Transcriptase (Invitrogen, Thermo Fisher Scientific, Cat. #1896649, Waltham, MA, USA). Second-strand cDNA synthesis was performed using *E. coli* DNA Polymerase I (New England Biolabs, Cat. #M0209, Ipswich, MA, USA), RNase H (New England Biolabs, Cat. #M0297, Ipswich, MA, USA), and dUTP solution (Thermo Fisher Scientific, Cat. #R0133, Waltham, MA, USA). An adenine (A) nucleotide was added to the 3' blunt ends of the DNA fragments to facilitate adapter ligation. Adapters containing a single thymine (T) overhang were ligated to the A-tailed DNA fragments. Adapter-ligated products were subjected to size selection via AMPure XP magnetic beads (Beckman Coulter, Cat. #A63880, Brea, CA, USA). The uracil-containing second-strand cDNA was treated with heat-labile uracil-DNA glycosylase (UDG; New England Biolabs, Cat. #M0280, Ipswich, MA, USA), followed by PCR amplification under the following conditions: initial denaturation at 95 ℃ for 3 minutes; 8 cycles of 98 ℃ for 15 seconds, 60 ℃ for 15 seconds, and 72 ℃ for 30 seconds; and a final extension at 72 ℃ for 5 minutes. The final cDNA libraries had an average insert size of approximately 300 ± 50 bp. Paired-end sequencing (2 × 150 bp) was performed on the Illumina NovaSeq™ 6000 platform, following the manufacturer's standard protocol.

6. Quality control and filtering of the clean reads from the RNA-seq data

cDNA libraries were constructed from mixed RNA samples derived from RSC96 cells and rat sciatic nerve tissues via the Illumina NovaSeq™ 6000 sequencing platform. Transcriptome sequencing was performed via paired-end RNA-seq (2 × 150 bp), which generated approximately one million paired-end reads. The raw reads obtained from the

sequencer contained adapter contaminants and low-quality bases, which could interfere with downstream transcriptome assembly and analysis. To obtain high-quality clean reads, the raw sequences were filtered via Cutadapt (v1.9; https://cutadapt.readthedocs.io/en/stable/). The filtering criteria were as follows: (1) reads containing adapter sequences were removed; (2) reads with poly (A) or poly (G) homopolymeric stretches were excluded; (3) reads containing more than 5% ambiguous nucleotides (N) were filtered out; and (4) reads with more than 20% low-quality bases (Q-score $\leq$ 20) were discarded. Sequence quality was subsequently assessed via FastQC (v0.11.9; http://www.bioinformatics.babraham.ac.uk/projects/fastqc/). Key quality metrics, including the Q20, Q30, and GC contents, were calculated on the basis of the filtered clean reads. As a result, a total of 75.87 Gbp of high-quality, paired-end clean reads were obtained. The raw sequencing data have been deposited in the NCBI Gene Expression Omnibus (GEO; accession numbers: GSE305996 and GSE306137).

7. In vivo quantification of G-actin and F-actin

The lysis buffer was prepared following the manufacturer's protocol by supplementing 1 mL of lysis and F-actin stabilization buffer (Part #LAS01) with 10 μL of ATP stock solution (100 mM, Cat. #BSA04) and 10 μL of protease inhibitor cocktail (100×, Cat. #PIC02). The prepared lysis buffer was prewarmed to 37 ℃ prior to application. The culture medium was aspirated, and the adherent cells were rinsed three times with PBS. An appropriate volume of the prewarmed lysis buffer was then added to each culture dish. For nerve tissue samples, 1 mL of prewarmed lysis buffer was applied per 100 mg of tissue. The lysates were incubated

at 37 ℃ for 10 minutes, after which 100 μL from each sample was collected for subsequent analysis.

The collected lysates (100 μL) were centrifuged at 350 × g for 5 minutes at room temperature to pellet unbroken cells and tissue debris. The supernatant was carefully collected and transferred into prelabeled ultracentrifuge tubes. The samples were then ultracentrifuged at 100,000 × g at 37 ℃ for 1 hour to sediment F-actin, leaving G-actin in the supernatant. The G-actin–enriched supernatant was transferred into newly labeled microcentrifuge tubes. The F-actin pellet was resuspended in 100 μL of depolymerization buffer (Part #FAD02) and incubated on ice for 1 hour to ensure complete depolymerization. To promote uniform resuspension, the pellet was gently pipetted every 15 min during the incubation period. Subsequently, 25 μL of 5× SDS sample buffer was added to both the F-actin and G-actin fractions and mixed thoroughly.

Both G-actin and F-actin lysates were separated by SDS–polyacrylamide gel electrophoresis (SDS–PAGE). Proteins were subsequently transferred onto a PVDF membrane following the manufacturer’s guidelines. Following transfer, the membrane was blocked in TBST containing 5% nonfat milk for 30 minutes at room temperature. A mouse monoclonal anti-actin antibody (provided in the kit) was diluted 1:1000 in TBST. The membrane was incubated with the primary antibody for 1 hour at room temperature. The membrane was subsequently washed three times in TBST for 10 minutes each at room temperature. According to the manufacturer's instructions, HRP-conjugated anti-mouse IgG (1:10,000, Cat. #AAN02-S) was applied for 1 hour at room temperature. The membrane was then washed five times with TBST, each for 10 minutes at room temperature. Actin (43 kDa)

was visualized by chemiluminescence via standard ECL detection on a PVDF membrane. The expression levels of G-actin and F-actin were quantified via densitometric analysis against a G-actin standard curve.

Supplementary Figure S1. TEM images showing the internalization of f-SPIONs within SCs.

a-d. TEM images obtained from multiple independent experiments demonstrate the internalization of f-SPIONs by SCs (RSC96 cells) following cellular uptake. ***Orange arrow***s indicate the cell membrane, ***blue arrows*** indicate the f-SPIONs internalized by the cells, ***green arrows*** indicate the mitochondria. **Abbreviations:** SC, Schwann cell; Nuc, nucleus; Mem, cytomembrane; MTs, mitochondria.

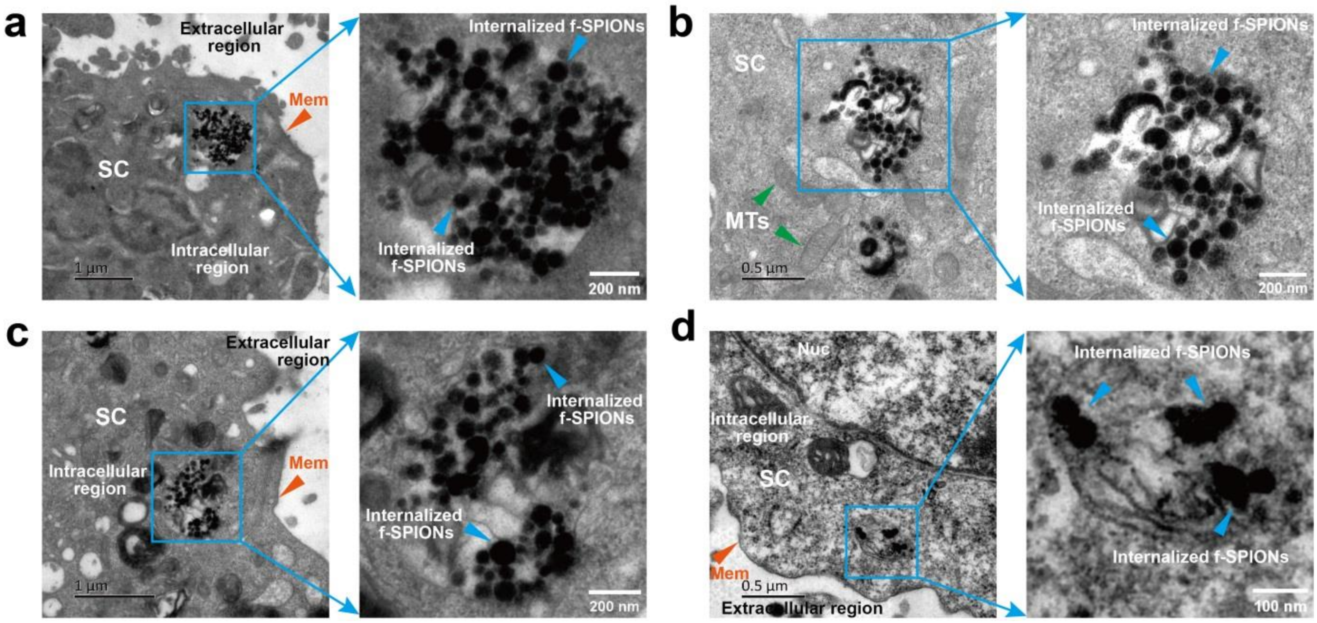

Supplementary Figure S2. Z-stack 3D reconstruction and cross-sectional imaging of f-SPION internalization in SCs

a–d. Different views of Z-stack 3D reconstruction. **e–i.** Views of different Z-axis cross-sections. Z-stack thickness: 16.0 μm; cross-sectional imaging was performed at 1 μm intervals along the Z-axis. Imaging parameters: pinhole = 1 Airy unit, z-step = 0.43 μm. Cell membranes were stained with DiO (Ex/Em: 494/517 nm); f-SPIONs were imaged using the standard Cy3.5 excitation channel in CLSM (Ex/Em: 561–568/607 nm).

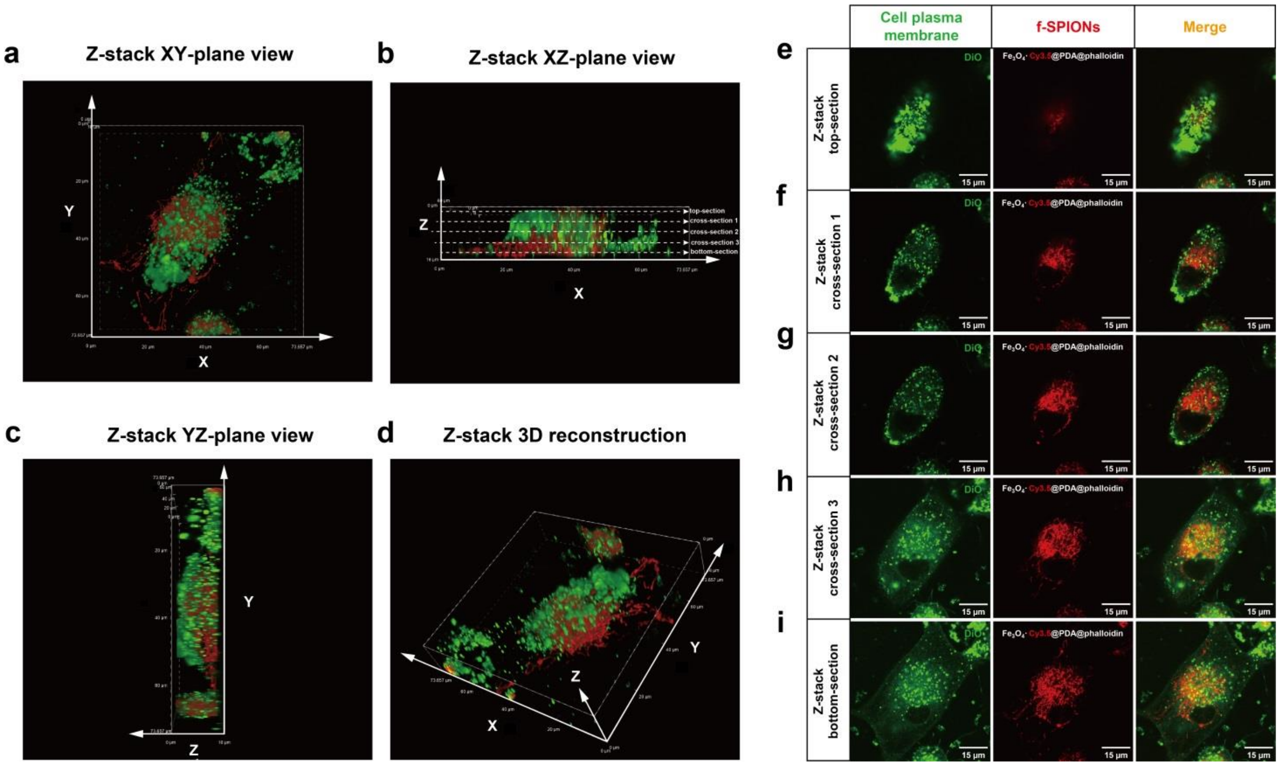

Supplementary Figure S3. CLSM imaging of f-SPION internalization by SCs.

a–c. Representative regions of interest (ROIs) obtained from three independent experiments. The cell membranes were labeled with DiO (green fluorescence, Ex/Em: 494/517 nm), and the f-SPIONs exhibited intrinsic red fluorescence (Ex/Em: 561–568/607 nm). The red fluorescence of f-SPIONs was completely enclosed by the DiO-labeled plasma membrane, demonstrating effective cellular uptake and internalization of f-SPIONs by Schwann cells (RSC96). ***White arrows*** indicate the continuous plasma membrane structures. **CLSM imaging parameters:** pinhole = 1 Airy unit; z-step = 0.43 μm.

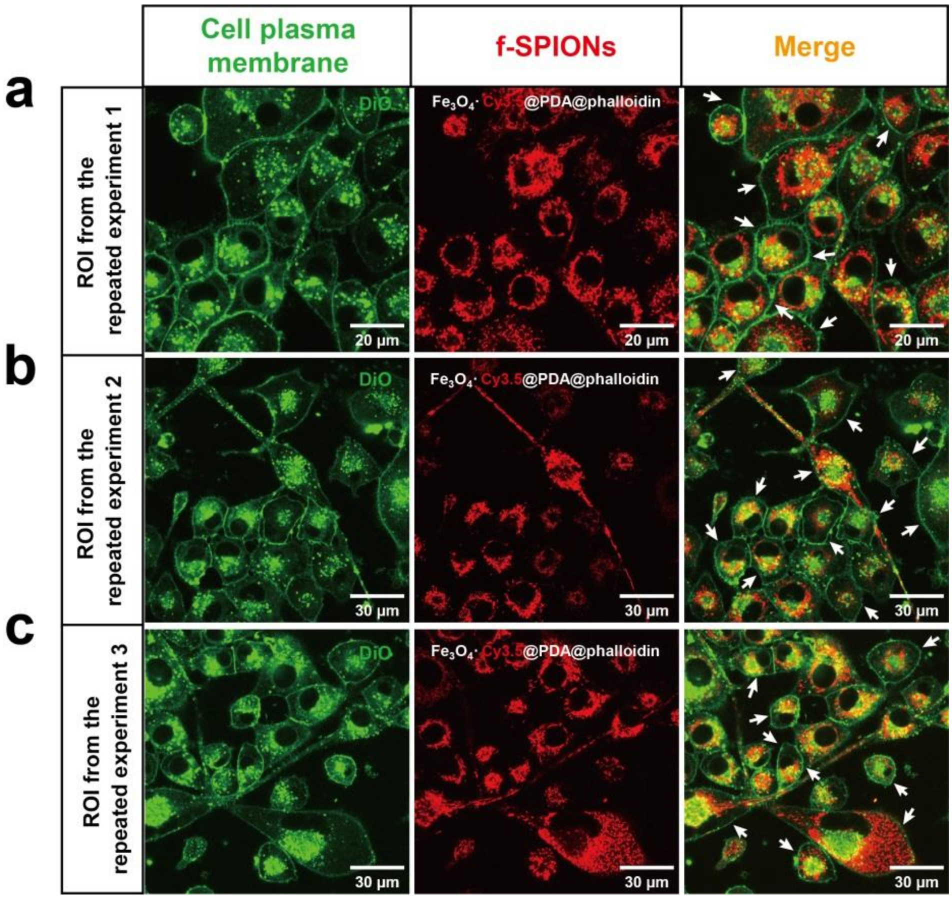

Supplementary Figure S4. Live-cell CLSM fluorescence imaging of the normal control and SPIONs control groups

a. Fluorescence imaging of cells in the normal control group. **b.** Fluorescence imaging of cells in the SPIONs control group. The red fluorescence channel uses the standard Cy3.5 excitation mode of CLSM (excitation at 561–568 nm, emission observed at 590–611 nm). The blue fluorescence channel uses the standard Alexa Fluor 350 excitation mode of CLSM (excitation at 350–360 nm, emission observed at 440–460 nm). Cell nuclei are labeled with Hoechst dye.

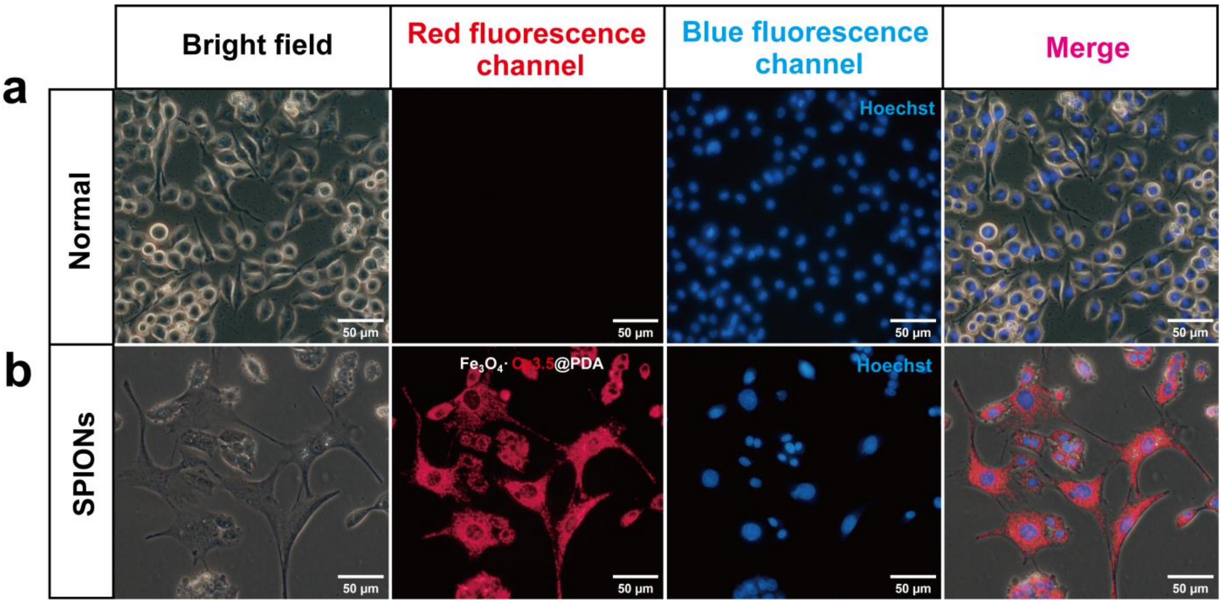

Supplementary Figure S5. Live-cell F-actin tracing imaging of the f-SPIONs control and SPIONs-mediated magnetic stimulation groups

a. Live-cell F-actin tracing imaging of Schwann cells in the f-SPIONs control group. **b.** Co-localization trend of f-SPIONs red fluorescence and F-actin green fluorescence within Schwann cells (blue dashed box in Fig. a) in the f-SPIONs control group (Pearson's R value: 0.57). **c.** Live-cell F-actin tracing imaging of Schwann cells in the SPIONs-mediated magnetic stimulation group (SPIONs+GMF). **d.** No co-localization of SPIONs red fluorescence and F-actin green fluorescence within cells (blue dashed box in Fig. c) in the SPIONs magnetic stimulation group (Pearson's R value: 0.29). ***White arrows*** indicate the direction of the GMF. **e.** Quantitative analysis of the Pearson's correlation coefficient between the red fluorescence of SPIONs or f-SPIONs and the green fluorescence of F-actin. "n" indicates the number of cells observed and analyzed. Statistical significance: *** $p < 0.001$.

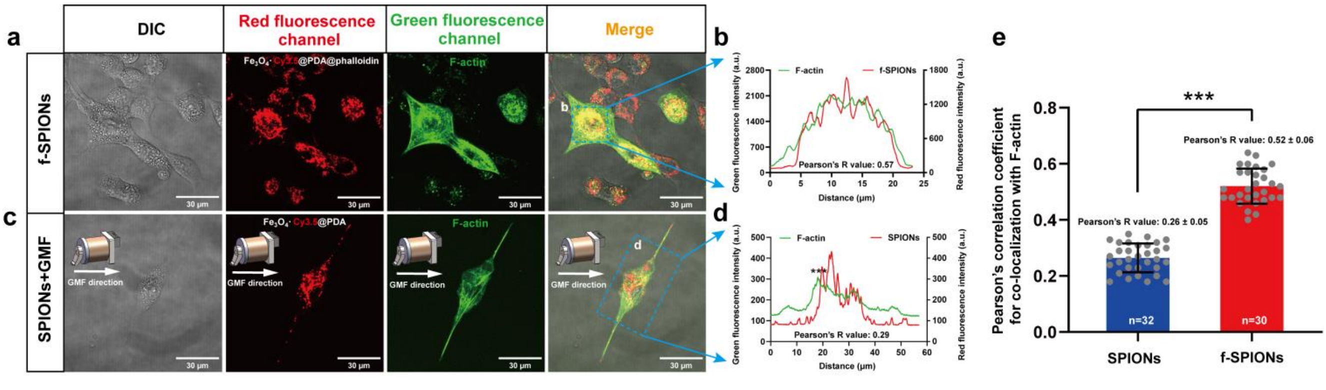

Supplementary Figure S6. Detection of ROS in SCs under magnetic stimulation mediated by f-SPIONs

a. Positive control: RSC96 cells treated with RosUp, a known ROS inducer, displayed bright green fluorescence indicating robust accumulation of intracellular ROS. The RosUp group was primarily used to calibrate and standardize the ROS fluorescence excitation parameters, ensuring the qualitative comparability of results across different groups. **b. Negative control:** RSC96 cells cultured under normal conditions presented no detectable accumulation of ROS, as indicated by the absence of green fluorescence. **c. Magnetic stimulation group:** RSC96 cells exposed to combined f-SPIONs (15 μg/mL) and GMF (3.25 T/m, 15 min per cycle with 15 min intervals, for a total of 24 cycles over 12 hours) showed no evident accumulation of ROS, indicating that the magnetic force stimulation did not induce oxidative stress. **d. f-SPIONs control group:** RSC96 cells incubated solely with f-SPIONs (15 μg/mL) for 12 hours showed no evident accumulation of ROS. **e. GMF control group:** RSC96 cells exposed solely to GMF (3.25 T/m, 15 min per cycle with 15 min intervals, for a total of 24 cycles over 12 hours) showed no evident accumulation of ROS. ***White arrows*** indicate the direction of the GMF. Each experiment was independently repeated three times.

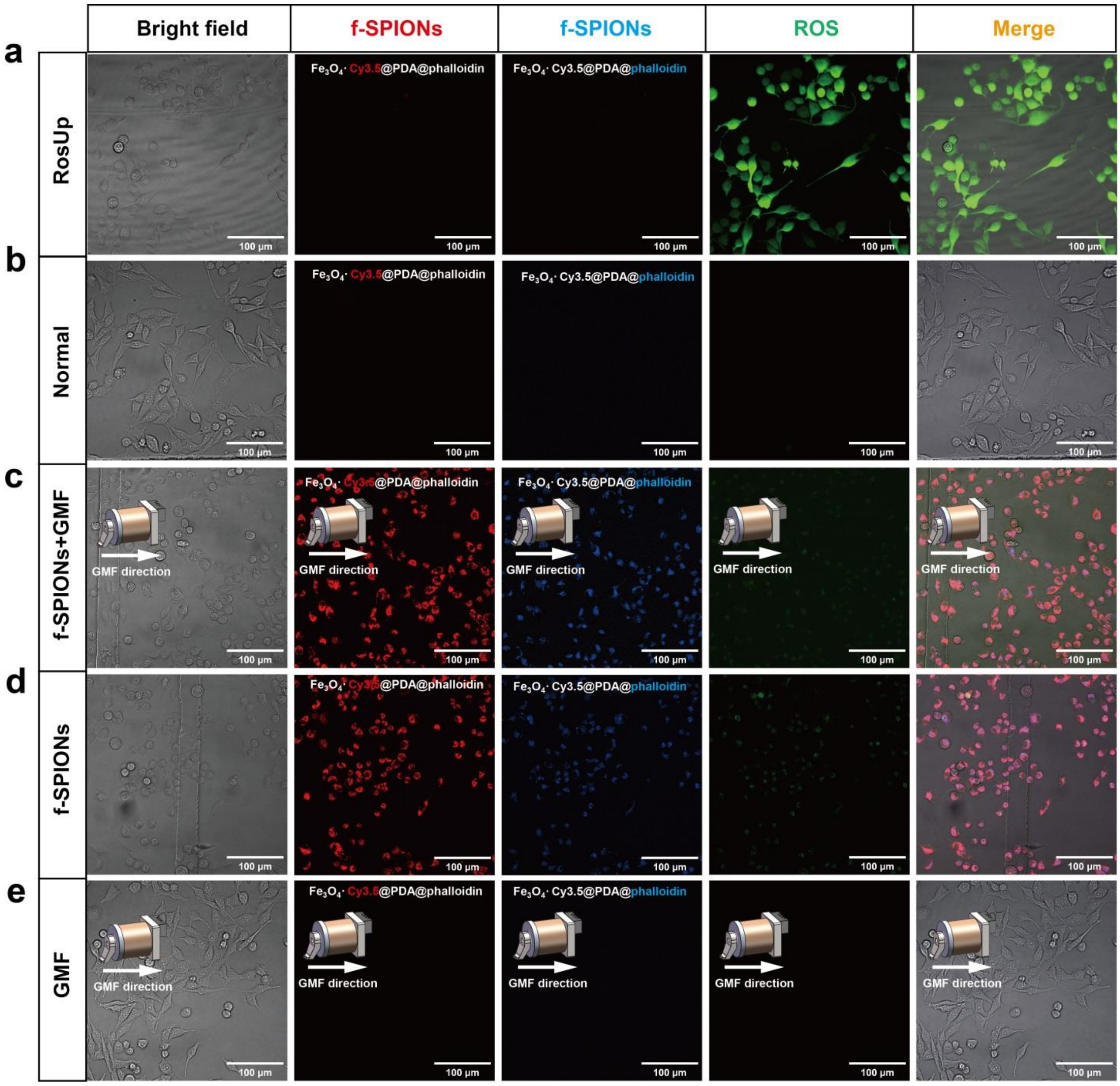

Bright field
f-SPIONs
f-SPIONs
ROS
Merge
a
b
c
d
e
RosUp
Normal
f-SPIONs+GMF
f-SPIONs
GMF
Fe3O4·Cy3.5@PDA@phalloidin
GMF direction
100 μm

Supplementary Figure S7. LPO detection in SCs under magnetic stimulation mediated by f-SPIONs

a. Positive control: RSC96 cells treated with LpoUp, an established LPO inducer, presented strong green fluorescence, indicating extensive oxidation of BODIPY 581/591 C11 and accumulation of lipid peroxidation products. The LpoUp group was primarily included to calibrate and standardize the fluorescence excitation parameters of reduced/oxidized LPO, ensuring the qualitative comparability of results among different experimental groups. **b. Negative control:** RSC96 cells cultured under standard conditions exhibited predominantly red fluorescence from BODIPY 581/591 C11, indicating that the probe remained largely in its reduced (nonoxidized) state. **c. Magnetic stimulation group:** under combined f-SPION (15 μg/mL) and GMF (3.25 T/m, 15 min per cycle with 15 min intervals, for a total of 24 cycles over 12 hours) treatment, the red/green fluorescence ratio remained comparable to that of the negative control, indicating no significant accumulation of oxidized LPO within the cells. **d. f-SPIONs control group:** RSC96 cells incubated solely with f-SPIONs (15 μg/mL) for 12 hours showed no evident accumulation of oxidized LPO. **e. GMF control group:** RSC96 cells exposed solely to GMF (3.25 T/m, 15 min per cycle with 15 min intervals, for a total of 24 cycles over 12 hours) showed no evident accumulation of oxidized LPO. ***White arrows*** indicate the direction of the GMF. Each experiment was independently repeated three times.

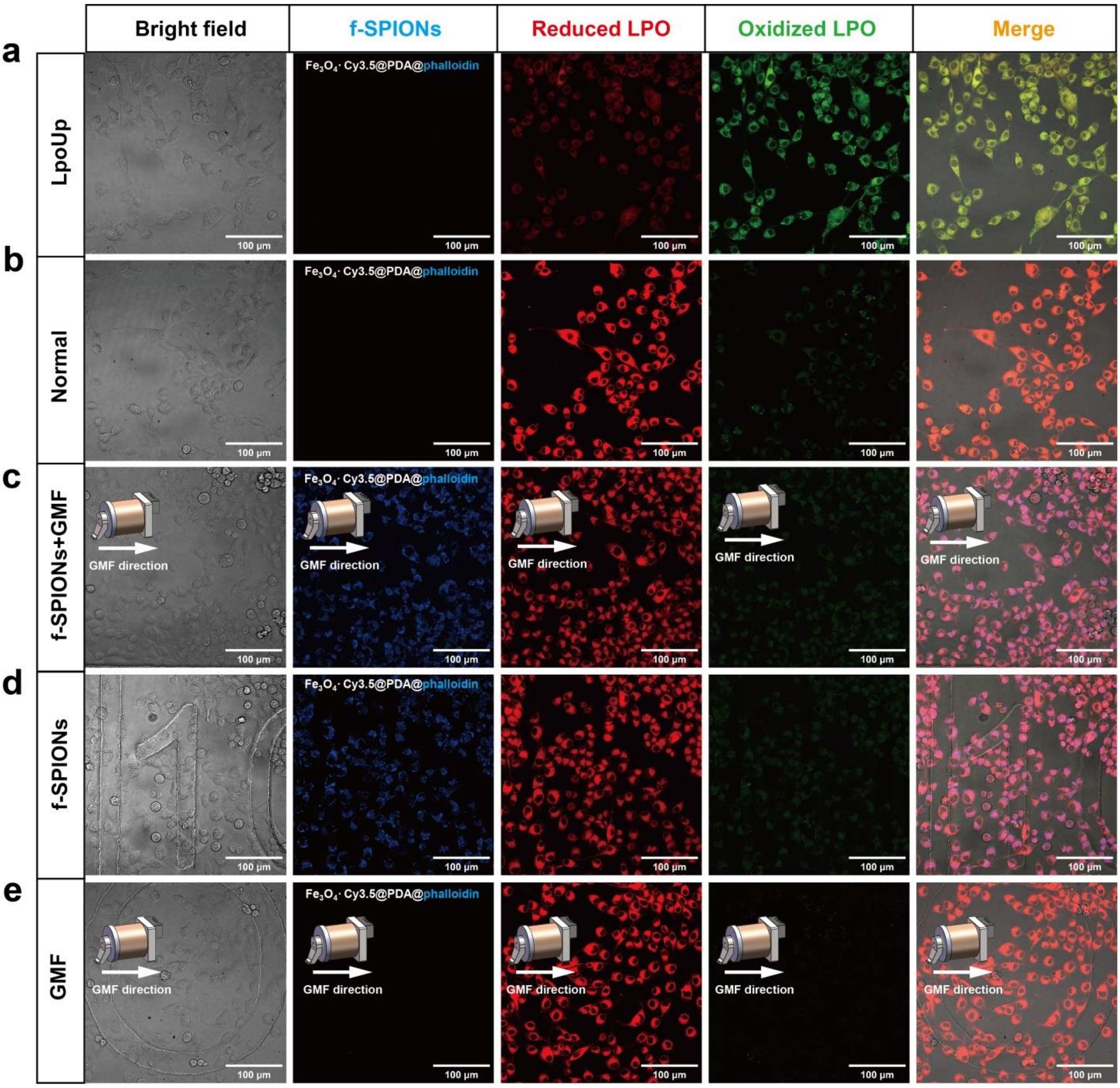
Bright field
f-SPIONs
Reduced LPO
Oxidized LPO
Merge
a
b
c
d
e
LpoUp
Normal
f-SPIONs+GMF
f-SPIONs
GMF
Fe3O4· Cy3.5@PDA@phalloidin
GMF direction
100 μm

Supplementary Figure S8. Evaluation of the cytotoxicity of magnetic stimulation using the Calcein/PI live/dead cell viability assay

a. Normal control group: RSC96 cells cultured under normal conditions. The red channel shows PI-stained dead cells, the green channel displays Calcein AM-stained live cells. **b. f-SPION-mediated magnetic stimulation group:** RSC96 cells exposed to combined treatment with f-SPIONs (15 μg/mL) and GMF (3.25 T/m, 15 min per cycle with 15 min intervals, for a total of 24 cycles over 12 hours). Red fluorescence co-localized with Calcein AM green fluorescence was identified as originating from internalized f-SPIONs, representing live cells. Red fluorescence did not co-localize with Calcein AM green fluorescence was identified as PI-stained dead cells. **c. f-SPIONs control group:** RSC96 cells incubated solely with f-SPIONs (15 μg/mL) for 12 hours. Red fluorescence co-localized with Calcein AM green fluorescence was identified as originating from internalized f-SPIONs, representing live cells. Red fluorescence did not co-localize with Calcein AM green fluorescence was identified as PI-stained dead cells. **d. GMF control group:** RSC96 cells exposed solely to GMF (3.25 T/m, 15 min per cycle with 15 min intervals, for a total of 24 cycles over 12 hours). The red channel shows PI-stained dead cells, while the green channel displays Calcein AM-stained live cells. **e.** Quantitative calculation and statistical analysis of live-cell ratios among different experimental groups. “n” indicates the number of cells observed and analyzed. Data are presented as mean ± standard deviation. White arrows indicate dead cells. Statistical significance: n.s., not significant.

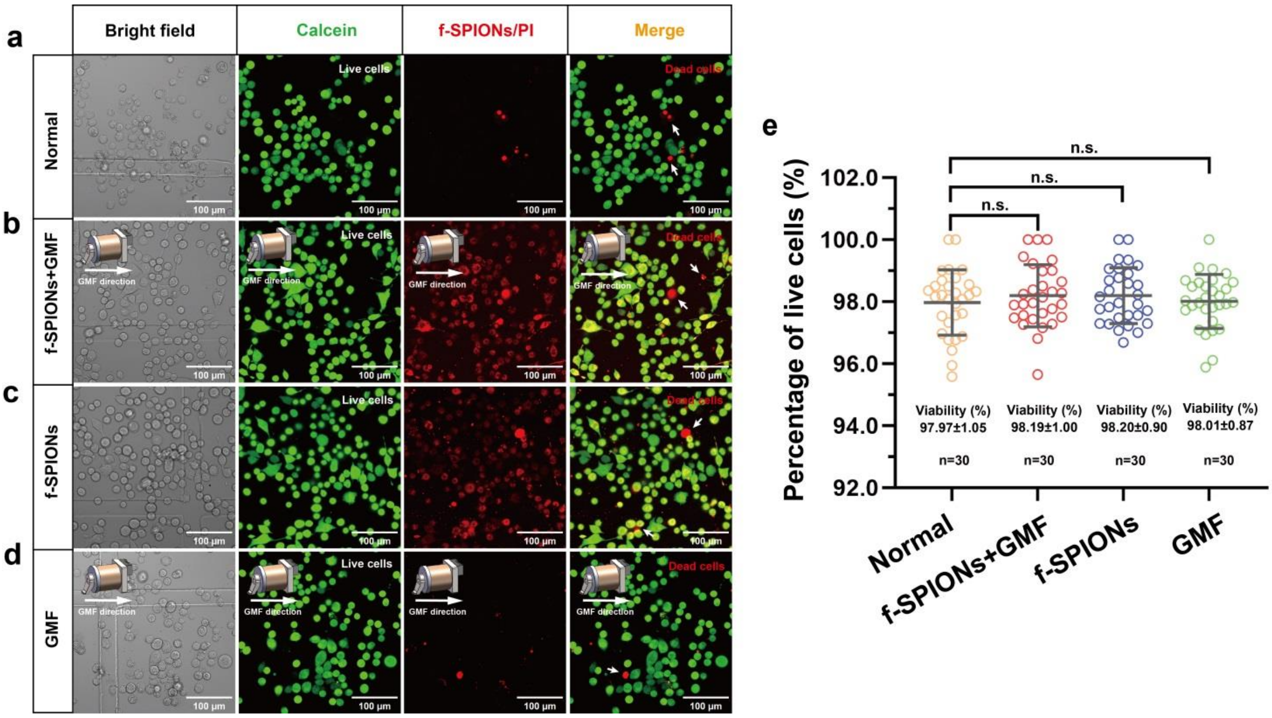
a
Bright field
Calcein
f-SPIONs/PI
Merge
Normal
Live cells
Dead cells
100 µm
b
f-SPIONs+GMF
GMF direction
c
f-SPIONs
d
GMF
e
n.s.
n.s.
n.s.
Percentage of live cells (%)
102.0
100.0
98.0
96.0
94.0
92.0
Viability (%)
97.97±1.05
98.19±1.00
98.20±0.90
98.01±0.87
n=30
Normal
f-SPIONs+GMF
f-SPIONs
GMF

Supplementary Figure S9. Evaluation of cytotoxicity of magnetic stimulation via Flow cytometry

a–c. Flow cytometric gating strategy for RSC96 cells. (a) Cell debris was excluded by FSC/SSC gating, followed by (b) single-cell gating using FSC-H vs. FSC-A plots. (c) Live and dead cell populations were distinguished using FITC fluorescence based on the Live or Dead™ staining kit. **d.** Detection of the fluorescence characteristics of f-SPIONs in cells from different experimental groups via the PE channel. **e–h.** Assessment of the live/dead cell ratio in different experimental groups via the FITC channel. **i.** Statistical analysis of the viable cell ratio across different experimental groups. "n" indicates the number of biological replicates. Data are presented as mean ± standard deviation. Statistical significance: n.s., not significant.

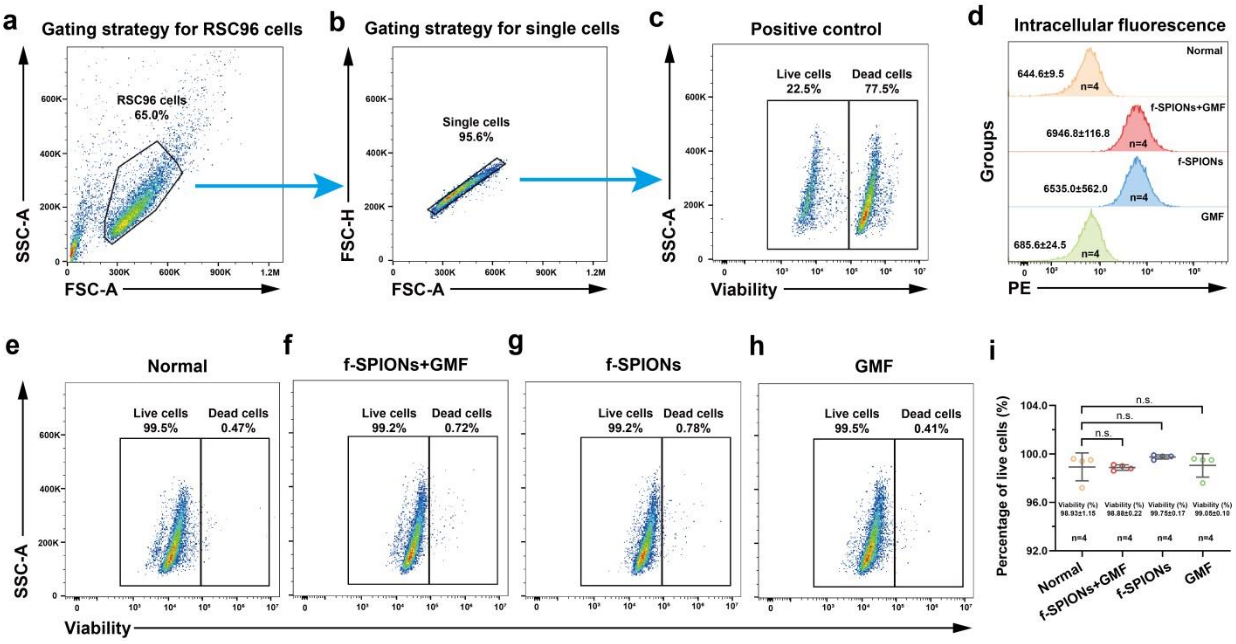

Supplementary Figure S10. Body weight monitoring of rats following systemic intravenous administration of f-SPIONs

The rats were administered f-SPIONs via intravenous injection at a dosage of 1 mg/kg. Body weight was continuously monitored for 14 consecutive days to evaluate systemic tolerance and potential physiological impact.

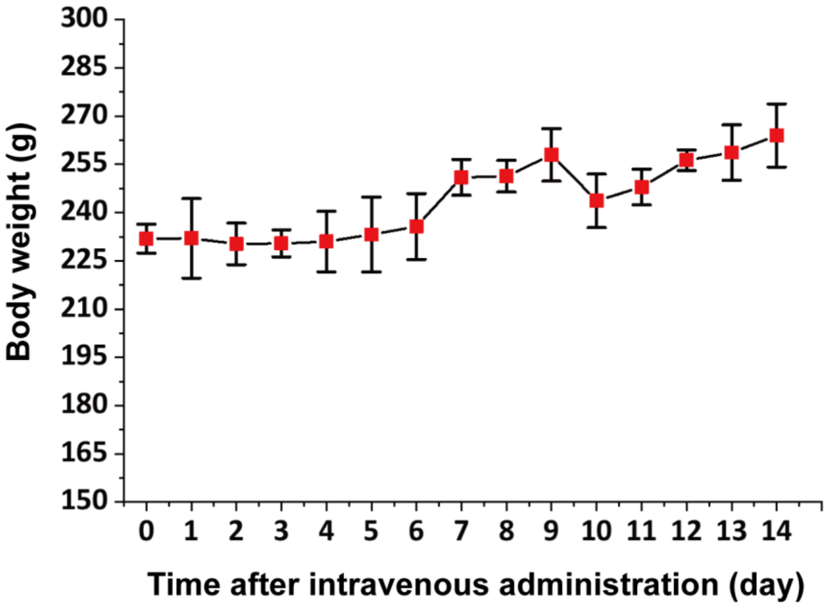

Supplementary Figure S11. Histopathological and pharmacokinetic evaluation of major organs following systemic intravenous administration of f-SPIONs

a–c. Representative histological examination (H&E staining) of (a) liver, (b) kidney, and (c) brain tissues at designated time points after intravenous administration of f-SPIONs, showing no overt pathological changes. **d–f.** The content of exogenous Fe in the (d) liver, (e) kidney, and (f) brain tissues was measured via ICP-AES at 1, 3, 7, and 14 days after intravenous administration of f-SPIONs. n = 3 rats for each group at each time point.

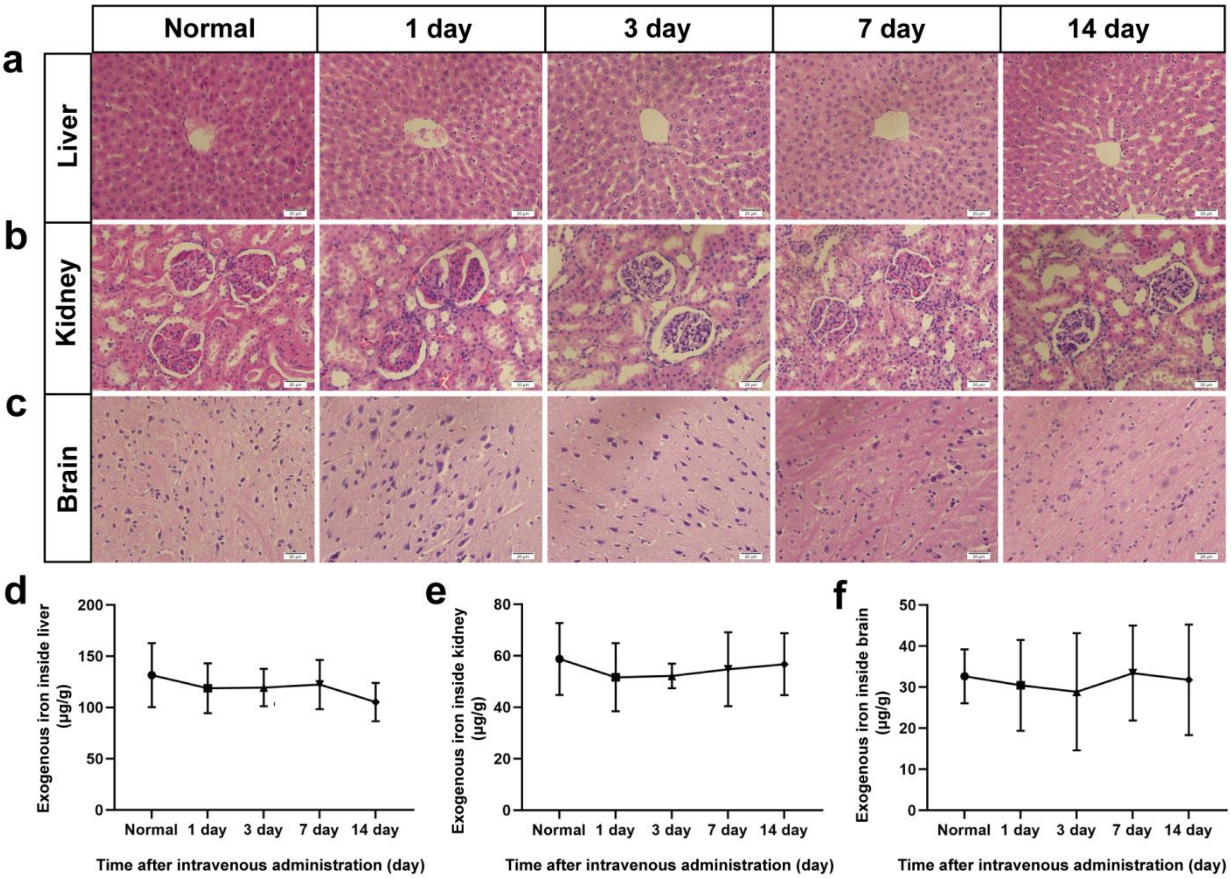

Supplementary Figure S12. F-/G-actin expression analysis in the SPIONs-mediated magnetic stimulation group

a. Western blot analysis of F-/G-actin expression in Schwann cells from the SPIONs-mediated magnetic stimulation group (SPIONs+GMF), showing results from four biological replicate experiments. **b.** Comparison of relative expression levels of G-actin and F-actin in the "SPIONs+GMF" group (using G-actin as the control group). **c.** The ratio of F-/G-actin in the SPIONs-mediated magnetic stimulation group (SPIONs+GMF) is significantly lower than that in the f-SPIONs-mediated magnetic stimulation group (f-SPIONs+GMF). * $p < 0.05$; ** $p < 0.01$.

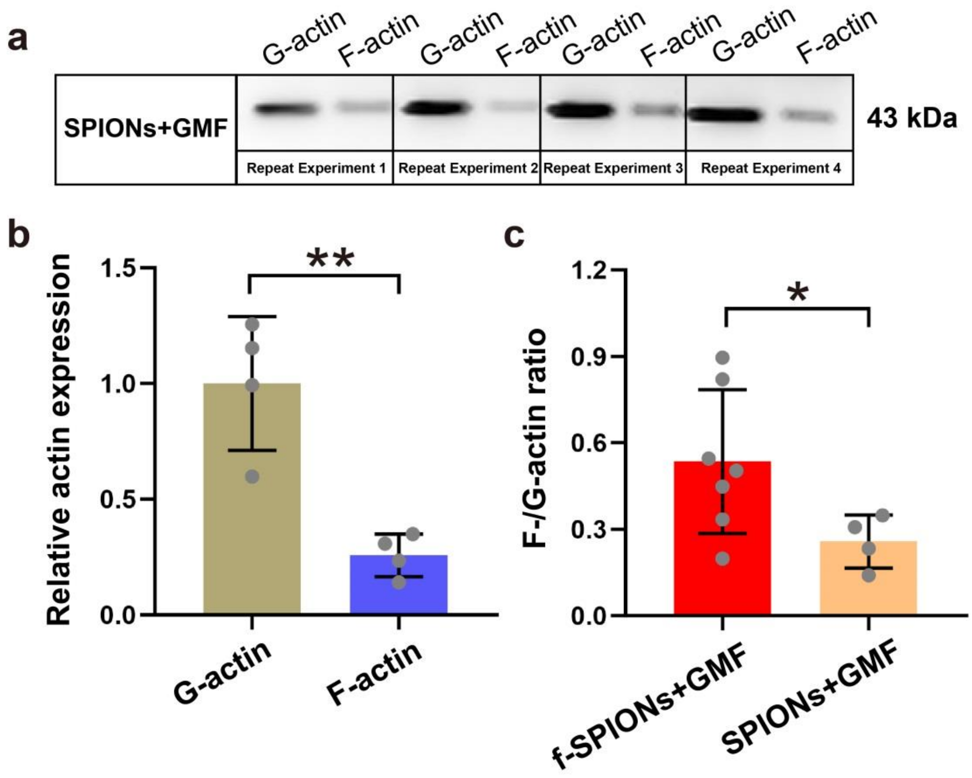

Supplementary Movie S1. Z-stack 3D reconstruction of f-SPIONs internalization by SCs

Z-stack thickness was 16.0 μm and cross-sectional imaging was performed at 1 μm intervals along the Z-axis. Imaging parameters: pinhole = 1 Airy unit, z-step = 0.43 μm. Cell membranes were stained with DiO (Ex/Em: 494/517 nm); f-SPIONs were imaged using the standard Cy3.5 excitation channel in CLSM (Ex/Em: 561–568/607 nm).

Supplementary Movie S2. Magnetomechanical stimulation via f-SPIONs induces directional neurite outgrowth

Time-lapse imaging of Schwann cells with F-actin tracing under combined stimulation with f-SPIONs and a gradient magnetic field (GMF). The cells maintained healthy morphology, exhibited active mitotic behavior, and showed directional extension of neurites and lamellipodia aligned with the magnetic field. **Time-lapse duration**: 18 hours. **Interval between frames**: 6.1 minutes. **Magnetic field exposure mode**: intermittent pulsed (15 min ON – 15 min OFF). ***White circles*** indicate mitotic cells. ***Short white arrows*** indicate directionally extended neurites and lamellipodia. ***Long white arrow*** denote the direction of the GMF.

Supplementary Movie S3. SFI gait analysis video of crush injury control and magnetic stimulation groups at 3 days post-surgery

Both the crush control and magnetic stimulation rats displayed clear hind limb (right side) dysfunction, including weak toe-off (increased footprint length) and poor toe extension

(narrower footprint width), confirming successful sciatic nerve crush modeling.

Supplementary Movie S4. SFI gait analysis video of crush injury control and magnetic stimulation groups at 7 days post-surgery

Both the crush control and magnetic stimulation rats continued to show evident hind limb (right side) dysfunction, with weak toe-off and toe extension, suggesting no significant recovery of sciatic nerve function at this stage.

Supplementary Movie S5. SFI gait analysis video of crush injury control and magnetic stimulation groups at 14 days post-surgery

The magnetic stimulation group showed marked functional recovery of the hind limb (right side) compared to the crush control group, evidenced by stronger toe-off (shorter footprint length) and toe extension (wider footprint width), indicating significant sciatic nerve recovery by day 14.

Supplementary Movie S6. Magnetomechanical stimulation via f-SPIONs activates intracellular Ca^{2+} dynamics in Schwann cells

Compared to the Normal, f-SPIONs only (f-SPIONs), and GMF only control (GMF) groups, the magnetomechanical stimulation group (f-SPIONs+GMF) exhibited significantly increased Ca^{2+} fluorescence intensity and active Ca^{2+} transients. **Time-lapse duration:** 650 seconds. **Frame interval**: 2.16 seconds. ***Yellow arrows*** indicate the direction of the GMF. ***Short white arrows*** highlight cells showing active Ca^{2+} dynamics.

Supplementary Movie S7. Activation of actin cytoskeleton dynamics regulates Ca^{2+} responses in Schwann cells

The “f-SPIONs+GMF” group showed enhanced intracellular Ca^{2+} signaling, as indicated by increased fluorescence and transient activity. Cyto D suppressed Ca^{2+} signaling by inhibiting actin polymerization. Inhibition of actin dynamics (“f-SPIONs+GMF+Cyto D” and “f-SPIONs+GMF+Anisomycin” groups) neutralized the Ca^{2+}-activating effects of magnetomechanical stimulation. **Time-lapse duration**: 650 seconds. **Frame interval**: 2.16 seconds. ***Yellow arrows*** indicate the direction of the GMF. ***Short white arrows*** highlight cells with active Ca^{2+} dynamics.

Supplementary Movie S8. Piezo1 functional activation induces intracellular Ca^{2+} dynamics in Schwann cells

GsMTx4 inhibited Piezo1 activity and significantly reduced intracellular Ca^{2+} fluorescence. Yoda1 activated Piezo1 and induced dynamic intracellular Ca^{2+} responses. The “f-SPIONs+GMF” group exhibited similar Ca^{2+} activation as Yoda1. Inhibition of Piezo1 (f-SPIONs+GMF+GsMTx4) abolished the Ca^{2+} activation effect induced by magnetomechanical stimulation. **Time-lapse duration**: 650 seconds. **Frame interval**: 2.16 seconds. ***Yellow arrows*** indicate the direction of the GMF. ***Short white arrows*** highlight cells with active Ca^{2+} dynamics.

Table S1. Chemical and peptide reagent information used in this study

Reagent or resource	Source	Identifier
Alexa Fluor™ 350 phalloidin	Invitrogen, Thermo Fisher Scientific	Cat. #A22281
CellMask™ Actin Tracking Stain	Invitrogen, Thermo Fisher Scientific	Cat. #A57249
Fluo-4 AM	Invitrogen, Thermo Fisher Scientific	Cat. #F14201
Pluronic F-127	Invitrogen, Thermo Fisher Scientific	Cat. #P3000MP
CCK-8 assay kit	Dojindo Laboratories	Cat. #CK04
ROS assay kit	Beyotime Biotechnology	Cat. #S0033S
Lipid peroxidation assay kit	Beyotime Biotechnology	Cat. #S0043S
Calcein/PI Live/Dead Viability/Cytotoxicity Assay Kit	Beyotime Biotechnology	Cat. #C2015S
Annexin V-APC/7-AAD Apoptosis Detection Kit	Lianke Bio	Cat. # AP105
Live or Dead™ Fixable Dead Cell Staining Kit	AAT Bioquest	Cat. #CS22501
Cell Plasma Membrane Staining Kit with DiO	Beyotime Biotechnology	Cat. # C1993S
G-Actin/F-Actin In Vivo Assay Kit	Cytoskeleton, Inc.	Cat. #BK037
Cytochalasin D	Invitrogen	Cat. #PHZ1063
Anisomycin	MCE	Cat. #HY-18982
GsMTx4	MCE	Cat. #HY-P1410A
Yoda1	MCE	Cat. #HY-18723
TRIzol reagent	Thermo Fisher Scientific	Cat. #15596018
PrimeScript RT Master Mix kit	Takara Bio	Cat. #RR036A
PowerUp SYBR™ Green Master Mix Kit	Thermo Fisher Scientific	Cat. #A25742
Radioimmunoprecipitation assay buffer	Beyotime Biotechnology	Cat. #P0013B
Bicinchoninic acid (BCA) protein assay kit	Thermo Fisher Scientific	Cat. #23225
Polyvinylidene difluoride membranes	Millipore	Cat. #IPVH00010
Enhanced chemiluminescence (ECL) detection system	Millipore	Cat. #WBKLS0500
Goat serum	Dako, Agilent Technologies	Cat. #X0907
RNA 6000 Nano LabChip Kit	Agilent Technologies	Cat. #5067-1511
Dynabeads Oligo (dT)	Thermo Fisher Scientific	Cat. #61005
Magnesium RNA Fragmentation Module	New England Biolabs	Cat. #E6150
SuperScript™ II Reverse Transcriptase	Invitrogen, Thermo Fisher Scientific	Cat. #1896649
E. coli DNA Polymerase I	New England Biolabs	Cat. #M0209
RNase H	New England Biolabs	Cat. #M0297
dUTP solution	Thermo Fisher Scientific	Cat. #R0133
AMPure XP magnetic beads	Beckman Coulter	Cat. #A63880
Heat-labile uracil-DNA glycosylase	UDG; New England Biolabs	Cat. #M0280

Table S4. Sequences of the primers used in this study

Primer	Forward primer (5'-3')	Reversed primer (5'-3')
Piezo1	TCATTGTGGTCAGCTTCGCT	GAGCCACCAGAAAACTCGGA
c-Jun	CGCCAACCTCAGCAACTTCA	CGTCTGCGGCTCTTCCTTC
STAT3	TTAACATTCTGGGCACGAACA	CACCACGACTGGCAAGGAG
NCAM1	ATCCACCTCAAGGTCTTCGC	TCACTGCTGATGTTTCGGGT
integrin β1	CCAAGTGGGACACGGGTGAA	TTCCCTCATACTTCGGATTGACCAC
GAPDH	ACGGCAAGTTCAACGGCACAG	GACGCCAGTAGACTCCACGACA

Table S5. Antibody reagent information used in this study

Antibodies	Working concentration	Source	Identifier	Application
Rabbit polyclonal anti-Piezo1 antibody	1:1000	Alomone Labs	Cat. #APC-087	Western blot
Rabbit monoclonal anti-c-Jun antibody	1:1000	Abcam	Cat. #ab40766	Western blot
Rabbit monoclonal anti-STAT3 antibody	1:1000	Abcam	Cat. #ab68153	Western blot
Rabbit monoclonal anti-NCAM antibody	1:1000	Abcam	Cat. #ab220360	Western blot
Rabbit monoclonal anti-Integrin β1 antibody	1:1000	Abcam	Cat. #ab179471	Western blot
Mouse monoclonal anti-GAPDH antibody	1:1000	Proteintech	Cat. #60004-1-Ig	Western blot
Horseradish peroxidase (HRP)-conjugated goat anti-rabbit IgG (H+L)	1:1000	Beyotime Biotechnology	Cat. #A0208	Western blot
Horseradish peroxidase (HRP)-conjugated goat anti-mouse IgG (H+L)	1:5000	Thermo Fisher Scientific	Cat. #31431	Western blot
Rabbit polyclonal anti-Piezo1 antibody	1:1000	Thermo Fisher Scientific	Cat. #PA5-116998	Immunofluorescence
Rabbit polyclonal anti-Laminin β1 antibody	1:500	Proteintech	Cat. #23498-1-AP	Immunofluorescence
Alexa Fluor 488-conjugated goat anti-rabbit IgG	1:200	Invitrogen	Cat. #A-11008	Immunofluorescence
